# Obesogenic eating behaviour and dietary intake in German children and adolescents: results from the GINIplus and LISA birth cohort studies

**DOI:** 10.1038/s41430-022-01125-2

**Published:** 2022-04-01

**Authors:** Anne Marb, Lars Libuda, Marie Standl, Sibylle Koletzko, Carl-Peter Bauer, Tamara Schikowski, Dietrich Berdel, Andrea von Berg, Gunda Herberth, Judith Bühlmeier, Carla P. Harris

**Affiliations:** 1 Institute of Epidemiology, Helmholtz Zentrum München GmbH, German Research Centre for Environmental Health, Neuherberg, Germany; 2 Institute for Medical Information Processing, Biometry and Epidemiology - IBE, LMU Munich, Munich, Germany; 3 Pettenkofer School of Public Health, Munich, Germany; 4 Department of Child and Adolescent Psychiatry, University Hospital Essen, University of Duisburg-Essen, Essen, Germany; 5 Children’s Hospital, Evangelisches Krankenhaus Düsseldorf, Düsseldorf, Germany; 6 Institute of Nutrition, Consumption and Health, Faculty of Natural Sciences, Paderborn University, Paderborn, Germany; 7 Dr. von Hauner Children’s Hospital, University Hospital, LMU Munich, Munich, Germany; 8 Department of Pediatrics, Gastroenterology and Nutrition, School of Medicine Collegium Medicum University of Warmia and Mazury, Olsztyn, Poland; 9 Department of Pediatrics, Technical University of Munich, Munich, Germany; 10 IUF-Leibniz Research Institute for Environmental Medicine, Düsseldorf, Germany; 11 Research Institute, Department of Pediatrics, Marien-Hospital Wesel, Wesel, Germany; 12 Department of Environmental Immunology, Helmholtz Centre for Environmental Research - UFZ, Leipzig, Germany

**Keywords:** Risk factors, Obesity, Nutrition, Epidemiology

## Abstract

**Background/objectives:**

The transition to adolescence is characterised by considerable behavioural changes, including diet. This study describes the level of obesogenic eating behaviours in 10- and 15-year-olds, and their association with dietary intake.

**Subjects/methods:**

Participants of the 10- and 15-year follow-ups of the German GINIplus and LISA birth cohort studies were included (N_10_ = 2257; N_15_ = 1880). Eating behaviours and dietary intake were assessed via self-report questionnaires. Sex-stratified, cross-sectional associations of “external eating”, “emotional eating” and “dietary restraint” (the latter at age 15 years only) with dietary intake (17 food groups—categorised into tertiles, macronutrients, and total energy) were assessed using multinomial logistic or multiple linear regression as required, adjusting for covariates and correcting for multiple testing.

**Results:**

Reported levels of eating behaviours were low in both age-groups. External eating was higher in 10-year-old males than females, while all eating behaviours were most pronounced in 15-year-old females. At 10 years, emotional eating was associated with medium vegetable intake in females (Relative Risk Ratio (RRR) = 1.84, *p* = 0.0017). At 15 years, external eating was associated with total energy (kJ) in females (*β* = 718, *p* = 0.0002) and high butter intake in males (RRR = 1.96, *p* = 0.0019). Dietary restraint in females was inversely associated with total energy (*β* = −967, *p* < 0.0001) and omega-3 fatty acids (Means Ratio (MR) = 0.94, *p* = 0.0017), and positively associated with high fruit (RRR = 2.20, *p* = 0.0003) and whole grains (RRR = 1.94, *p* = 0.0013).

**Conclusion:**

Obesogenic eating behaviour scores are low among children and adolescents of a predominantly high socioeconomic status population and present only few associations with specific aspects of diet, mainly among adolescent females.

## Introduction

Overweight and obesity among children and adolescents are major public health issues, impairing health and overall quality of life [1, 2]. In Germany, around 15% of children and adolescents are currently affected [3]. The aetiology of childhood obesity is multifactorial and complex [4]; however, diet is a known risk factor, e.g. through energy imbalance caused by overeating [5]. Acquired behaviours often have a strong influence on eating decisions compared to biological mechanisms, i.e. internal stimuli triggering hunger and satiety [6–8]. In particular, three eating behaviours are associated with low responsiveness to internal signals of food intake: external eating, which refers to a high susceptibility to external food cues [9]; emotional eating, implying food intake as a coping strategy for emotional distress [9]; and dietary restraint, the cognitive regulation of eating for weight control, often observed in individuals struggling to maintain control over their food intake and weight [10, 11]. These behavioural dimensions have been associated with overweight among children and adolescents [12–14]. Understanding what aspects of the diet are influenced by these behaviours is of major importance for nutritional education and obesity prevention. The period of pubertal development is of special interest, given the shift in diet, being highly controlled by parents at younger ages, to increased autonomy, influence of peers as well as body image and weight concerns in adolescence [15, 16]. Studies on the association of eating behaviours with diet in children and adolescents have reported mixed results [15, 17–22]. These predominantly focus on single aspects of eating behaviour [15, 17, 19–22] and dietary intake, the latter mostly limited to selected (obesity-related) food groups or nutrients [15, 18, 20–22]. This study aims to describe the levels of external eating, emotional eating and dietary restraint at two time-points representing late childhood and adolescence, and their cross-sectional association with usual dietary intake among participants of the population-based German birth cohorts, GINIplus and LISA.

## Subjects and methods

### Study participants

The study included participants from the 10- and 15-year follow-ups of two ongoing prospective German birth cohort studies, GINIplus (German Infant Nutritional Intervention plus environmental and genetic influences on allergy development) and LISA (Influence of Lifestyle related factors on the development of the Immune System and Allergies in East and West Germany). In GINIplus, 5991 healthy full-term newborns were recruited from obstetric clinics in Munich and Wesel, between 1995 and 1998. Newborns with a family history of atopic disease were invited for the intervention arm, investigating effects of different hydrolysed formulae on allergy development. All others, and those declining participation in the intervention, were invited for the observation arm. The LISA cohort included 3097 healthy full-term newborns recruited between 1997 and 1999 in Munich, Wesel, Leipzig and Bad Honnef. In both studies, participants underwent assessments at regular follow-ups. Details on study design, recruitment strategy and exclusion criteria have been described previously [23, 24]. Both studies were conducted in accordance with the ethical standards laid down in the Declaration of Helsinki and approved by the local ethics committees (Bavarian Board of Physicians, Medical Faculty of the University of Leipzig, Board of Physicians of Saxony, and Board of Physicians of North-Rhine-Westphalia). All participants and their families gave written informed consent.

### Eating behaviour

Eating behaviour was assessed by the Eating Behaviour and Weight Problems Inventory for Children (EWI-C) [25], designed specifically for children and young adolescents and widely applied in other European countries [26]. At 10 and 15 years, the inventory was addressed directly to the participants. It includes 60 items assigned to 10 subscales, of which three were integrated in the GINIplus and LISA studies: “External eating” (subscale 1), “Emotional eating” (subscale 3), and “Dietary restraint” (subscale 5). While the first two subscales were assessed at both follow-ups, “Dietary restraint” was not assessed at the 10-year follow-up, as it was considered that the emphasis on food avoidance as a means for weight reduction, could potentially transfer negative beliefs, attitudes and behaviours related to weight and body image to the young participants. We assumed that at 10 years, participants may be more prone to adopt new attitudes and behaviours that could lead to later disordered eating, while such behaviours are likely to be already established in adolescence [27]. Each item is scored on a four-point Likert scale (0 = not at all, 1 = little, 2 = mostly, 3 = totally), and these are summed up to obtain subscale scores (subscale 1 and 3 (8 items each): score = 0–24, subscale 5 (7 items): score = 0–21). Given the highly skewed distribution of the EWI-C scores, these were categorised into age- and sex-specific tertiles (T1 = low, T2 = medium, T3 = high).

### Dietary intake

Dietary intake was assessed at the follow-ups age 10- and 15-years by a self-completed food frequency questionnaire (FFQ) containing 80 food items, designed and validated to estimate usual food intake in school-aged children [28]. At 10 years, parents were asked to complete the FFQ alongside their children, whereas at 15 years participants were addressed directly. Reported consumption frequency and portion sizes were converted into average daily intakes (g/d). Corresponding energy and nutrient contents were calculated using the German Food Code and Nutrient Database (BLS), version II.3.1 [29]. As part of the data quality control procedure, participants were excluded if a complete block of food items was empty or more than 40 food items (50 % of the FFQ) were missing. To further reduce the risk of under- and over-reporting of food intake, participants were excluded if total daily energy intake was outside 500–3500 kcal (2093–14,654 kJ) for females or 800–4000 kcal (3349–16,747 kJ) for males [30]. Moreover, exclusions were made if provided values for %EI of specific food items were implausible (outliers visually detected by means of boxplots). Further information on the dietary assessment and quality control steps is provided in Supplementary Text S1. Foods were classified into 17 food groups [31]. The present analysis includes all food groups, total daily energy intake, and macronutrients: fat, protein, carbohydrate, fibres, total sugar (mono- and disaccharides), saturated fatty acids (SFA), monounsaturated fatty acids, polyunsaturated fatty acids (PUFA), and omega-3 and −6 PUFA. Food groups and nutrients are expressed as their percentage contribution towards total daily energy intake (%EI), except water and tea (ml/day), fibres (g/day) and total energy intake (kJ/day). Given the skewed distribution of many food groups, these were categorised into tertiles (T1 = low, T2 = medium, T3 = high). Nutrients and total energy intake were treated as continuous variables, with total, omega-3, and omega-6 PUFA naturally log-transformed due to their log-normal distribution.

### Covariates

Covariates were selected based on existing literature or if relevant to the study design. Those correlated in the univariate analysis (*p* < 0.05) with any exposure or outcome were kept: exact age at dietary assessment; body mass index (BMI); pubertal status, defined at 10 years as pubertal onset (yes, no), and at age 15 years as pubertal stage (pre-mid, late, or post-pubertal); siblings (yes, no); moderate-to-vigorous physical activity (low, medium, high); screen time (low, high); total difficulties, assessed by the Strengths and Difficulties Questionnaire [32–34] (normal, borderline, abnormal); parental education (low-medium, high), parental BMI (kg/m²); study (GINIplus observation, GINIplus intervention, LISA), recruitment region (Munich, Leipzig, Bad Honnef, Wesel), and total daily energy or beverage intake. At both follow-ups, information on covariates was obtained through medical examination or questionnaires. Details on the assessment and categorisation of covariates are provided in Supplementary Table S1.

### Statistical analysis

**Fig. 1:**
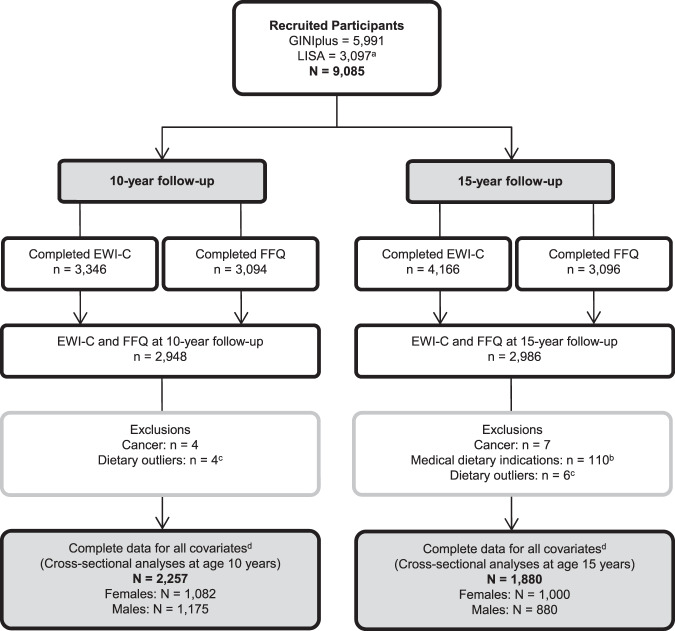
Flowchart study population. EWI-C Eating Behaviour and Weight Problems Inventory for Children, FFQ food frequency questionnaire. ^a^Three families removed their consent to participate in the study. ^b^Medical dietary indications included diabetes, coeliac disease, food allergies, food intolerances, and were assessed only at the 15-year follow-up. ^c^Dietary outliers: Clear outliers in diet variables were visually identified using descriptive plots and excluded from the analysis. ^d^Covariates: age, BMI, pubertal status, siblings, moderate-to-vigorous physical activity, screen time, total difficulties, parental education, parental BMI, study, recruitment region, and total energy or total beverage intake.

All statistical analyses were performed separately at each follow-up and stratified by sex, using R version 4.0.3 [35] (code available on request). Participants reporting cancer or medical dietary indications (reported only at 15 years), and those presenting dietary outliers (visually identified using boxplots) or with incomplete data for covariates were excluded (Fig. 1). Subject characteristics were described by medians (25th percentile; 75th percentile) or counts (%). Differences between sexes were tested by Wilcoxon’s rank sum test for continuous variables, and Pearson’s χ² test for categorical variables. Cross-sectional associations between eating behaviours and dietary intake were assessed by multinomial logistic regression for categorical outcome variables (ordinal logistic regression was not applied as the proportional odds assumption, tested using Brant test [36], was not fulfilled), and multiple linear regression for continuous outcome variables. All models were adjusted for the above-mentioned covariates. Total energy intake was included in all models except those with water and tea, which included total beverage intake. Results of multinomial logistic regression are presented as relative risk ratios (RRR), and of linear regression as beta coefficients (β) or means ratios (MR) for naturally log-transformed outcome variables, each with corresponding 95% confidence intervals (95% CI). Multinomial logistic regression was calculated using the “multinom” function in the R package “nnet” [37]. We corrected for multiple testing according to Nyholt [38]. Making use of the correlation pattern among all outcome variables, this method derives the number of effective variables, hence providing an estimate of the number of independent tests. The α-level was divided by this number, yielding a two-sided α-level of 0.0019 (0.05/26 = 0.0019). We performed various sensitivity analyses: First, excluding participants who reported a vegetarian or vegan diet. Second, we excluded participants with a BMI < 10th or >90th percentile. In the remaining sub-population, we further tested the interaction of each eating behaviour with BMI to detect possible BMI-specific effects. Significant interactions (*p* < 0.0019) were visualised applying the “plot_model” function in the R package “sjPlot” [39].

## Results

### Study population

The study population included 1082 females and 1175 males at the 10-year follow-up, and 1000 females and 880 males at the 15-year follow-up (Fig. 1). Basic characteristics of the study population at each follow-up are described in Table 1. Median external eating scores averaged 6–7 at both time-points, with highest scores switching from males at 10 years to females at 15 years. The median score for emotional eating at 10 years was 1 in both sexes. At 15 years, both emotional eating and dietary restraint were 3 in females and 1 in males. Descriptive statistics of food group and nutrient intakes can be found in Supplementary Tables S2 and S3. Overall, females consumed more fruit and vegetables, while males consumed more meat and caloric drinks.

**Table 1:** Descriptive characteristics of study population at the 10-year and 15-year follow-up.

		10-year follow-up		*P* value^a^	15-year follow-up		*P* value^a^
		Females (*N* = 1082)	Males (*N* = 1175)		Females (*N* = 1000)	Males (*N* = 880)	
Age [years]		10.7 (10.5;11.2)	10.7 (10.4;11.1)	**0.045**	15.4 (15.2;15.7)	15.4 (15.2;15.7)	0.537
BMI [kg/m²]		16.6 (15.5;18.4)	16.7 (15.6;18.4)	0.624	20.2 (18.7;21.9)	19.9 (18.4;21.9)	0.029
10th percentile > BMI > 90th percentile	Yes	218 (20.1)	236 (20.1)	1.000	200 (20.0)	176 (20.0)	1.000
	No	864 (79.9)	939 (79.9)		800 (80.0)	704 (80.0)	
Total energy intake [kJ/d]		7401 (6107;8818)	8,633 (7082;10,254)	**<0.001**	7204 (5722;9007)	9721 (7796;11,708)	**<0.001**
Vegetarian or vegan diet	Yes	10 (0.9)	4 (0.3)	0.135	54 (5.4)	14 (1.6)	**<0.001**
	No	1072 (99.1)	1171 (99.7)		946 (94.6)	866 (98.4)	
Puberty onset	Yes	510 (47.1)	139 (11.8)	**<0.001**	–	–	
	No	572 (52.9)	1036 (88.2)		–	–	
Puberty stage	Pre-mid	–	–		38 (3.8)	357 (40.6)	**<0.001**
	Late	–	–		799 (79.9)	516 (58.6)	
	Post	–	–		163 (16.3)	7 (0.8)	
Siblings	Yes	960 (88.7)	1043 (88.8)	1.000	885 (88.5)	775 (88.1)	0.827
	No	122 (11.3)	132 (11.2)		115 (11.5)	105 (11.9)	
Moderate-vigorous PA	Low	306 (28.3)	210 (17.9)	**<0.001**	282 (28.2)	147 (16.7)	**<0.001**
	Medium	574 (53.0)	637 (54.2)		548 (54.8)	474 (53.9)	
	High	202 (18.7)	328 (27.9)		170 (17.0)	259 (29.4)	
Screen time	Low	989 (91.4)	1020 (86.8)	**0.001**	531 (53.1)	336 (38.2)	**<0.001**
	High	93 (8.6)	155 (13.2)		469 (46.9)	544 (61.8)	
Total difficulties	Normal	970 (89.6)	987 (84.0)	**<0.001**	932 (93.2)	841 (95.6)	0.086
	Borderline	50 (4.6)	86 (7.3)		58 (5.8)	33 (3.8)	
	Abnormal	62 (5.7)	102 (8.7)		10 (1.0)	6 (0.7)	
Parental education	Low-medium	304 (28.1)	393 (33.4)	**0.007**	270 (27.0)	261 (29.7)	0.220
	High	778 (71.9)	782 (66.6)		730 (73.0)	619 (70.3)	
Parental BMI	Normal	408 (37.7)	454 (38.6)	0.638	398 (39.8)	341 (38.8)	0.398
	Overweight	504 (46.6)	525 (44.7)		401 (40.1)	378 (43.0)	
	Obese	170 (15.7)	196 (16.7)		201 (20.1)	161 (18.3)	
Study (arm)	GINI observation	417 (38.5)	402 (34.2)	**0.040**	368 (36.8)	303 (34.4)	**0.043**
	GINI intervention	301 (27.8)	322 (27.4)		285 (28.5)	223 (25.3)	
	LISA	364 (33.6)	451 (38.4)		347 (34.7)	354 (40.2)	
Region	Munich	550 (50.8)	594 (50.6)	0.819	536 (53.6)	503 (57.2)	0.104
	Leipzig	71 (6.6)	85 (7.2)		71 (7.1)	77 (8.8)	
	Bad Honnef	48 (4.4)	59 (5.0)		47 (4.7)	35 (4.0)	
	Wesel	413 (38.2)	437 (37.2)		346 (34.6)	265 (30.1)	
External eating^b^		6 (4; 9)	7 (4; 10)	**0.004**	7 (4; 10)	6 (4; 10)	**0.010**
Emotional eating^b^		1 (0; 3)	1 (0; 3)	0.496	3 (1; 6)	1 (0; 4)	**<0.001**
Dietary restraint^b^		–	–		3 (1; 8)	1 (0; 3)	**<0.001**

Significant differences marked in bold: *p* < 0.05.

*BMI* body mass index, *PA* physical activity.

^a^Comparison between males and females: tested by Wilcoxon’s rank sum test for continuous variables, and by Pearson’s χ² test for categorical variables.

^b^Theoretical range of score values: external eating score = 0–24; emotional eating score = 0–24; dietary restraint score = 0–21. Values are presented as counts (%) for categorical variables and medians (25th; 75th percentile) for continuous variables.

### Association between eating behaviour and dietary intake

**Fig. 2:**
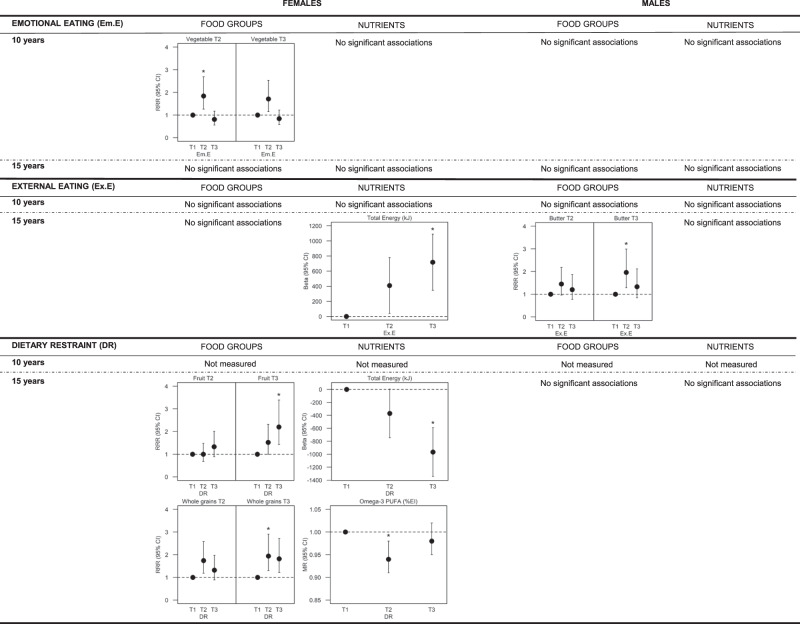
Significant associations between tertiles of eating behaviours and dietary intake. Beta beta coefficient, CI confidence interval, MR means ratio, PUFA polyunsaturated fatty acids, RRR relative risk ratio; T1 tertile 1, T2 tertile 2, T3 tertile 3. Effect estimates of multinomial logistic regression are presented as relative risk ratio (95% CI). Effect estimates of multiple linear regression are presented as beta coefficient (95% CI). Effect estimates of multiple linear regression for naturally log-transformed outcome variables are presented as means ratio (95% CI). All models were adjusted for age, BMI, pubertal status, siblings, moderate-to-vigorous physical activity, screen time, total difficulties, parental education, parental BMI, study, and recruitment region. Food groups (except water and tea) and nutrients models were further adjusted for total daily energy intake. Water and Tea models were further adjusted for total daily beverage intake. Tertile 1 is the reference category. *Statistically significant (*p* < 0.0019).

Results of regression analyses on the associations of eating behaviour and dietary intake in females and males at 10 and 15 years are presented in Fig. 2 (significant associations) and in Supplementary Tables S4–7. The first tertile (T1 = low) is the reference level for both exposure and categorical outcome variables.

#### External eating

External eating was not associated with dietary intake in females or males at 10 years. At 15 years, high external eating levels were linked to increased total energy intake in females (*β* = 718, 95% CI = (346;1090), *p* value = 0.0002) (Supplementary Table S6), and medium levels were positively associated with high butter intake in males (RRR = 1.96 (1.28; 2.99), *p* = 0.0019) (Supplementary Table S7).

#### Emotional eating

At the follow-up at age 10 years, medium levels of emotional eating were linked to medium vegetable intakes in females (RRR = 1.84 (1.26;2.69), *p* = 0.0017) (Supplementary Table S4). No association was found in males at the age of 10 years, nor at the age of 15 years in either sex.

#### Dietary restraint (15-year follow-up)

In females, high levels of dietary restraint were associated with high fruit intake (RRR = 2.20 (1.43;3.39), *p* = 0.0003) and lower total energy intake (*β* = −967 (−1,343; −591), *p* < 0.0001). Further, females with medium levels of dietary restraint were more likely to report high whole grain intakes (RRR = 1.94 (1.30;2.91), *p* = 0.0013), and lower omega-3 PUFA intakes (MR = 0.94 (0.91;0.98), *p* = 0.0017) (Supplementary Table S6). No associations with dietary restraint were observed in males.

After excluding participants who reported vegetarian or vegan diets (Supplementary Tables S8–11), the association of dietary restraint with fruit and whole grain intake in 15-year-old females was diminished (Supplementary Table S10).

### Effect modification by BMI

Excluding participants with BMI < 10th or >90th percentile altered some associations (Supplementary Table S12–15): in females, the associations of emotional eating with vegetable intake at 10 years, and of external eating and dietary restraint with energy intake at 15 years remained significant; and a new positive association was observed at 15 years between emotional eating and medium water intake. In 15-year-old males, the association between external eating and butter was not significant, and an additional association with medium tea intake was observed. Significant interactions with BMI were observed for dietary restraint (with whole grain, oils and sugar-sweetened foods) and emotional eating (with dairy). These are depicted in plots of marginal effects stratified by BMI (low: <25th percentile; medium: ≥25th and <75th percentile; high: ≥75th percentile) in Supplementary Fig. S1. We observed positive associations among low-BMI participants and inverse associations among high-BMI participants with high dairy (10-year-old males) and high oil (15-year-old males). Different effect sizes with similar direction were observed for whole grains (15-year-old females) and sugar-sweetened foods (15-year-old males).

## Discussion

The present analyses examined the extent of obesogenic eating behaviours (external eating, emotional eating, and dietary restraint) and their cross-sectional association with dietary intake, among 10- and 15-year-olds from the German GINIplus and LISA birth cohort studies. At both ages, external eating scores were higher than other eating behaviour scores. Males presented higher levels of external eating at 10 years, while all eating behaviours were more pronounced in females at the age of 15 years. Associations with dietary intake were mainly observed at the age of 15 years and among females. The most robust associations following sensitivity analyses were observed in females for emotional eating with vegetable intake at age 10 years, and for external eating and dietary restraint with total energy intake at 15 years.

### Extent of obesogenic eating behaviour

In our study, the three EWI-C subscales ranged from median = 1–7, notably below those reported by Diehl (ranging 5–12) [25] in a representative sample of German schoolchildren (*n* = 923, 11–16 years) based on which the EWI-C was developed. Reasons for this discrepancy might be the overrepresentation of children from higher socioeconomic status, with a low prevalence of overweight and mental health problems in the GINIplus and LISA cohorts due to non-random loss-to-follow-up [24, 40]. However, our findings largely agree concerning sex and age differences: the increase in emotional eating levels from 10 to 15 years in females may be related to increased emotion-related problems such as anxiety, peer harassment or depression, which are often more present in adolescence [41, 42] and among females [43]. Furthermore, body weight and image dissatisfaction are also more common in females [13], reflected by their significantly higher score for dietary restraint. In males, however, impulsivity and less inhibitory control towards food cues might explain a higher score for external eating at 10 years [44, 45]. The observed increase in scores for all EWI-C subscales with age in our study may be related to the switch from a highly controlled child’s diet towards a more autonomous eating behaviour in adolescence [16]. On the other hand, at 10 years, children likely received support from their parents in completing the questionnaire, who possibly were not aware of or did not admit the extent of their child’s eating behaviour, leading to underreporting.

### Association between eating behaviour and dietary intake

In regression analyses, external eating was associated with a significantly higher energy intake in 15-year-old females, although no association with specific food groups and nutrients was observed. This suggests external eating promotes an energy-rich diet irrespective of specific foods. An association with sweets and soft drinks as found in a Swedish study on 12-year-old children (*n* = 1441) [18] was not confirmed in the present analysis.

Existing studies investigating emotional eating in children and adolescents predominantly reported higher intakes of sweets, soft drinks, and energy-rich food [18–20, 22]. Although similar non-significant trends in 10- and 15-year-old females were indeed observed in our study, we could not substantiate these findings when considering only significant associations after correcting for multiple testing. Surprisingly, we also found that in 10-, but not in 15-year-old females, a medium level of emotional eating was linked to a medium level of vegetable intake with the high level of vegetable intake showing the same, though non-significant, tendency. Possibly, parents whose children show signs of emotional eating try to counteract this by providing healthier foods. Despite no effects seen for high levels of emotional eating, it should be noted that the range of scores in the high tertile (score = 3–18) is quite broad, possibly limiting the power to detect a significant association.

A high level of dietary restraint was significantly associated with lower total energy intake in females and a similar, albeit non-significant, trend in males in the present analysis, which might reflect a deliberate low-calorie weight-loss strategy [14]. Furthermore, our results indicate an association of dietary restraint with higher intakes of food groups considered healthy (whole grains, fruit) in 15-year-old females, corroborating previous research [15, 17, 18]. Excluding vegetarians and vegans weakened the observed associations with fruit and whole grains in females, thus suggesting that they were mainly driven by this subgroup. Reduced energy intake or more selective food choices, as was observed with dietary restraint [17], may negatively impact nutrient intake. An example is the lower intake of omega-3 PUFA associated with medium dietary restraint. Nevertheless, it should also be considered that restrained eaters are more prone to underreporting [46, 47].

### Effect modification by BMI

Excluding participants with BMI < 10th or >90th percentile weakened some of the observed effects (e.g. with butter in males and omega-3 PUFA in females), suggesting that extreme body weight may present specific behaviour-diet relationships, and should be considered separate from the general population. Even after excluding these participants, numerous interactions with BMI were observed, indicating it is an important effect modifier. In males, associations of dietary restraint with high oil (at 15 years), and emotional eating with high dairy (at 10 years), occurred in opposite directions amongst the lowest (positive effect) and highest (inverse effect) BMI subgroups. In order to better understand eating behaviours in relation to diet, the interplay with BMI needs to be addressed in more depth in future studies.

### Strengths and limitations

The present study benefits from a large study population of males and females aged 10 and 15 years. Addressing both time-points is a key aspect given the behavioural changes occurring during pubertal development and their long-term impact on health [48] and eating behaviour [49]. Rather than selecting specific dietary outcomes, we included all major food groups in our analysis, thus allowing for a comprehensive understanding of the relationship with diet as a whole. Despite the elevated number of tests this implies, we observed significant associations following correction for multiple testing. Several possible shortcomings of the study must be considered. Even though the sampling was primarily population-based, due to non-random loss-to-follow-up, the cohort overrepresents children from higher socioeconomic background, with a lower prevalence of overweight and obesity, which might limit generalisability. Moreover, our findings are observational and based on cross-sectional analyses, and thus cannot infer causality and reverse causation is conceivable. Categorisation of exposure and outcome variables, although considered necessary given the skewed distribution, implies certain loss of information and may reduce the power to detect associations particularly in the higher tertiles. The questionnaire applied to assess eating behaviour is not validated to our knowledge, but is commonly used in epidemiologic studies. Due to the lack of assessment of dietary restraint at the 10-year follow-up, no conclusions can be drawn about differences in this eating behaviour among children and adolescents. Unfortunately, only three subscales of the EWI-C were assessed in the GINIplus and LISA studies, as the full inventory is extensive. As the study assessments involve numerous comprehensive questionnaires and examinations relevant to common chronic diseases, we strive to keep the burden on participants to a minimum in order to avoid drop-out. Ideally, all ten subscales would have been analysed in this study, and since we observed interactions with BMI in our analysis, those explicitly referring to the attitude to (over-)weight and body figure would be relevant for future research.

## Conclusion

The results of the present study indicate generally low reported levels of obesogenic eating behaviours among children and adolescents. Obesogenic eating behaviours present only few associations with diet, mainly with total energy intake and specific food groups among adolescent females. Numerous associations with diet were modified by BMI, indicating that future studies need to consider body weight even within a healthy weight population.

## Supplementary information

Supplemental Material

## Data Availability

The datasets generated and/or analysed during the current study are not publicly available due to data protection reasons, but are available on reasonable request, provided it is consistent with the consent given by the study participants. In some cases, ethical approval can be obtained for the release. Lastly, a data transfer agreement must be accepted and the request must be approved by the studies’ steering committees. Requests should be addressed to MS (marie.standl@helmholtz-muenchen.de).
