# Supplementary material for: Obesogenic eating behaviour and dietary intake in German children and adolescents: results from the GINIplus and LISA birth cohort studies
Source: Eur J Clin Nutr. 2022 Apr 1;76(10):1478–85. doi: 10.1038/s41430-022-01125-2 (PMC9550626; doi:10.1038/s41430-022-01125-2)
Supplement: Supplementary file 1 — Supplemental Material [file 41430_2022_1125_MOESM1_ESM.pdf]

Text S1. Assessment of dietary intake

Dietary intake at the 10- and 15-year follow-ups was assessed by a self-administered food frequency questionnaire (FFQ), designed and validated to measure 10-year-old children's usual food and nutrient intake over the past year [1]. The FFQ includes 80 food items and specific questions concerning preferred energy and fat content, preparation methods, and supplement use. The 80 FFQ food items were allocated into 41 groups and combined to form 17 major food groups, as described in Harris et al. [2]. Further details on the development of the FFQ, including food item selection, dietary vitamins, supplement use, and validation methods, have been previously described [1,3]. To estimate how often food was consumed over the previous year, subjects could choose one of nine frequency categories, including 'never', 'once a month', '2-3 times a month', 'once a week', '2-3 times a week', '4-6 times a week', 'once a day', '2-3 times a day' and 'four times a day or more'. In addition, common portion sizes were assigned for each food item to enable an estimation of quantities. For food items that are difficult to describe in common household measures, coloured photographs from the EPIC (European Prospective Investigation into Cancer and Nutrition) study showing three different portion sizes were provided [4]. A quality control procedure, based on recommendations by Willet et al. [5] for data cleaning in nutritional epidemiology, was applied at both follow-ups, which has been described in detail elsewhere [2]. In brief, participants were excluded if a complete block of food items was empty or more than 40 food items (50 % of the FFQ) were missing. To further reduce the risk of under- and over-reporting of food intake, participants were excluded, considering cut-offs defined by Willet et al., if daily total energy intake was outside 500-3,500 kcal (2,093-14,654 kJ) for females or 800-4,000 kcal (3,349-16,747 kJ) for males [5]. Moreover, exclusions were made if provided values for %EI of specific food items were implausible (outliers visually detected by means of boxplots). Consumption frequency and estimated portion sizes were converted into average daily intakes (g/d), and the corresponding energy and nutrient contents were calculated based on the German Food Code and Nutrient Database (BLS), version II.3.1, 2005 (Federal Research Center for Nutrition and Food (BfEL), Karlsruhe, Germany) [6]. Intakes relative to total daily energy intake were calculated as the ratio of energy from each food item or macronutrient to the total daily energy intake, and multiplied by 100 to obtain percentage contributions towards total energy intake (%EI). Due to the lack of energy content of water and tea, these food groups were presented in ml/day. Fibres are presented in g/day.

References (Text S1)

1 Stiegler P, Sausenthaler S, Buyken AE, Rzehak P, Czech D, Linseisen J et al. A new FFQ designed to measure the intake of fatty acids and antioxidants in children. Public Health Nutr. 2010;13:38–46.

2 Harris C, Flexeder C, Thiering E, Buyken A, Berdel D, Koletzko S et al. Changes in dietary intake during puberty and their determinants: results from the GINIplus birth cohort study. BMC Public Health. 2015;15:841.

3 Sausenthaler S, Standl M, Buyken A, Rzehak P, Koletzko S, Bauer CP et al. Regional and socio-economic differences in food, nutrient and supplement intake in school-age children in Germany: results from the GINIplus and the LISAplus studies. Public Health Nutr. 2011;14:1724–1735.

4 Bohlscheid-Thomas S, Hoting I, Boeing H, Wahrendorf J. Reproducibility and relative validity of food group intake in a food frequency questionnaire developed for the German part of the EPIC project. European Prospective Investigation into Cancer and Nutrition. Int J Epidemiol. 1997;26 Suppl 1:S59-70.

5 Willett W. Nutritional Epidemiology. Third Edition. Oxford University Press: Oxford, New York; 2012.

6 Hartmann BM, Bell S, Vásquez-Caicedo AL, Götz A, Brombach C. Der Bundeslebensmittelschlüssel. German Nutrient Data Base. Karlsruhe; 2005.

Table S1. Details on the assessment and categorisation of covariates

Covariate	Assessment	Categorisation
**Age [years]**	At 10-year and 15-year follow-ups: missing values for age at completing the FFQ were first replaced by age at completing the EWI-C, and second by age at completing the main questionnaires.	Continuous variable
**BMI [kg/m²]**	At 10-year and 15-year follow-ups: missing values for measured BMI at the examinations were replaced by reported BMI from the main questionnaires.	Continuous variable
**Puberty onset**	At 10-year follow-up: parents stated pubertal onset (acne or spots, pubic or axillary hair, breast development, menstruation, penis or testicle enlargement).	Yes; no
**Puberty stage**	At 15-year follow-up: Pubertal stage was assessed based on a self-rating pubertal development scale [7,8].	Pre-mid; late; post-pubertal
**Siblings**	At 10-year and 15-year follow-ups: presence of biological and not biological siblings.	Yes; no
**Moderate-vigorous physical activity**	At 10-year and 15-year follow-ups: Reported moderate-vigorous physical activity derived from a questionnaire was defined as weekly hours of exercise performed outside school, with exercise constituting any activity causing breathlessness or sweating.	Grouped based on age-specific percentiles as low: 25th percentile; medium: 25th–75th percentile; high: >75th percentile
**Screentime**	At 10-year and 15-year follow-ups: Number of hours the child spends in front of a screen (television, computer, gameboy, playstation) during a normal week per day (24 hours) in summer and winter.	Low: ≤2 h/day in summer and winter; high: >2 h/day in summer or winter
**Total difficulties**	At 10-year and 15-year follow-ups: Total difficulties were assessed by the SDQ and categorized on the basis of age-specific threshold values for 10-year-old [9,10] and 15-year-old children [11].	Normal; borderline; abnormal
**Parental education**	At birth, 3-year or 4-year follow-up: Parental education was defined by the highest grade completed by either the mother or the father on the basis of the German educational system.	Low-medium: ≤10th grade; high: >10th grade
**Parental BMI**	At 10-year and 15-year follow-ups: reported parental BMI was defined by the highest BMI category of either the mother or the father.	Normal: <25 kg/m²; overweight: ≥ 25 and <30 kg/m²; obese: ≥30 kg/m²
**Study arm**	In the GINI study, parents who's newborns had at least one first degree family member with an atopic disease were asked to participate in the intervention study arm, which investigated the effect of three different hydrolysed formulas on allergy development. All others were asked to participate in the observation study arm. There is no intervention arm in the LISA study.	GINI observation; GINI intervention; LISA
**Region**	GINIplus included the study areas Munich and Wesel, and LISA the study areas Munich, Leipzig, Bad Honnef and Wesel.	Munich; Leipzig; Bad Honnef; Wesel
**Total energy intake [kJ/day]**	At 10-year and 15-year follow-ups: total daily energy intake was assessed by a FFQ.	Continuous variable
**Total beverage intake [ml/day]**	At 10-year and 15-year follow-ups: total daily beverage intake was assessed by a FFQ.	Continuous variable

Abbreviations: BMI, body mass index; EWI-C, Eating Behaviour and Weight Problems Inventory for Children; FFQ, food frequency questionnaire; GINIplus, German Infant Nutritional Intervention plus environmental and genetic influences on allergy development; LISA, Influence of Lifestyle related factors on the development of the Immune System and Allergies in East and West Germany; SDQ, Strengths and Difficulties Questionnaire.

References (Table S1)

7 Carskadon MA, Acebo C. A self-administered rating scale for pubertal development. J Adolesc Health Off Publ Soc Adolesc Med. 1993;14:190–195.

8 Petersen AC, Crockett L, Richards M, Boxer A. A self-report measure of pubertal status: Reliability, validity, and initial norms. J Youth Adolesc. 1988;17:117–133.

9 Woerner W, Becker A, Friedrich C, Klasen H, Goodman R, Rothenberger A. [Normal values and evaluation of the German parents' version of Strengths and DIfficulties Questionnaire (SDQ): Results of a representative field study]. Z Kinder Jugendpsychiatr Psychother. 2002;30:105–112.

10 Woerner W, Becker A, Rothenberger A. Normative data and scale properties of the German parent SDQ. Eur Child Adolesc Psychiatry. 2004;13 Suppl 2:II3-10.

11 Goodman R. The Strengths and Difficulties Questionnaire: a research note. J Child Psychol Psychiatry. 1997;38:581–586.

Table S2. Description of food groups intake

	10-Year Follow-Up								
	Females (N=1,082)				Males (N=1,175)				
	Total	T1	T2	T3	Total	T1	T2	T3	P-value[a]
**Fruit**	4.2 (2.7; 6.1)	2.0 (1.3; 2.7)	4.2 (3.8; 4.8)	7.4 (6.1; 9.2)	3.3 (1.9; 5.0)	1.4 (0.9; 1.9)	3.3 (2.9; 3.7)	6.0 (5.1; 7.5)	**<0.001**
**Vegetable**	1.6 (1.0; 2.3)	0.8 (0.5; 1.0)	1.6 (1.4; 1.8)	2.8 (2.3; 3.5)	1.3 (0.8; 1.8)	0.6 (0.3; 0.8)	1.3 (1.1; 1.4)	2.2 (1.8; 2.7)	**<0.001**
**Starchy vegetables**	2.3 (1.4; 3.4)	1.2 (0.8; 1.4)	2.3 (1.9; 2.6)	4.1 (3.5; 5.1)	2.1 (1.4; 3.3)	1.1 (0.8; 1.4)	2.1 (1.9; 2.5)	4.0 (3.3; 4.8)	0.166
**Whole grains**	2.7 (0.7; 7.0)	0.3 (0; 0.7)	2.7 (1.8; 3.7)	9.3 (7.0; 12.5)	2.3 (0.4; 6.6)	0.1 (0; 0.4)	2.3 (1.6; 3.4)	9.2 (6.6; 13.2)	**0.048**
**Refined grains**	27 (21.8; 33.3)	19.4 (16.5; 21.8)	27.0 (25.5; 29.1)	36.4 (33.3; 40.4)	27.1 (21.1; 32.7)	18.9 (15.9; 21.1)	27.1 (25.3; 28.9)	35.9 (32.8; 40.9)	0.323
**Meat**	11.4 (8.1; 15.9)	6.7 (5.0; 8.1)	11.4 (10.4; 12.8)	18.5 (15.9; 21.7)	12.5 (9.2; 17.0)	7.7 (5.5; 9.2)	12.5 (11.5; 14.0)	19.1 (17.0; 22.6)	**<0.001**
**Fish**	1.2 (0.6; 1.9)	0.4 (0.1; 0.6)	1.2 (1.0; 1.4)	2.3 (1.9; 3.0)	1.2 (0.7; 1.9)	0.5 (0.2; 0.7)	1.2 (1.1; 1.4)	2.3 (1.9; 3.0)	0.223
**Eggs**	0.6 (0.3; 1.0)	0.2 (0.1; 0.3)	0.6 (0.5; 0.7)	1.2 (1.0; 1.7)	0.5 (0.3; 1.0)	0.2 (0.1; 0.3)	0.5 (0.5; 0.6)	1.2 (1.0; 1.6)	0.076
**Nuts and seeds**	0.3 (0.1; 0.8)	0 (0; 0.1)	0.3 (0.3; 0.5)	1.2 (0.8; 2.1)	0.3 (0.1; 0.8)	0 (0; 0.1)	0.3 (0.2; 0.4)	1.2 (0.8; 2.1)	0.293
**Butter**	0.7 (0.1; 2.6)	0 (0; 0.1)	0.7 (0.4; 1.2)	3.7 (2.6; 5.8)	0.8 (0.1; 2.6)	0 (0; 0.1)	0.8 (0.4; 1.2)	3.8 (2.6; 6.0)	0.971
**Margarine**	0.3 (0; 1.3)	0 (0; 0)	0.3 (0.2; 0.6)	2.1 (1.3; 3.6)	0.3 (0; 1.1)	0 (0; 0)	0.3 (0.1; 0.5)	1.7 (1.1; 2.9)	0.077
**Oils**	1.2 (0.5; 2.3)	0.4 (0.2; 0.5)	1.2 (1.0; 1.5)	3.0 (2.3; 4.1)	1.0 (0.5; 2.0)	0.3 (0.2; 0.5)	1.0 (0.8; 1.3)	2.7 (2.0; 4.0)	**0.003**
**Dairy**	16.1 (11.0; 22.4)	8.8 (6.4; 11.0)	16.1 (14.4; 18)	26.3 (22.5; 32.4)	17.0 (11.8; 23.4)	9.8 (7.1; 11.8)	17.0 (15.4; 18.9)	27.7 (23.5; 33.5)	**0.012**
**Sugar-sweetend foo**	9.8 (6.8; 14.5)	5.8 (4.4; 6.8)	9.8 (8.6; 11.2)	16.9 (14.5; 20.0)	10.0 (6.5; 14.3)	5.3 (4.1; 6.5)	10.0 (8.7; 11.3)	16.6 (14.3; 20.4)	0.473
**Caloric drinks**	7.4 (3.2; 13.2)	1.9 (1.0; 3.2)	7.4 (6.1; 9.1)	16.0 (13.2; 20.4)	8.3 (3.5; 14.1)	2.2 (1.1; 3.5)	8.3 (6.8; 10.0)	16.8 (14.1; 21.1)	**0.048**
**Tea [ml/d]**	23.6 (3.9; 103)	0 (0; 3.8)	23.7 (14.7; 38.1)	160 (105; 291)	14.4 (0; 78.0)	0 (0; 0)	14.4 (8.6; 22.3)	141 (78.7; 249)	**<0.001**
**Water [ml/d]**	600 (282; 918)	168 (58.8; 282)	601 (496; 697)	1,094 (920; 1,354)	610 (270; 973)	148 (27.1; 270)	612 (515; 721)	1,171 (975; 1,416)	0.482

	15-Year Follow-Up								
	Females (N=1,000)				Males (N=880)				
	Total	T1	T2	T3	Total	T1	T2	T3	P-value[a]
**Fruit**	3.9 (2.4; 6.5)	1.9 (1.1; 2.4)	3.9 (3.4; 4.6)	7.8 (6.5; 10.1)	2.3 (1.2; 3.8)	0.9 (0.5; 1.2)	2.3 (2.0; 2.7)	4.6 (3.9; 6.0)	**<0.001**
**Vegetable**	1.8 (1.1; 2.8)	0.8 (0.6; 1.1)	1.8 (1.6; 2.1)	3.4 (2.8; 4.5)	1.2 (0.7; 1.8)	0.5 (0.3; 0.7)	1.2 (1.0; 1.4)	2.3 (1.8; 3.1)	**<0.001**
**Starchy vegetables**	1.9 (1.2; 3.1)	1.0 (0.7; 1.2)	1.9 (1.7; 2.3)	3.7 (3.1; 4.9)	1.8 (1.2; 2.8)	1.0 (0.7; 1.2)	1.8 (1.6; 2.1)	3.5 (2.8; 4.2)	**0.073**
**Whole grains**	3.1 (1.1; 8.0)	0.5 (0; 1.1)	3.1 (2.2; 4.2)	10.2 (8.0; 13.9)	2.6 (0.7; 7.1)	0.3 (0; 0.7)	2.6 (1.9; 3.7)	9.9 (7.1; 14.3)	**0.025**
**Refined grains**	28.1 (22.0; 34.3)	20.1 (17.2; 22)	28.1 (26.0; 29.7)	37.0 (34.4; 41.4)	27.1 (21.1; 33.7)	19.3 (15.9; 21.1)	27.1 (25.0; 28.9)	37.1 (33.7; 41.3)	0.055
**Meat**	11.2 (7.1; 16.0)	5.4 (2.7; 7.1)	11.2 (10.0; 12.7)	18.4 (16.0; 22.6)	13.7 (9.8; 19.3)	8.2 (6.1; 9.8)	13.7 (12.5; 15.2)	21.6 (19.3; 25.0)	**<0.001**
**Fish**	1.0 (0.4; 1.8)	0.2 (0; 0.4)	1.0 (0.8; 1.2)	2.2 (1.8; 3.0)	1.2 (0.6; 1.9)	0.4 (0.1; 0.6)	1.2 (1.0; 1.4)	2.4 (1.9; 3.0)	**<0.001**
**Eggs**	0.6 (0.3; 1.1)	0.2 (0.1; 0.3)	0.6 (0.5; 0.7)	1.5 (1.1; 2.1)	0.6 (0.3; 1.0)	0.2 (0.1; 0.3)	0.6 (0.5; 0.7)	1.3 (1.0; 1.9)	0.156
**Nuts and seeds**	0.4 (0; 0.9)	0 (0; 0)	0.4 (0.3; 0.5)	1.4 (0.9; 2.4)	0.4 (0.1; 1.0)	0 (0; 0.1)	0.4 (0.3; 0.5)	1.4 (1.0; 2.4)	0.588
**Butter**	0.9 (0.1; 2.8)	0 (0; 0.1)	0.9 (0.5; 1.4)	3.9 (2.8; 5.8)	1.0 (0.2; 2.6)	0.1 (0; 0.2)	1.0 (0.6; 1.4)	3.5 (2.6; 5.2)	0.704
**Margarine**	0.2 (0; 1.0)	0 (0; 0)	0.2 (0.1; 0.4)	1.5 (1.0; 2.7)	0.1 (0; 0.7)	0 (0; 0)	0.1 (0; 0.3)	1.2 (0.7; 2.1)	**0.005**
**Oils**	1.4 (0.6; 2.5)	0.4 (0.2; 0.6)	1.4 (1.1; 1.7)	3.3 (2.5; 4.8)	1.2 (0.6; 2.2)	0.4 (0.2; 0.6)	1.2 (0.9; 1.5)	2.7 (2.2; 4.1)	**0.002**
**Dairy**	14.0 (9.0; 20.4)	7.6 (5.0; 9.0)	14.0 (12.5; 15.7)	24.2 (20.4; 29.4)	14.0 (9.0; 20.7)	7.2 (5.0; 9.0)	14.0 (12.0; 16.0)	24.5 (20.7; 30.4)	0.950
**Sugar-sweetend foo**	10.3 (6.6; 15.3)	5.5 (3.9; 6.6)	10.3 (8.9; 11.8)	18.5 (15.3; 23.1)	9.9 (6.5; 14.6)	5.3 (3.8; 6.5)	9.9 (8.9; 11.1)	17.1 (14.6; 22.4)	0.168
**Caloric drinks**	6.0 (2.3; 12.8)	1.2 (0.6; 2.3)	6.0 (4.6; 7.9)	16.5 (12.8; 23.2)	9.3 (4.5; 16.4)	3.0 (1.2; 4.5)	9.3 (7.8; 11.6)	19.6 (16.5; 24.3)	**<0.001**
**Tea [ml/d]**	30.8 (6.0; 147)	0 (0; 6.0)	31.0 (19.4; 53.8)	209 (148; 335)	15.0 (0; 83.0)	0 (0; 0)	15.1 (8.5; 26.3)	159 (82.8; 309)	**<0.001**
**Water [ml/d]**	926 (552; 1,366)	356 (121; 552)	928 (800; 1,053)	1,606 (1,366; 1,890)	993 (476; 1,551)	265 (61.0; 476)	994 (866; 1,186)	1,791 (1,556; 2,183)	0.052

Abbreviations: T1, tertile 1; T2, tertile; T3, tertile 3. Values are presented as medians (25th; 75th percentile) for continuous variables in %EI unless stated otherwise. Comparison between males (total) and females (total): tested by Wilcoxon's rank sum test for continuous variables. Significant differences are marked in bold: $p < 0.05$.

Table S3. Description of total energy and nutrients intake

	10-Year Follow-Up			15-Year Follow-Up		
	**Females (N=1,082)**	**Males (N=1,175)**	**P-value[a]**	**Females (N=1,000)**	**Males (N=880)**	**P-value[a]**
**Total energy [kJ/d]**	7,401 (6,107; 8,819)	8,633 (7,082; 10,254)	**<0.001**	7,204 (5,723; 9,008)	9,721 (7,796; 11,709)	**<0.001**
**Fat**	29.2 (25.6; 33.1)	29.6 (26.3; 33.5)	0.097	29.5 (26.0; 33.5)	30.4 (27.0; 34.3)	**0.002**
**Protein**	14.5 (13.1; 16.2)	14.8 (13.3; 16.4)	0.068	14.8 (12.9; 16.5)	15.2 (13.5; 17.1)	**<0.001**
**Carbohydrate**	55.1 (50.8; 59.4)	54.7 (50.3; 58.9)	0.058	55.0 (49.9; 59.3)	53.4 (48.9; 57.9)	**<0.001**
**Fibres [g/d]**	17.0 (13.7; 21.2)	18.0 (14.1; 22.5)	**<0.001**	17.0 (13.3; 22.1)	18.6 (14.2; 23.3)	**<0.001**
**Total sugar**	25.6 (20.7; 30.6)	25.6 (20.8; 30.8)	0.576	24.4 (19.2; 30.3)	24.7 (19.2; 30.0)	0.646
**SFA**	12.2 (10.4; 14.1)	12.4 (10.8; 14.4)	**0.017**	12.3 (10.3; 14.3)	12.6 (10.7; 14.6)	0.028
**MUFA**	10.4 (8.9; 11.9)	10.4 (9.2; 12.0)	0.059	10.3 (8.9; 12.0)	10.9 (9.3; 12.4)	**<0.001**
**PUFA**	4.2 (3.7; 5.0)	4.2 (3.6; 4.9)	0.215	4.4 (3.8; 5.2)	4.4 (3.8; 5.2)	0.973
**omega-3 PUFA**	0.5 (0.5; 0.6)	0.5 (0.5; 0.6)	**0.007**	0.6 (0.5; 0.6)	0.6 (0.5; 0.6)	0.090
**omega-6 PUFA**	3.7 (3.2; 4.3)	3.6 (3.1; 4.3)	0.304	3.8 (3.2; 4.6)	3.8 (3.2; 4.5)	0.829

Abbreviations: MUFA, monounsaturated fatty acids. PUFA, polyunsaturated fatty acids. SFA: saturated fatty acids. Values are presented as medians (25th; 75th percentile) for continuous variables in %EI unless stated otherwise. [a]Comparison between males (total) and females (total): tested by Wilcoxon's rank sum test for continuous variables. Significant differences are marked in bold: $p < 0.05$.

Table S4. Association between tertiles of eating behaviours and dietary intake in females at the 10-year follow-up

		External eating (N=1,082)				Emotional eating (N=1,082)			
		T2 (n=396) Score=5-8		T3 (n=305) Score=9-24		T2 (n=300) Score=1-2		T3 (n=321) Score=3-18	
		Estimate (95% CI)	P-value	Estimate (95% CI)	P-value	Estimate (95% CI)	P-value	Estimate (95% CI)	P-value
Fruit[a]	T2	0.90 (0.63;1.30)	0.5750	0.82 (0.56;1.22)	0.3293	1.28 (0.88;1.86)	0.1961	0.92 (0.64;1.32)	0.6530
	T3	1.01 (0.69;1.47)	0.9648	0.76 (0.51;1.15)	0.1998	1.49 (1.01;2.20)	0.0438	0.99 (0.68;1.45)	0.9706
Vegetable[a]	T2	1.16 (0.81;1.67)	0.4225	0.99 (0.67;1.47)	0.9777	1.84 (1.26;2.69)	**0.0016**	0.81 (0.56;1.17)	0.2632
	T3	0.96 (0.66;1.38)	0.8118	0.66 (0.44;1.00)	0.0476	1.71 (1.15;2.53)	0.0077	0.84 (0.58;1.22)	0.3544
Starchy	T2	1.08 (0.76;1.54)	0.6738	1.06 (0.71;1.59)	0.7632	0.88 (0.61;1.27)	0.4961	1.05 (0.72;1.52)	0.8066
vegetables[a]	T3	0.90 (0.61;1.32)	0.5838	1.35 (0.89;2.04)	0.1585	0.93 (0.63;1.38)	0.7145	1.32 (0.89;1.94)	0.1658
Whole grains[a]	T2	0.92 (0.64;1.31)	0.6245	0.58 (0.39;0.85)	0.0056	0.85 (0.60;1.22)	0.3904	0.73 (0.50;1.05)	0.0889
	T3	1.11 (0.77;1.59)	0.5827	0.73 (0.49;1.07)	0.1093	0.80 (0.55;1.16)	0.2361	0.97 (0.67;1.39)	0.8557
Refined grains[a]	T2	0.84 (0.59;1.19)	0.3214	0.91 (0.62;1.34)	0.6319	1.07 (0.74;1.54)	0.7260	0.93 (0.65;1.33)	0.6874
	T3	1.09 (0.76;1.57)	0.6402	1.26 (0.85;1.87)	0.2479	1.16 (0.80;1.68)	0.4276	0.94 (0.65;1.36)	0.7370
Meat[a]	T2	1.23 (0.86;1.75)	0.2519	1.22 (0.83;1.80)	0.3165	0.77 (0.53;1.12)	0.1664	1.27 (0.89;1.82)	0.1882
	T3	1.07 (0.75;1.53)	0.7117	1.16 (0.79;1.71)	0.4568	1.07 (0.75;1.53)	0.7004	0.96 (0.66;1.40)	0.8481
Fish[a]	T2	0.91 (0.64;1.30)	0.6122	1.16 (0.79;1.72)	0.4479	1.46 (1.02;2.11)	0.0411	1.02 (0.71;1.46)	0.9312
	T3	0.83 (0.58;1.18)	0.2911	1.11 (0.75;1.64)	0.6165	1.26 (0.87;1.82)	0.2290	1.00 (0.70;1.43)	1.0000
Eggs[a]	T2	1.12 (0.79;1.60)	0.5178	1.19 (0.82;1.75)	0.3611	1.35 (0.93;1.95)	0.1127	1.21 (0.85;1.72)	0.2965
	T3	1.21 (0.85;1.72)	0.2911	1.10 (0.74;1.62)	0.6332	1.71 (1.19;2.47)	0.0041	1.18 (0.82;1.70)	0.3675
Nuts and seeds[a]	T2	0.76 (0.53;1.08)	0.1196	0.76 (0.52;1.11)	0.1571	0.86 (0.60;1.24)	0.4267	0.86 (0.60;1.23)	0.4065
	T3	1.08 (0.76;1.55)	0.6573	0.97 (0.66;1.43)	0.8763	0.98 (0.68;1.40)	0.8992	0.96 (0.67;1.38)	0.8384
Butter[a]	T2	0.97 (0.66;1.41)	0.8681	1.03 (0.68;1.57)	0.8915	1.27 (0.86;1.88)	0.2267	1.22 (0.82;1.80)	0.3224
	T3	0.84 (0.56;1.26)	0.4040	0.87 (0.56;1.35)	0.5280	0.95 (0.63;1.44)	0.8085	0.94 (0.62;1.42)	0.7707
Margarine[a]	T2	1.42 (0.99;2.06)	0.0601	1.08 (0.72;1.61)	0.7260	0.87 (0.60;1.28)	0.4904	1.18 (0.81;1.71)	0.3914
	T3	1.02 (0.69;1.51)	0.9232	1.21 (0.80;1.84)	0.3658	1.24 (0.83;1.85)	0.2872	1.36 (0.91;2.03)	0.1312
Oils[a]	T2	1.16 (0.82;1.66)	0.4033	1.07 (0.72;1.58)	0.7487	1.23 (0.85;1.79)	0.2806	1.14 (0.79;1.63)	0.4824
	T3	0.95 (0.66;1.37)	0.7990	0.94 (0.63;1.39)	0.7460	1.77 (1.21;2.57)	0.0031	1.23 (0.85;1.80)	0.2738
Dairy[a]	T2	0.77 (0.54;1.10)	0.1442	1.06 (0.72;1.55)	0.7801	0.98 (0.68;1.42)	0.9294	1.15 (0.80;1.64)	0.4530
	T3	0.80 (0.56;1.14)	0.2133	0.78 (0.52;1.17)	0.2262	0.76 (0.53;1.10)	0.1521	0.72 (0.50;1.05)	0.0846
Sugar-sweetend	T2	1.47 (1.03;2.10)	0.0327	1.62 (1.10;2.39)	0.0136	1.42 (1.00;2.03)	0.0504	1.35 (0.93;1.95)	0.1156
food[a]	T3	1.50 (1.05;2.13)	0.0242	1.41 (0.95;2.08)	0.0852	0.95 (0.66;1.38)	0.7878	1.61 (1.13;2.31)	0.0090
Caloric drinks[a]	T2	1.22 (0.86;1.74)	0.2729	1.26 (0.85;1.87)	0.2432	1.17 (0.81;1.68)	0.4032	0.96 (0.67;1.38)	0.8377
	T3	0.85 (0.59;1.21)	0.3552	0.98 (0.66;1.44)	0.9037	1.00 (0.69;1.44)	0.9931	0.94 (0.66;1.35)	0.7440
Water[a] [ml/d]	T2	1.27 (0.88;1.83)	0.1964	1.05 (0.71;1.55)	0.8173	0.86 (0.60;1.25)	0.4274	1.05 (0.72;1.52)	0.8105
	T3	1.18 (0.80;1.74)	0.4048	0.79 (0.51;1.21)	0.2744	0.85 (0.57;1.26)	0.4058	0.93 (0.62;1.39)	0.7298
Tea[a] [ml/d]	T2	1.37 (0.96;1.96)	0.0817	1.15 (0.78;1.70)	0.4903	0.83 (0.58;1.19)	0.3156	1.09 (0.76;1.58)	0.6341
	T3	0.99 (0.68;1.44)	0.9466	1.00 (0.67;1.50)	0.9995	0.80 (0.54;1.17)	0.2508	0.93 (0.63;1.37)	0.7107
Total energy[b] [kJ/d]		215 (-74;503)	0.1445	392 (78;707)	0.0146	24 (-273;321)	0.8741	186 (-109;480)	0.2167
Fat[b]		-0.09 (-0.87;0.69)	0.8285	0.06 (-0.80;0.91)	0.8962	0.12 (-0.68;0.93)	0.7600	-0.19 (-0.99;0.60)	0.6379
Protein[b]		0.00 (-0.35;0)	0.9824	0.06 (-0.31;0.44)	0.7486	0.00 (-0.35;0.36)	0.9854	-0.24 (-0.59;0.11)	0.1813
Carbohydrate[b]		0.10 (-0.83;1.03)	0.8285	-0.11 (-1.13;0.90)	0.8279	-0.15 (-1.10;0.81)	0.7604	0.44 (-0.51;1.38)	0.3645
Fibres[b] [g/d]		-0.05 (-0.71;0.62)	0.8868	-0.71 (-1.43;0.02)	0.0557	0.37 (-0.32;1.05)	0.2912	-0.29 (-0.97;0.39)	0.4027
Total sugar[b]		-0.20 (-1.22;0.82)	0.6979	-0.23 (-1.35;0.88)	0.6808	-0.02 (-1.06;1.03)	0.9748	0.52 (-0.52;1.56)	0.3238
SFA[b]		-0.04 (-0.43;0.34)	0.8288	-0.04 (-0.46;0.39)	0.8648	-0.20 (-0.59;0.20)	0.3309	-0.17 (-0.56;0.23)	0.4045
MUFA[b]		0.01 (-0.32;0.34)	0.9439	0.10 (-0.26;0.46)	0.5954	0.09 (-0.26;0.43)	0.6205	-0.08 (-0.42;0.26)	0.6329
PUFA[c]		0.99 (0.96;1.03)	0.6490	1.00 (0.97;1.04)	0.8544	1.04 (1.00;1.08)	0.0309	1.02 (0.98;1.05)	0.3156
omega-3 PUFA[c]		0.99 (0.96;1.02)	0.4946	1.01 (0.98;1.04)	0.4318	1.04 (1.01;1.07)	0.0111	1.01 (0.98;1.04)	0.4448
omega-6 PUFA[c]		0.99 (0.96;1.03)	0.6985	1.00 (0.96;1.04)	0.9131	1.04 (1.00;1.08)	0.0463	1.02 (0.98;1.06)	0.3193

Abbreviations: CI, confidence interval; MUFA, monounsaturated fatty acids; PUFA, polyunsaturated fatty acids; SFA, saturated fatty acids; T2, tertile 2; T3, tertile 3. [a]Effect estimates of multinomial logistic regression are presented as relative risk ratio (95% CI). [b]Effect estimates of multiple linear regression are presented as beta coefficient (95% CI). [c]Effect estimates of multiple linear regression for naturally log-transformed outcome variables are presented as means ratio (95% CI). All models were adjusted for age, BMI, pubertal status, siblings, moderate-to-vigorous physical activity, screen time, total difficulties, parental education, parental BMI, study, and recruitment region. Food groups (except water and tea) and nutrients models were further adjusted for total daily energy intake. Water and Tea models were further adjusted for total daily beverage intake. Tertile 1 is the reference category. Significant associations are marked in bold: $p<0.0019$.

Table S5. Association between tertiles of eating behaviours and dietary intake in males at the 10-year follow-up

		**External eating (N=1,175)**				**Emotional eating (N=1,175)**			
		**T2 (n=376) Score=6-9**		**T3 (n=314) Score=10-24**		**T2 (n=335) Score=1-2**		**T3 (n=354) Score=3-24**	
		**Estimate (95% CI)**	**P-value**	**Estimate (95% CI)**	**P-value**	**Estimate (95% CI)**	**P-value**	**Estimate (95% CI)**	**P-value**
**Fruit[a]**	**T2**	0.82 (0.58;1.15)	0.2430	0.94 (0.65;1.35)	0.7277	0.96 (0.68;1.36)	0.8100	1.04 (0.73;1.48)	0.8411
	**T3**	0.92 (0.65;1.31)	0.6603	0.95 (0.65;1.38)	0.7706	1.04 (0.72;1.50)	0.8445	1.31 (0.91;1.90)	0.1450
**Vegetable[a]**	**T2**	1.30 (0.93;1.82)	0.1317	1.03 (0.71;1.48)	0.8897	0.86 (0.61;1.22)	0.4032	1.08 (0.76;1.53)	0.6628
	**T3**	0.97 (0.68;1.38)	0.8737	1.03 (0.71;1.49)	0.8769	1.14 (0.80;1.63)	0.4729	1.17 (0.81;1.69)	0.3937
**Starchy**	**T2**	0.86 (0.61;1.22)	0.3975	1.09 (0.76;1.57)	0.6291	0.82 (0.57;1.18)	0.2827	0.98 (0.69;1.39)	0.9081
**vegetables[a]**	**T3**	0.90 (0.62;1.31)	0.5838	1.13 (0.76;1.68)	0.5606	1.05 (0.72;1.54)	0.7930	1.05 (0.71;1.54)	0.8205
**Whole grains[a]**	**T2**	1.21 (0.86;1.71)	0.2758	1.35 (0.94;1.94)	0.1093	1.05 (0.73;1.49)	0.7993	1.28 (0.90;1.82)	0.1737
	**T3**	1.03 (0.73;1.45)	0.8809	0.87 (0.59;1.27)	0.4646	1.13 (0.79;1.62)	0.5024	1.12 (0.78;1.62)	0.5376
**Refined grains[a]**	**T2**	1.13 (0.81;1.59)	0.4633	1.25 (0.87;1.79)	0.2320	0.99 (0.69;1.41)	0.9423	1.28 (0.90;1.80)	0.1684
	**T3**	0.96 (0.68;1.35)	0.8188	1.00 (0.69;1.44)	0.9922	0.93 (0.66;1.32)	0.6858	0.81 (0.56;1.16)	0.2514
**Meat[a]**	**T2**	1.03 (0.73;1.44)	0.8842	0.92 (0.64;1.32)	0.6357	0.98 (0.69;1.38)	0.9089	0.91 (0.64;1.29)	0.5891
	**T3**	1.33 (0.95;1.87)	0.1009	1.26 (0.88;1.80)	0.2113	1.09 (0.77;1.55)	0.6316	1.22 (0.86;1.73)	0.2652
**Fish[a]**	**T2**	1.04 (0.74;1.45)	0.8383	1.22 (0.84;1.75)	0.2932	0.80 (0.56;1.13)	0.2071	1.01 (0.71;1.44)	0.9348
	**T3**	0.99 (0.70;1.39)	0.9519	1.37 (0.95;1.97)	0.0908	0.87 (0.61;1.24)	0.4393	1.06 (0.74;1.51)	0.7531
**Eggs[a]**	**T2**	0.92 (0.65;1.29)	0.6143	0.95 (0.66;1.37)	0.7991	1.26 (0.88;1.80)	0.2060	0.83 (0.59;1.18)	0.3057
	**T3**	0.95 (0.68;1.33)	0.7699	0.90 (0.62;1.29)	0.5609	1.60 (1.12;2.29)	0.0096	1.00 (0.70;1.42)	0.9882
**Nuts and seeds[a]**	**T2**	1.12 (0.80;1.57)	0.4930	1.25 (0.87;1.79)	0.2329	1.14 (0.81;1.61)	0.4594	0.89 (0.63;1.27)	0.5255
	**T3**	0.93 (0.66;1.30)	0.6762	1.07 (0.74;1.53)	0.7298	1.22 (0.86;1.74)	0.2701	1.17 (0.83;1.66)	0.3644
**Butter[a]**	**T2**	1.13 (0.78;1.62)	0.5177	1.18 (0.79;1.75)	0.4134	1.52 (1.04;2.23)	0.0312	1.09 (0.75;1.60)	0.6494
	**T3**	1.15 (0.79;1.68)	0.4686	1.31 (0.87;1.97)	0.1977	1.42 (0.96;2.11)	0.0821	0.97 (0.65;1.44)	0.8608
**Margarine[a]**	**T2**	0.74 (0.51;1.05)	0.0944	1.06 (0.73;1.52)	0.7695	0.85 (0.59;1.22)	0.3734	1.14 (0.80;1.63)	0.4787
	**T3**	0.91 (0.63;1.31)	0.5966	0.68 (0.46;1.03)	0.0662	1.04 (0.71;1.53)	0.8274	1.05 (0.72;1.55)	0.7902
**Oils[a]**	**T2**	0.99 (0.70;1.39)	0.9317	0.97 (0.67;1.41)	0.8769	0.85 (0.60;1.22)	0.3809	1.22 (0.85;1.75)	0.2743
	**T3**	1.31 (0.92;1.87)	0.1400	1.48 (1.01;2.16)	0.0436	0.98 (0.68;1.41)	0.9015	1.28 (0.88;1.86)	0.1935
**Dairy[a]**	**T2**	0.74 (0.53;1.04)	0.0848	0.66 (0.46;0.95)	0.0270	0.74 (0.52;1.05)	0.0943	0.87 (0.61;1.23)	0.4195
	**T3**	0.79 (0.55;1.12)	0.1838	0.70 (0.48;1.02)	0.0670	0.85 (0.59;1.21)	0.3664	0.90 (0.62;1.30)	0.5791
**Sugar-sweetend**	**T2**	1.25 (0.90;1.75)	0.1850	1.03 (0.71;1.49)	0.8664	1.20 (0.84;1.70)	0.3099	1.05 (0.74;1.49)	0.7982
**food[a]**	**T3**	1.01 (0.71;1.43)	0.9592	1.37 (0.95;1.97)	0.0876	1.26 (0.88;1.79)	0.2073	1.12 (0.79;1.60)	0.5200
**Caloric drinks[a]**	**T2**	0.84 (0.60;1.18)	0.3254	0.93 (0.65;1.33)	0.6894	0.88 (0.62;1.24)	0.4592	0.74 (0.52;1.05)	0.0896
	**T3**	1.07 (0.76;1.50)	0.6933	0.99 (0.69;1.43)	0.9582	1.01 (0.71;1.43)	0.9775	0.74 (0.52;1.05)	0.0946
**Water[a] [ml/d]**	**T2**	1.34 (0.95;1.90)	0.0958	1.34 (0.93;1.95)	0.1202	0.89 (0.63;1.27)	0.5318	0.81 (0.56;1.17)	0.2653
	**T3**	1.01 (0.70;1.47)	0.9441	1.14 (0.77;1.69)	0.5219	0.85 (0.58;1.25)	0.4152	1.20 (0.82;1.75)	0.3562
**Tea[a] [ml/d]**	**T2**	1.02 (0.72;1.44)	0.9296	1.17 (0.80;1.71)	0.4154	1.01 (0.70;1.46)	0.9554	1.05 (0.73;1.51)	0.7876
	**T3**	0.93 (0.65;1.34)	0.7080	1.18 (0.79;1.75)	0.4134	1.29 (0.89;1.89)	0.1780	1.08 (0.74;1.59)	0.6917
**Total energy[b] [kJ/d]**		239 (-91;570)	0.1551	257 (-95;609)	0.1521	453 (114;793)	0.0089	434 (93;774)	0.0126
**Fat[b]**		0.67 (-0.09;1.42)	0.0837	0.91 (0.10;1.72)	0.0270	0.37 (-0.42;1.15)	0.3596	0.71 (-0.07;1.50)	0.0751
**Protein[b]**		-0.05 (-0.38;0.28)	0.7555	-0.15 (-0.50;0.20)	0.4055	0.00 (-0.34;0.35)	0.9857	0.23 (-0.12;0.57)	0.1974
**Carbohydrate[b]**		-0.61 (-1.51;0.30)	0.1875	-0.75 (-1.71;0.21)	0.1268	-0.37 (-1.30;0.56)	0.4349	-0.94 (-1.87;0.00)	0.0494
**Fibres[b] [g/d]**		0.01 (-0.70;0.73)	0.9698	0.01 (-0.74;0.77)	0.9711	0.39 (-0.35;1.12)	0.3018	0.42 (-0.32;1.16)	0.2642
**Total sugar[b]**		-0.24 (-1.22;0.74)	0.6307	-0.55 (-1.59;0.50)	0.3049	-0.14 (-1.16;0.87)	0.7811	-0.65 (-1.66;0.37)	0.2125
**SFA[b]**		0.25 (-0.13;0.62)	0.1931	0.34 (-0.05;0.74)	0.0910	0.09 (-0.30;0.48)	0.6493	0.19 (-0.20;0.58)	0.3436
**MUFA[b]**		0.31 (-0.01;0.63)	0.0596	0.36 (0.02;0.70)	0.0368	0.16 (-0.17;0.49)	0.3355	0.29 (-0.04;0.62)	0.0833
**PUFA[c]**		1.02 (0.99;1.06)	0.2390	1.03 (0.99;1.07)	0.1114	1.02 (0.99;1.06)	0.2449	1.04 (1.00;1.08)	0.0374
**omega-3 PUFA[c]**		1.00 (0.97;1.03)	0.8564	1.03 (1.00;1.06)	0.0791	1.00 (0.97;1.03)	0.9117	1.04 (1.00;1.07)	0.0227
**omega-6 PUFA[c]**		1.02 (0.99;1.06)	0.1962	1.03 (0.99;1.07)	0.1393	1.02 (0.99;1.06)	0.2150	1.04 (1.00;1.08)	0.0523

Abbreviations: CI, confidence interval; MUFA, monounsaturated fatty acids; PUFA, polyunsaturated fatty acids; SFA, saturated fatty acids; T2, tertile 2; T3, tertile 3. [a]Effect estimates of multinomial logistic regression are presented as relative risk ratio (95% CI). [b]Effect estimates of multiple linear regression are presented as beta coefficient (95% CI). [c]Effect estimates of multiple linear regression for naturally log-transformed outcome variables are presented as means ratio (95% CI). All models were adjusted for age, BMI, pubertal status, siblings, moderate-to-vigorous physical activity, screen time, total difficulties, parental education, parental BMI, study, and recruitment region. Food groups (except water and tea) and nutrients models were further adjusted for total daily energy intake. Water and Tea models were further adjusted for total daily beverage intake. Tertile 1 is the reference category. Significant associations are marked in bold: $p<0.0019$.

Table S6. Association between tertiles of eating behaviours and dietary intake in females at the 15-year follow-up

		External eating (N=1,000)				Emotional eating (N=1,000)				Dietary restraint (N=1,000)			
		T2 (n=315) Score=6-9		T3 (n=313) Score=10-22		T2 (n=236) Score=3-5		T3 (n=305) Score=6-24		T2 (n=277) Score=3-6		T3 (n=303) Score=7-21	
		Estimate (95% CI)	P-value	Estimate (95% CI)	P-value	Estimate (95% CI)	P-value	Estimate (95% CI)	P-value	Estimate (95% CI)	P-value	Estimate (95% CI)	P-value
**Fruit[a]**	**T2**	1.04 (0.71;1.53)	0.8318	1.11 (0.75;1.64)	0.6040	0.81 (0.54;1.21)	0.2973	0.96 (0.65;1.40)	0.8156	1.00 (0.68;1.48)	0.9968	1.33 (0.89;2.01)	0.1643
	**T3**	1.04 (0.69;1.57)	0.8459	1.13 (0.74;1.71)	0.5789	0.79 (0.52;1.22)	0.2867	1.14 (0.76;1.72)	0.5256	1.52 (1.00;2.32)	0.0494	2.20 (1.43;3.39)	**0.0003**
**Vegetable[a]**	**T2**	1.16 (0.79;1.70)	0.4617	1.19 (0.80;1.75)	0.3873	0.88 (0.59;1.31)	0.5229	1.14 (0.78;1.67)	0.5099	1.32 (0.90;1.95)	0.1609	1.11 (0.74;1.67)	0.6079
	**T3**	1.01 (0.67;1.52)	0.9613	1.05 (0.70;1.59)	0.8108	0.63 (0.41;0.97)	0.0343	1.25 (0.84;1.88)	0.2738	1.28 (0.84;1.94)	0.2558	1.59 (1.05;2.42)	0.0292
**Starchy**	**T2**	1.29 (0.87;1.90)	0.2005	1.56 (1.05;2.30)	0.0259	1.21 (0.82;1.80)	0.3405	1.07 (0.73;1.56)	0.7250	0.72 (0.49;1.06)	0.0982	0.76 (0.51;1.14)	0.1869
**vegetables[a]**	**T3**	1.38 (0.92;2.09)	0.1202	1.60 (1.06;2.43)	0.0268	1.05 (0.68;1.61)	0.8402	1.24 (0.83;1.85)	0.2922	0.64 (0.42;0.98)	0.0400	0.69 (0.45;1.06)	0.0912
**Whole grains[a]**	**T2**	1.05 (0.72;1.52)	0.8049	1.26 (0.85;1.85)	0.2500	1.03 (0.70;1.53)	0.8707	1.34 (0.92;1.95)	0.1248	1.74 (1.18;2.58)	0.0054	1.32 (0.89;1.97)	0.1635
	**T3**	0.89 (0.60;1.31)	0.5472	1.25 (0.84;1.84)	0.2739	1.00 (0.67;1.49)	0.9866	1.39 (0.95;2.05)	0.0924	1.94 (1.30;2.91)	**0.0013**	1.82 (1.22;2.72)	0.0033
**Refined grains[a]**	**T2**	1.21 (0.83;1.76)	0.3255	1.15 (0.78;1.70)	0.4811	0.87 (0.58;1.30)	0.5024	0.83 (0.58;1.21)	0.3382	0.85 (0.57;1.27)	0.4301	0.59 (0.40;0.88)	0.0087
	**T3**	1.14 (0.78;1.68)	0.5005	1.64 (1.12;2.42)	0.0117	1.10 (0.74;1.64)	0.6256	0.85 (0.58;1.24)	0.4091	1.16 (0.78;1.72)	0.4645	0.57 (0.38;0.85)	0.0058
**Meat[a]**	**T2**	0.95 (0.65;1.38)	0.7748	0.98 (0.67;1.43)	0.9149	1.43 (0.96;2.12)	0.0776	0.96 (0.66;1.40)	0.8325	0.75 (0.51;1.12)	0.1575	0.82 (0.56;1.22)	0.3326
	**T3**	1.09 (0.74;1.61)	0.6467	1.07 (0.73;1.59)	0.7198	0.98 (0.65;1.47)	0.9118	0.78 (0.54;1.15)	0.2108	0.99 (0.67;1.46)	0.9445	0.73 (0.48;1.09)	0.1245
**Fish[a]**	**T2**	0.65 (0.44;0.95)	0.0272	0.99 (0.68;1.46)	0.9696	1.05 (0.71;1.56)	0.8102	0.85 (0.59;1.24)	0.4069	1.05 (0.72;1.54)	0.8067	0.92 (0.62;1.36)	0.6705
	**T3**	0.92 (0.63;1.34)	0.6577	1.05 (0.71;1.55)	0.8170	1.04 (0.70;1.54)	0.8542	0.79 (0.54;1.15)	0.2240	0.93 (0.63;1.38)	0.7335	1.08 (0.74;1.59)	0.6889
**Eggs[a]**	**T2**	1.03 (0.71;1.51)	0.8603	1.26 (0.87;1.84)	0.2260	1.29 (0.86;1.93)	0.2140	1.41 (0.97;2.03)	0.0692	0.99 (0.68;1.44)	0.9405	0.83 (0.56;1.24)	0.3609
	**T3**	1.16 (0.80;1.69)	0.4389	1.00 (0.68;1.48)	0.9850	1.51 (1.02;2.23)	0.0414	1.14 (0.78;1.66)	0.4976	0.78 (0.53;1.16)	0.2177	1.16 (0.79;1.71)	0.4457
**Nuts and seeds[a]**	**T2**	1.00 (0.69;1.46)	0.9926	0.94 (0.64;1.37)	0.7325	1.10 (0.74;1.62)	0.6459	0.86 (0.59;1.25)	0.4260	1.03 (0.70;1.52)	0.8850	0.95 (0.64;1.39)	0.7782
	**T3**	0.98 (0.67;1.44)	0.9207	1.01 (0.69;1.48)	0.9529	0.87 (0.58;1.30)	0.4918	0.79 (0.54;1.14)	0.2066	1.29 (0.87;1.90)	0.1988	1.06 (0.72;1.57)	0.7693
**Butter[a]**	**T2**	1.32 (0.89;1.97)	0.1733	1.13 (0.75;1.69)	0.5637	1.05 (0.69;1.58)	0.8297	0.85 (0.57;1.27)	0.4300	1.16 (0.77;1.75)	0.4828	0.94 (0.62;1.41)	0.7626
	**T3**	1.53 (1.02;2.30)	0.0382	1.47 (0.97;2.20)	0.0668	0.86 (0.56;1.32)	0.4865	1.00 (0.67;1.48)	0.9983	0.91 (0.60;1.37)	0.6458	0.55 (0.36;0.84)	0.0055
**Margarine[a]**	**T2**	1.23 (0.83;1.84)	0.3057	1.30 (0.88;1.93)	0.1853	1.10 (0.73;1.65)	0.6433	1.00 (0.68;1.46)	0.9858	0.68 (0.45;1.01)	0.0562	0.61 (0.40;0.91)	0.0162
	**T3**	1.21 (0.81;1.83)	0.3540	1.07 (0.70;1.62)	0.7568	1.01 (0.66;1.55)	0.9580	0.98 (0.66;1.47)	0.9259	0.69 (0.45;1.05)	0.0838	0.64 (0.42;0.99)	0.0426
**Oils[a]**	**T2**	0.94 (0.64;1.37)	0.7387	1.13 (0.77;1.66)	0.5342	0.81 (0.54;1.22)	0.3169	1.06 (0.74;1.54)	0.7467	1.01 (0.68;1.49)	0.9613	0.87 (0.59;1.29)	0.4841
	**T3**	0.96 (0.65;1.41)	0.8176	0.92 (0.62;1.36)	0.6671	1.16 (0.78;1.74)	0.4543	1.02 (0.69;1.51)	0.9264	1.11 (0.74;1.66)	0.6102	1.05 (0.70;1.57)	0.8096
**Dairy[a]**	**T2**	1.17 (0.80;1.71)	0.4160	0.99 (0.67;1.45)	0.9548	1.08 (0.73;1.60)	0.6940	1.12 (0.77;1.63)	0.5420	1.02 (0.70;1.49)	0.9314	0.95 (0.64;1.41)	0.8061
	**T3**	0.93 (0.64;1.37)	0.7242	0.78 (0.53;1.15)	0.2133	1.01 (0.68;1.50)	0.9689	0.94 (0.64;1.37)	0.7351	0.94 (0.63;1.40)	0.7604	1.35 (0.91;2.01)	0.1296
**Sugar-sweetend**	**T2**	1.00 (0.69;1.46)	1.0000	1.27 (0.87;1.86)	0.2205	1.06 (0.72;1.56)	0.7653	1.44 (0.99;2.11)	0.0588	0.87 (0.60;1.28)	0.4931	0.59 (0.40;0.88)	0.0090
**food[a]**	**T3**	1.12 (0.77;1.65)	0.5473	1.19 (0.80;1.76)	0.3907	1.14 (0.76;1.70)	0.5191	1.72 (1.17;2.53)	0.0057	0.74 (0.50;1.10)	0.1376	0.66 (0.45;0.98)	0.0397
**Caloric drinks[a]**	**T2**	0.76 (0.52;1.11)	0.1498	0.99 (0.68;1.46)	0.9740	0.95 (0.64;1.41)	0.7839	1.08 (0.75;1.56)	0.6834	0.99 (0.68;1.46)	0.9755	0.85 (0.57;1.25)	0.3999
	**T3**	0.66 (0.45;0.97)	0.0322	0.76 (0.52;1.12)	0.1704	0.79 (0.54;1.17)	0.2428	0.61 (0.41;0.89)	0.0108	0.68 (0.46;1.01)	0.0545	0.68 (0.46;1.01)	0.0575
**Water[a] [ml/d]**	**T2**	1.21 (0.82;1.77)	0.3334	1.18 (0.80;1.74)	0.4055	1.04 (0.70;1.56)	0.8399	1.64 (1.12;2.41)	0.0109	0.94 (0.63;1.41)	0.7782	1.56 (1.06;2.31)	0.0251
	**T3**	1.24 (0.78;1.97)	0.3661	1.25 (0.79;1.99)	0.3373	1.30 (0.81;2.07)	0.2785	1.59 (1.00;2.54)	0.0513	1.86 (1.17;2.96)	0.0089	1.60 (0.99;2.59)	0.0560
**Tea[a] [ml/d]**	**T2**	1.16 (0.79;1.70)	0.4498	0.81 (0.55;1.19)	0.2779	1.04 (0.68;1.57)	0.8678	0.69 (0.47;1.00)	0.0496	1.04 (0.71;1.53)	0.8322	1.02 (0.69;1.52)	0.9188
	**T3**	1.11 (0.73;1.68)	0.6137	0.91 (0.61;1.37)	0.6623	1.58 (1.03;2.42)	0.0376	0.75 (0.50;1.12)	0.1569	0.86 (0.56;1.30)	0.4683	1.20 (0.79;1.82)	0.3880

Table S6. Association between tertiles of eating behaviours and dietary intake in females at the 15-year follow-up *(Continued)*

	**External eating (N=1,000)**				**Emotional eating (N=1,000)**				**Dietary restraint (N=1,000)**			
	**T2 (n=315) Score=6-9**		**T3 (n=313) Score=10-22**		**T2 (n=236) Score=3-5**		**T3 (n=305) Score=6-24**		**T2 (n=277) Score=3-6**		**T3 (n=303) Score=7-21**	
	**Estimate (95% CI)**	**P-value**	**Estimate (95% CI)**	**P-value**	**Estimate (95% CI)**	**P-value**	**Estimate (95% CI)**	**P-value**	**Estimate (95% CI)**	**P-value**	**Estimate (95% CI)**	**P-value**
**Total energy[b] [kJ/d]**	409 (39;779)	0.0302	718 (346;1,090)	**0.0002**	-90 (-476;297)	0.6492	511 (146;875)	0.0061	-371 (-747;4)	0.0527	-967 (-1,343;-591)	**<0.0001**
**Fat[b]**	0.89 (-0.03;1.81)	0.0575	0.33 (-0.60;1.27)	0.4826	-0.13 (-1.09;0.83)	0.7845	0.37 (-0.54;1.28)	0.4285	-0.50 (-1.44;0.44)	0.2973	-0.28 (-1.23;0.68)	0.5691
**Protein[b]**	-0.11 (-0.55;0.32)	0.6118	-0.27 (-0.71;0.17)	0.2331	-0.18 (-0.63;0.28)	0.4482	-0.42 (-0.85;0.01)	0.0535	-0.14 (-0.58;0.31)	0.5473	0.29 (-0.16;0.74)	0.2127
**Carbohydrate[b]**	-0.75 (-1.88;0.38)	0.1932	-0.01 (-1.16;1.13)	0.9795	0.33 (-0.85;1.50)	0.5850	0.07 (-1.04;1.18)	0.9046	0.66 (-0.49;1.82)	0.2598	-0.04 (-1.20;1.13)	0.9500
**Fibres[b] [g/d]**	-0.37 (-1.18;0.44)	0.3704	0.63 (-0.19;1.45)	0.1334	-0.26 (-1.10;0.58)	0.5434	0.36 (-0.43;1.16)	0.3710	0.82 (-0.01;1.64)	0.0525	0.86 (0.02;1.69)	0.0447
**Total sugar[b]**	-0.71 (-1.95;0.53)	0.2606	-1.15 (-2.40;0.11)	0.0731	-0.09 (-1.38;1.20)	0.8882	0.10 (-1.12;1.32)	0.8744	-0.67 (-1.94;0.59)	0.2963	0.73 (-0.55;2.01)	0.2642
**SFA[b]**	0.51 (0.07;0.96)	0.0237	0.27 (-0.18;0.72)	0.2352	-0.17 (-0.63;0.30)	0.4840	0.34 (-0.09;0.78)	0.1240	-0.29 (-0.74;0.17)	0.2136	-0.23 (-0.69;0.24)	0.3367
**MUFA[b]**	0.32 (-0.06;0.71)	0.1013	0.12 (-0.27;0.52)	0.5340	-0.02 (-0.42;0.38)	0.9239	0.08 (-0.30;0.46)	0.6806	-0.05 (-0.45;0.34)	0.7951	-0.05 (-0.45;0.36)	0.8253
**PUFA[c]**	1.01 (0.97;1.05)	0.6396	0.99 (0.95;1.03)	0.5971	1.01 (0.97;1.05)	0.6969	0.99 (0.95;1.03)	0.5084	0.98 (0.94;1.02)	0.2428	1.00 (0.96;1.04)	0.8710
**omega-3 PUFA[c]**	1.03 (0.99;1.06)	0.1424	1.01 (0.98;1.05)	0.4957	0.99 (0.96;1.03)	0.7759	1.00 (0.97;1.04)	0.9826	0.94 (0.91;0.98)	**0.0017**	0.98 (0.95;1.02)	0.3266
**omega-6 PUFA[c]**	1.01 (0.97;1.05)	0.7303	0.99 (0.95;1.03)	0.5557	1.01 (0.97;1.06)	0.6391	0.99 (0.95;1.03)	0.4996	0.98 (0.94;1.02)	0.3934	1.00 (0.96;1.04)	0.9378

Abbreviations: CI, confidence interval; MUFA, monounsaturated fatty acids; PUFA, polyunsaturated fatty acids; SFA, saturated fatty acids; T2, tertile 2; T3, tertile 3. [a]Effect estimates of multinomial logistic regression are presented as relative risk ratio (95% CI). [b]Effect estimates of multiple linear regression are presented as beta coefficient (95% CI). [c]Effect estimates of multiple linear regression for naturally log-transformed outcome variables are presented as means ratio (95% CI). All models were adjusted for age, BMI, pubertal status, siblings, moderate-to-vigorous physical activity, screen time, total difficulties, parental education, parental BMI, study, and recruitment region. Food groups (except water and tea) and nutrients models were further adjusted for total daily energy intake. Water and Tea models were further adjusted for total daily beverage intake. Tertile 1 is the reference category. Significant associations are marked in bold: p<0.0019.

Table S7. Association between tertiles of eating behaviours and dietary intake in males at the 15-year follow-up

		External eating (N=880)				Emotional eating (N=880)				Dietary restraint (N=880)			
		T2 (n=332) Score=5-9		T3 (n=249) Score=10-22		T2 (n=310) Score=1-3		T3 (n=239) Score=4-23		T2 (n=366) Score=1-3		T3 (n=218) Score=4-21	
		Estimate (95% CI)	P-value	Estimate (95% CI)	P-value	Estimate (95% CI)	P-value	Estimate (95% CI)	P-value	Estimate (95% CI)	P-value	Estimate (95% CI)	P-value
Fruit[a]	T2	1.01 (0.68;1.50)	0.9759	1.05 (0.68;1.61)	0.8273	0.70 (0.48;1.04)	0.0814	0.90 (0.58;1.39)	0.6379	1.19 (0.81;1.75)	0.3794	1.19 (0.74;1.91)	0.4769
	T3	1.06 (0.70;1.60)	0.7840	0.87 (0.55;1.37)	0.5412	0.68 (0.45;1.03)	0.0699	0.91 (0.58;1.43)	0.6784	1.31 (0.87;1.97)	0.1998	1.49 (0.91;2.46)	0.1147
Vegetable[a]	T2	1.00 (0.67;1.47)	0.9853	1.13 (0.74;1.74)	0.5631	0.96 (0.65;1.41)	0.8178	1.11 (0.72;1.70)	0.6294	1.18 (0.80;1.72)	0.4061	1.19 (0.74;1.92)	0.4771
	T3	0.99 (0.66;1.49)	0.9664	0.94 (0.60;1.47)	0.7821	0.79 (0.53;1.19)	0.2580	0.93 (0.60;1.47)	0.7685	1.19 (0.79;1.79)	0.3991	1.60 (0.98;2.60)	0.0581
Starchy	T2	1.04 (0.69;1.55)	0.8596	1.00 (0.65;1.54)	0.9958	1.50 (1.00;2.24)	0.0519	1.02 (0.66;1.57)	0.9407	0.94 (0.63;1.40)	0.7615	0.98 (0.61;1.58)	0.9268
vegetables[a]	T3	1.08 (0.71;1.64)	0.7189	0.90 (0.57;1.42)	0.6465	1.05 (0.69;1.59)	0.8339	0.72 (0.46;1.14)	0.1582	1.04 (0.68;1.58)	0.8622	1.14 (0.69;1.88)	0.6175
Whole grains[a]	T2	0.96 (0.64;1.42)	0.8212	0.57 (0.37;0.89)	0.0127	0.76 (0.51;1.12)	0.1634	0.58 (0.38;0.90)	0.0157	0.76 (0.51;1.12)	0.1645	0.99 (0.61;1.60)	0.9672
	T3	0.98 (0.66;1.48)	0.9384	0.66 (0.42;1.02)	0.0610	0.74 (0.50;1.12)	0.1531	0.75 (0.49;1.16)	0.1950	0.72 (0.48;1.08)	0.1121	1.14 (0.70;1.85)	0.5950
Refined grains[a]	T2	0.94 (0.63;1.40)	0.7647	0.81 (0.52;1.26)	0.3459	1.25 (0.84;1.86)	0.2792	0.70 (0.45;1.08)	0.1090	1.20 (0.80;1.80)	0.3673	1.35 (0.84;2.16)	0.2130
	T3	0.82 (0.55;1.22)	0.3285	0.95 (0.61;1.47)	0.8055	1.57 (1.04;2.35)	0.0307	1.05 (0.68;1.62)	0.8346	0.94 (0.63;1.39)	0.7577	0.71 (0.44;1.16)	0.1688
Meat[a]	T2	0.86 (0.58;1.27)	0.4361	0.90 (0.59;1.38)	0.6317	1.03 (0.70;1.53)	0.8779	0.93 (0.61;1.43)	0.7471	0.93 (0.63;1.36)	0.6943	0.96 (0.60;1.56)	0.8806
	T3	1.20 (0.81;1.79)	0.3606	1.03 (0.66;1.60)	0.9104	1.04 (0.70;1.55)	0.8332	0.83 (0.54;1.28)	0.4026	0.83 (0.56;1.23)	0.3454	1.10 (0.68;1.77)	0.6940
Fish[a]	T2	1.23 (0.83;1.82)	0.3031	1.77 (1.15;2.73)	0.0102	0.82 (0.55;1.20)	0.3066	1.05 (0.68;1.63)	0.8325	1.30 (0.88;1.92)	0.1802	1.23 (0.76;1.99)	0.4002
	T3	1.53 (1.04;2.26)	0.0320	1.91 (1.23;2.96)	0.0039	0.86 (0.58;1.27)	0.4371	1.44 (0.94;2.22)	0.0975	0.99 (0.67;1.46)	0.9520	1.38 (0.87;2.20)	0.1742
Eggs[a]	T2	1.49 (1.00;2.22)	0.0473	1.32 (0.86;2.03)	0.2091	1.23 (0.83;1.81)	0.3055	1.35 (0.88;2.09)	0.1708	1.20 (0.82;1.76)	0.3419	1.57 (0.97;2.54)	0.0667
	T3	1.36 (0.91;2.02)	0.1336	1.16 (0.75;1.79)	0.5160	1.05 (0.71;1.57)	0.7941	1.27 (0.82;1.96)	0.2910	1.35 (0.91;2.02)	0.1375	1.94 (1.20;3.14)	0.0072
Nuts and seeds[a]	T2	1.01 (0.68;1.48)	0.9788	1.26 (0.82;1.94)	0.2929	1.29 (0.88;1.89)	0.1983	1.23 (0.80;1.90)	0.3400	1.06 (0.72;1.55)	0.7729	1.69 (1.05;2.73)	0.0315
	T3	0.97 (0.65;1.43)	0.8651	1.15 (0.75;1.78)	0.5261	1.42 (0.95;2.10)	0.0858	1.66 (1.08;2.55)	0.0218	1.20 (0.81;1.77)	0.3632	2.07 (1.28;3.36)	0.0032
Butter[a]	T2	1.45 (0.96;2.18)	0.0757	1.20 (0.77;1.87)	0.4317	0.96 (0.63;1.44)	0.8299	0.98 (0.63;1.54)	0.9446	1.57 (1.04;2.37)	0.0318	1.59 (0.97;2.61)	0.0676
	T3	1.96 (1.28;2.99)	**0.0019**	1.33 (0.84;2.12)	0.2296	0.95 (0.62;1.45)	0.8097	0.91 (0.57;1.44)	0.6798	1.14 (0.75;1.73)	0.5416	1.39 (0.84;2.30)	0.1962
Margarine[a]	T2	1.08 (0.72;1.63)	0.6991	0.90 (0.58;1.40)	0.6436	0.83 (0.55;1.26)	0.3806	1.12 (0.72;1.73)	0.6151	0.92 (0.62;1.38)	0.6992	1.26 (0.77;2.05)	0.3596
	T3	1.23 (0.81;1.86)	0.3284	1.05 (0.67;1.65)	0.8412	0.98 (0.65;1.47)	0.9282	0.96 (0.61;1.52)	0.8779	0.89 (0.59;1.34)	0.5867	1.09 (0.67;1.78)	0.7366
Oils[a]	T2	1.04 (0.70;1.54)	0.8376	0.97 (0.62;1.50)	0.8874	1.08 (0.73;1.59)	0.7143	1.06 (0.69;1.62)	0.7956	0.77 (0.52;1.14)	0.1917	0.58 (0.36;0.93)	0.0251
	T3	0.75 (0.50;1.13)	0.1742	0.86 (0.55;1.34)	0.5061	1.06 (0.71;1.59)	0.7597	1.04 (0.66;1.62)	0.8752	0.94 (0.62;1.41)	0.7620	0.90 (0.56;1.46)	0.6667
Dairy[a]	T2	1.17 (0.79;1.73)	0.4472	0.88 (0.58;1.36)	0.5758	1.62 (1.09;2.41)	0.0164	1.38 (0.90;2.12)	0.1421	1.13 (0.76;1.67)	0.5436	1.18 (0.74;1.89)	0.4884
	T3	0.87 (0.59;1.30)	0.5085	0.74 (0.48;1.14)	0.1699	1.03 (0.69;1.53)	0.8925	0.91 (0.59;1.40)	0.6551	0.86 (0.58;1.27)	0.4405	0.95 (0.59;1.53)	0.8346
Sugar-sweetend	T2	1.34 (0.91;1.98)	0.1356	1.01 (0.65;1.56)	0.9703	1.52 (1.03;2.24)	0.0346	1.36 (0.88;2.11)	0.1624	0.78 (0.53;1.16)	0.2205	0.80 (0.50;1.27)	0.3434
food[a]	T3	1.04 (0.70;1.54)	0.8560	1.26 (0.82;1.92)	0.2903	1.16 (0.78;1.72)	0.4624	1.45 (0.95;2.22)	0.0891	0.91 (0.61;1.35)	0.6327	0.76 (0.47;1.22)	0.2504
Caloric drinks[a]	T2	0.97 (0.66;1.43)	0.8969	0.67 (0.43;1.04)	0.0714	1.02 (0.69;1.50)	0.9333	0.71 (0.46;1.10)	0.1216	0.86 (0.58;1.28)	0.4621	0.66 (0.42;1.06)	0.0828
	T3	1.03 (0.69;1.53)	0.9008	1.23 (0.80;1.88)	0.3472	0.90 (0.61;1.35)	0.6178	0.91 (0.60;1.39)	0.6653	1.13 (0.76;1.68)	0.5451	0.77 (0.48;1.23)	0.2726
Water[a] [ml/d]	T2	1.37 (0.91;2.05)	0.1307	0.98 (0.63;1.52)	0.9354	1.05 (0.70;1.58)	0.7965	0.93 (0.60;1.45)	0.7574	1.33 (0.90;1.99)	0.1572	1.93 (1.17;3.17)	0.0096
	T3	1.22 (0.78;1.93)	0.3847	0.86 (0.52;1.41)	0.5443	0.89 (0.57;1.41)	0.6262	0.97 (0.59;1.59)	0.9059	0.93 (0.59;1.45)	0.7415	1.80 (1.05;3.10)	0.0327
Tea[a] [ml/d]	T2	1.72 (1.14;2.60)	0.0098	1.66 (1.07;2.60)	0.0248	1.34 (0.89;2.00)	0.1579	1.45 (0.93;2.27)	0.0991	0.93 (0.62;1.38)	0.7052	1.32 (0.81;2.13)	0.2625
	T3	1.42 (0.94;2.14)	0.0990	1.10 (0.70;1.73)	0.6859	1.21 (0.80;1.84)	0.3606	1.51 (0.96;2.37)	0.0766	1.10 (0.73;1.66)	0.6374	1.11 (0.67;1.84)	0.6782

Table S7. Association between tertiles of eating behaviours and dietary intake in males at the 15-year follow-up *(Continued)*

	External eating (N=880)				Emotional eating (N=880)				Dietary restraint (N=880)			
	T2 (n=332) Score=5-9		T3 (n=249) Score=10-22		T2 (n=310) Score=1-3		T3 (n=239) Score=4-23		T2 (n=366) Score=1-3		T3 (n=218) Score=4-21	
	Estimate (95% CI)	P-value	Estimate (95% CI)	P-value	Estimate (95% CI)	P-value	Estimate (95% CI)	P-value	Estimate (95% CI)	P-value	Estimate (95% CI)	P-value
**Total energy[b] [kJ/d]**	234 (-206;675)	0.2967	300 (-184;783)	0.2237	-59 (-498;381)	0.7941	337 (-145;819)	0.1710	-284 (-721;153)	0.2023	-720 (-1,244;-196)	0.0071
**Fat[b]**	0.79 (-0.10;1.69)	0.0834	0.37 (-0.61;1.35)	0.4595	0.18 (-0.72;1.07)	0.6935	0.77 (-0.21;1.76)	0.1219	-0.22 (-1.12;0.67)	0.6209	0.62 (-0.46;1.69)	0.2587
**Protein[b]**	0.04 (-0.41;0.48)	0.8738	-0.45 (-0.93;0.04)	0.0705	-0.28 (-0.72;0.16)	0.2167	-0.27 (-0.76;0.21)	0.2670	-0.01 (-0.45;0.43)	0.9495	0.49 (-0.04;1.01)	0.0720
**Carbohydrate[b]**	-0.80 (-1.92;0.33)	0.1657	0.09 (-1.14;1.32)	0.8860	0.10 (-1.02;1.23)	0.8597	-0.50 (-1.73;0.73)	0.4267	0.19 (-0.93;1.31)	0.7432	-1.13 (-2.47;0.22)	0.0999
**Fibres[b] [g/d]**	0.08 (-0.88;1.04)	0.8750	-0.59 (-1.64;0.46)	0.2723	-0.16 (-1.12;0.80)	0.7455	-1.04 (-2.09;0.01)	0.0532	-0.15 (-1.10;0.81)	0.7636	0.93 (-0.22;2.08)	0.1119
**Total sugar[b]**	-0.44 (-1.70;0.83)	0.4983	0.49 (-0.89;1.87)	0.4869	-0.51 (-1.77;0.75)	0.4277	0.20 (-1.18;1.58)	0.7742	0.69 (-0.56;1.95)	0.2782	-0.45 (-1.96;1.06)	0.5572
**SFA[b]**	0.50 (0.06;0.94)	0.0274	0.25 (-0.23;0.73)	0.3069	0.03 (-0.41;0.47)	0.9039	0.38 (-0.10;0.86)	0.1227	-0.11 (-0.55;0.32)	0.6088	0.19 (-0.33;0.72)	0.4706
**MUFA[b]**	0.28 (-0.10;0.66)	0.1500	0.06 (-0.35;0.48)	0.7637	0.05 (-0.34;0.43)	0.8135	0.17 (-0.25;0.59)	0.4265	-0.04 (-0.42;0.34)	0.8448	0.31 (-0.14;0.77)	0.1800
**PUFA[c]**	1.00 (0.96;1.04)	0.9925	1.01 (0.96;1.05)	0.7586	1.02 (0.98;1.06)	0.2637	1.04 (0.99;1.08)	0.0965	0.98 (0.95;1.02)	0.4177	1.02 (0.97;1.07)	0.4399
**omega-3 PUFA[c]**	1.05 (1.01;1.09)	0.0158	1.07 (1.02;1.11)	0.0022	1.01 (0.97;1.04)	0.7779	1.05 (1.01;1.09)	0.0152	0.99 (0.95;1.03)	0.5796	1.00 (0.96;1.05)	0.8274
**omega-6 PUFA[c]**	0.99 (0.95;1.04)	0.7808	1.00 (0.95;1.05)	0.9721	1.03 (0.98;1.07)	0.2443	1.03 (0.99;1.08)	0.1422	0.98 (0.94;1.02)	0.3996	1.02 (0.97;1.07)	0.4376

Abbreviations: CI, confidence interval; MUFA, monounsaturated fatty acids; PUFA, polyunsaturated fatty acids; SFA, saturated fatty acids; T2, tertile 2; T3, tertile 3. [a]Effect estimates of multinomial logistic regression are presented as relative risk ratio (95% CI). [b]Effect estimates of multiple linear regression are presented as beta coefficient (95% CI). [c]Effect estimates of multiple linear regression for naturally log-transformed outcome variables are presented as means ratio (95% CI). All models were adjusted for age, BMI, pubertal status, siblings, moderate-to-vigorous physical activity, screen time, total difficulties, parental education, parental BMI, study, and recruitment region. Food groups (except water and tea) and nutrients models were further adjusted for total daily energy intake. Water and Tea models were further adjusted for total daily beverage intake. Tertile 1 is the reference category. Significant associations are marked in bold: p<0.0019.

Table S8. Sensitivity analysis (10 females who reported a vegetarian or vegan diet were excluded): Effect estimates and 95% CI assessing the association between tertiles of eating behaviours and dietary intake in females at the 10-year follow-up

		**External eating (N=1,072)**				**Emotional eating (N=1,072)**			
		**T2 (n=393)**		**T3 (n=300)**		**T2 (n=296)**		**T3 (n=317)**	
		**Score=5-8**		**Score=9-24**		**Score=1-2**		**Score=3-18**	
		**Estimate (95% CI)**	**P-value**	**Estimate (95% CI)**	**P-value**	**Estimate (95% CI)**	**P-value**	**Estimate (95% CI)**	**P-value**
**Fruit[a]**	**T2**	0.91 (0.64;1.31)	0.6281	0.82 (0.55;1.21)	0.3188	1.26 (0.87;1.83)	0.2230	0.91 (0.63;1.30)	0.5902
	**T3**	1.00 (0.69;1.45)	0.9995	0.74 (0.49;1.12)	0.1569	1.46 (0.99;2.16)	0.0546	0.98 (0.67;1.43)	0.9020
**Vegetable[a]**	**T2**	1.17 (0.81;1.69)	0.3942	0.99 (0.67;1.46)	0.9618	1.85 (1.26;2.70)	**0.0015**	0.79 (0.55;1.14)	0.2096
	**T3**	0.96 (0.66;1.38)	0.8130	0.64 (0.43;0.96)	0.0322	1.64 (1.11;2.44)	0.0136	0.83 (0.57;1.20)	0.3158
**Starchy**	**T2**	1.08 (0.75;1.54)	0.6837	1.05 (0.70;1.57)	0.8045	0.90 (0.62;1.30)	0.5718	1.04 (0.72;1.52)	0.8168
**vegetables[a]**	**T3**	0.89 (0.60;1.31)	0.5492	1.29 (0.85;1.96)	0.2329	0.96 (0.65;1.43)	0.8419	1.28 (0.87;1.89)	0.2163
**Whole grains[a]**	**T2**	0.90 (0.63;1.28)	0.5597	0.57 (0.38;0.84)	0.0044	0.84 (0.58;1.20)	0.3373	0.74 (0.51;1.06)	0.1015
	**T3**	1.11 (0.77;1.60)	0.5738	0.71 (0.48;1.06)	0.0955	0.81 (0.56;1.17)	0.2639	0.95 (0.66;1.36)	0.7643
**Refined grains[a]**	**T2**	0.83 (0.58;1.17)	0.2841	0.89 (0.60;1.31)	0.5524	1.08 (0.75;1.56)	0.6740	0.92 (0.64;1.32)	0.6621
	**T3**	1.08 (0.75;1.56)	0.6684	1.26 (0.85;1.87)	0.2583	1.14 (0.79;1.66)	0.4788	0.94 (0.65;1.36)	0.7599
**Meat[a]**	**T2**	1.24 (0.87;1.77)	0.2357	1.26 (0.85;1.87)	0.2424	0.78 (0.54;1.14)	0.1994	1.30 (0.91;1.87)	0.1534
	**T3**	1.08 (0.75;1.54)	0.6815	1.20 (0.81;1.77)	0.3698	1.09 (0.76;1.56)	0.6389	0.98 (0.67;1.43)	0.9285
**Fish[a]**	**T2**	0.93 (0.65;1.32)	0.6794	1.21 (0.82;1.79)	0.3376	1.48 (1.02;2.13)	0.0369	1.04 (0.72;1.50)	0.8251
	**T3**	0.85 (0.59;1.21)	0.3619	1.15 (0.78;1.71)	0.4827	1.24 (0.86;1.81)	0.2520	1.03 (0.72;1.48)	0.8666
**Eggs[a]**	**T2**	1.11 (0.78;1.58)	0.5739	1.18 (0.81;1.74)	0.3876	1.33 (0.92;1.93)	0.1305	1.22 (0.85;1.74)	0.2740
	**T3**	1.20 (0.84;1.71)	0.3197	1.05 (0.71;1.55)	0.8067	1.69 (1.17;2.44)	0.0050	1.18 (0.82;1.70)	0.3792
**Nuts and seeds[a]**	**T2**	0.77 (0.54;1.10)	0.1507	0.77 (0.52;1.13)	0.1839	0.87 (0.60;1.25)	0.4437	0.86 (0.60;1.23)	0.3956
	**T3**	1.08 (0.76;1.54)	0.6742	0.92 (0.62;1.36)	0.6836	0.98 (0.68;1.41)	0.9199	0.95 (0.66;1.37)	0.7883
**Butter[a]**	**T2**	0.97 (0.66;1.42)	0.8818	0.99 (0.65;1.51)	0.9796	1.26 (0.85;1.87)	0.2422	1.18 (0.80;1.75)	0.3973
	**T3**	0.84 (0.56;1.26)	0.3978	0.85 (0.54;1.32)	0.4649	0.93 (0.61;1.42)	0.7479	0.91 (0.60;1.38)	0.6641
**Margarine[a]**	**T2**	1.42 (0.98;2.06)	0.0616	1.08 (0.72;1.63)	0.6990	0.89 (0.60;1.30)	0.5361	1.20 (0.83;1.75)	0.3339
	**T3**	1.03 (0.70;1.52)	0.8816	1.24 (0.81;1.89)	0.3167	1.23 (0.82;1.83)	0.3115	1.40 (0.94;2.10)	0.0972
**Oils[a]**	**T2**	1.15 (0.81;1.65)	0.4308	1.05 (0.71;1.56)	0.7908	1.25 (0.86;1.82)	0.2367	1.12 (0.78;1.61)	0.5255
	**T3**	0.95 (0.66;1.37)	0.7891	0.92 (0.62;1.38)	0.6928	1.71 (1.17;2.50)	0.0053	1.24 (0.85;1.81)	0.2643
**Dairy[a]**	**T2**	0.75 (0.52;1.07)	0.1170	1.02 (0.69;1.50)	0.9148	0.98 (0.68;1.42)	0.9295	1.15 (0.80;1.65)	0.4462
	**T3**	0.79 (0.55;1.13)	0.1921	0.77 (0.51;1.15)	0.1941	0.74 (0.51;1.07)	0.1111	0.72 (0.50;1.05)	0.0856
**Sugar-sweetend**	**T2**	1.52 (1.06;2.18)	0.0213	1.64 (1.12;2.42)	0.0119	1.39 (0.97;1.99)	0.0691	1.37 (0.94;1.98)	0.1005
**food[a]**	**T3**	1.53 (1.07;2.18)	0.0183	1.38 (0.93;2.04)	0.1102	0.93 (0.64;1.36)	0.7209	1.61 (1.12;2.31)	0.0100
**Caloric drinks[a]**	**T2**	1.23 (0.86;1.76)	0.2522	1.29 (0.87;1.92)	0.2024	1.18 (0.82;1.70)	0.3818	0.98 (0.68;1.41)	0.9056
	**T3**	0.85 (0.60;1.21)	0.3720	1.01 (0.69;1.49)	0.9537	1.03 (0.71;1.48)	0.8896	0.95 (0.67;1.37)	0.8028
**Water[a] [ml/d]**	**T2**	1.28 (0.89;1.84)	0.1853	1.03 (0.70;1.53)	0.8784	0.84 (0.58;1.21)	0.3513	1.05 (0.73;1.53)	0.7814
	**T3**	1.18 (0.80;1.74)	0.4021	0.79 (0.51;1.21)	0.2735	0.82 (0.55;1.22)	0.3230	0.93 (0.62;1.40)	0.7325
**Tea[a] [ml/d]**	**T2**	1.37 (0.96;1.96)	0.0865	1.18 (0.79;1.75)	0.4205	0.82 (0.57;1.18)	0.2786	1.11 (0.77;1.61)	0.5710
	**T3**	0.98 (0.67;1.43)	0.9051	0.98 (0.65;1.48)	0.9304	0.80 (0.54;1.17)	0.2490	0.93 (0.63;1.37)	0.7110
**Total energy[b] [kJ/d]**		208 (-81;497)	0.1584	401 (85;717)	0.0130	42 (-257;340)	0.7836	210 (-86;506)	0.1634
**Fat[b]**		-0.08 (-0.86;0.70)	0.8407	0.02 (-0.83;0.88)	0.9568	0.06 (-0.74;0.87)	0.8765	-0.18 (-0.98;0.62)	0.6532
**Protein[b]**		0.02 (-0.32;0.36)	0.9221	0.05 (-0.32;0.42)	0.7909	0.00 (-0.35;0.35)	0.9898	-0.24 (-0.59;0.11)	0.1758
**Carbohydrate[b]**		0.08 (-0.85;1.01)	0.8711	-0.07 (-1.09;0.95)	0.8990	-0.08 (-1.04;0.88)	0.8692	0.43 (-0.52;1.38)	0.3704
**Fibres[b] [g/d]**		-0.03 (-0.70;0.63)	0.9251	-0.76 (-1.49;-0.03)	0.0423	0.36 (-0.32;1.05)	0.2992	-0.37 (-1.05;0.31)	0.2905
**Total sugar[b]**		-0.26 (-1.28;0.76)	0.6192	-0.17 (-1.30;0.95)	0.7602	0.04 (-1.02;1.09)	0.9451	0.58 (-0.47;1.62)	0.2769
**SFA[b]**		-0.04 (-0.43;0.35)	0.8301	-0.06 (-0.49;0.37)	0.7855	-0.22 (-0.62;0.18)	0.2735	-0.18 (-0.58;0.22)	0.3735
**MUFA[b]**		0.01 (-0.32;0.35)	0.9308	0.10 (-0.27;0.47)	0.5911	0.06 (-0.28;0.41)	0.7151	-0.07 (-0.41;0.27)	0.6890
**PUFA[c]**		0.99 (0.96;1.03)	0.6687	1.00 (0.96;1.04)	0.9236	1.04 (1.00;1.08)	0.0366	1.02 (0.98;1.06)	0.2780
**omega-3 PUFA[c]**		0.99 (0.96;1.02)	0.5092	1.01 (0.98;1.04)	0.4540	1.04 (1.01;1.07)	0.0156	1.01 (0.98;1.04)	0.4166
**omega-6 PUFA[c]**		0.99 (0.96;1.03)	0.7177	1.00 (0.96;1.04)	0.9858	1.04 (1.00;1.08)	0.0529	1.02 (0.98;1.06)	0.2826

Abbreviations: CI, confidence interval; MUFA, monounsaturated fatty acids; PUFA, polyunsaturated fatty acids; SFA, saturated fatty acids; T2, tertile 2; T3, tertile 3. [a]Effect estimates of multinomial logistic regression are presented as relative risk ratio (95% CI). [b]Effect estimates of multiple linear regression are presented as beta coefficient (95% CI). [c]Effect estimates of multiple linear regression for naturally log-transformed outcome variables are presented as means ratio (95% CI). All models were adjusted for age, BMI, pubertal status, siblings, moderate-to-vigorous physical activity, screen time, total difficulties, parental education, parental BMI, study, and recruitment region. Food groups (except water and tea) and nutrients models were further adjusted for total daily energy intake. Water and Tea models were further adjusted for total daily beverage intake. Tertile 1 is the reference category. Significant associations are marked in bold: $p<0.0019$.

Table S9. Sensitivity analysis (4 males who reported a vegetarian or vegan diet were excluded): Effect estimates and 95% CI assessing the association between tertiles of eating behaviours and dietary intake in males at the 10-year follow-up

		External eating (N=1,171)				Emotional eating (N=1,171)			
		T2 (n=375) Score=5-8		T3 (n=313) Score=9-24		T2 (n=333) Score=1-2		T3 (n=353) Score=3-18	
		Estimate (95% CI)	P-value	Estimate (95% CI)	P-value	Estimate (95% CI)	P-value	Estimate (95% CI)	P-value
Fruit[a]	T2	0.82 (0.58;1.15)	0.2489	0.92 (0.64;1.32)	0.6579	0.97 (0.69;1.38)	0.8768	1.05 (0.74;1.51)	0.7705
	T3	0.93 (0.66;1.33)	0.6976	0.94 (0.64;1.38)	0.7561	1.04 (0.72;1.50)	0.8473	1.33 (0.92;1.92)	0.1328
Vegetable[a]	T2	1.30 (0.92;1.82)	0.1332	1.02 (0.71;1.47)	0.8982	0.86 (0.60;1.22)	0.3902	1.08 (0.76;1.53)	0.6710
	T3	0.98 (0.69;1.39)	0.9088	1.03 (0.72;1.49)	0.8577	1.13 (0.79;1.62)	0.5060	1.17 (0.81;1.69)	0.3959
Starchy	T2	0.85 (0.60;1.21)	0.3697	1.09 (0.76;1.57)	0.6495	0.82 (0.57;1.18)	0.2848	0.97 (0.68;1.38)	0.8784
vegetables[a]	T3	0.90 (0.62;1.30)	0.5677	1.10 (0.74;1.64)	0.6476	1.08 (0.73;1.58)	0.7080	1.06 (0.72;1.57)	0.7518
Whole grains[a]	T2	1.23 (0.87;1.74)	0.2392	1.35 (0.94;1.94)	0.1073	1.05 (0.73;1.49)	0.8019	1.30 (0.91;1.85)	0.1507
	T3	1.06 (0.74;1.49)	0.7633	0.87 (0.60;1.27)	0.4801	1.12 (0.78;1.60)	0.5482	1.14 (0.79;1.65)	0.4804
Refined grains[a]	T2	1.13 (0.81;1.59)	0.4738	1.25 (0.87;1.79)	0.2310	0.99 (0.69;1.40)	0.9359	1.27 (0.90;1.80)	0.1741
	T3	0.94 (0.67;1.33)	0.7310	0.98 (0.68;1.42)	0.9271	0.95 (0.67;1.34)	0.7553	0.81 (0.56;1.16)	0.2434
Meat[a]	T2	1.02 (0.73;1.43)	0.9239	0.91 (0.64;1.31)	0.6254	0.99 (0.70;1.40)	0.9590	0.91 (0.64;1.29)	0.5827
	T3	1.30 (0.93;1.83)	0.1295	1.25 (0.87;1.80)	0.2182	1.10 (0.77;1.57)	0.5891	1.20 (0.85;1.71)	0.2988
Fish[a]	T2	1.01 (0.72;1.41)	0.9533	1.19 (0.83;1.72)	0.3507	0.82 (0.58;1.16)	0.2546	1.01 (0.71;1.44)	0.9503
	T3	0.98 (0.69;1.37)	0.8940	1.35 (0.94;1.95)	0.1049	0.89 (0.62;1.26)	0.4971	1.06 (0.74;1.51)	0.7449
Eggs[a]	T2	0.93 (0.66;1.30)	0.6576	0.96 (0.67;1.38)	0.8356	1.26 (0.88;1.80)	0.2135	0.84 (0.59;1.19)	0.3188
	T3	0.98 (0.69;1.37)	0.8915	0.92 (0.64;1.32)	0.6471	1.57 (1.10;2.25)	0.0136	1.00 (0.70;1.42)	0.9968
Nuts and seeds[a]	T2	1.11 (0.79;1.56)	0.5374	1.25 (0.87;1.79)	0.2322	1.14 (0.80;1.61)	0.4620	0.88 (0.62;1.25)	0.4819
	T3	0.94 (0.67;1.32)	0.7339	1.07 (0.74;1.54)	0.7191	1.21 (0.85;1.72)	0.3009	1.18 (0.83;1.67)	0.3488
Butter[a]	T2	1.15 (0.80;1.66)	0.4475	1.19 (0.80;1.77)	0.3866	1.50 (1.03;2.20)	0.0362	1.10 (0.75;1.61)	0.6108
	T3	1.18 (0.80;1.73)	0.3986	1.31 (0.87;1.97)	0.2002	1.41 (0.95;2.10)	0.0883	0.98 (0.66;1.46)	0.9220
Margarine[a]	T2	0.73 (0.51;1.04)	0.0831	1.06 (0.74;1.53)	0.7445	0.84 (0.58;1.21)	0.3524	1.12 (0.78;1.60)	0.5335
	T3	0.90 (0.62;1.30)	0.5700	0.68 (0.46;1.02)	0.0654	1.05 (0.72;1.53)	0.8067	1.05 (0.71;1.55)	0.8045
Oils[a]	T2	0.98 (0.70;1.39)	0.9269	0.97 (0.67;1.41)	0.8684	0.86 (0.60;1.23)	0.4176	1.23 (0.86;1.77)	0.2582
	T3	1.31 (0.91;1.87)	0.1420	1.48 (1.01;2.16)	0.0445	0.99 (0.69;1.42)	0.9464	1.29 (0.89;1.87)	0.1828
Dairy[a]	T2	0.73 (0.52;1.03)	0.0749	0.67 (0.46;0.96)	0.0279	0.74 (0.52;1.05)	0.0939	0.86 (0.60;1.22)	0.3967
	T3	0.78 (0.55;1.11)	0.1726	0.69 (0.48;1.01)	0.0553	0.86 (0.60;1.23)	0.4156	0.91 (0.63;1.31)	0.6172
Sugar-sweetend	T2	1.28 (0.92;1.80)	0.1457	1.05 (0.73;1.52)	0.7900	1.18 (0.83;1.68)	0.3594	1.05 (0.74;1.49)	0.7853
food[a]	T3	1.03 (0.73;1.46)	0.8671	1.40 (0.97;2.01)	0.0702	1.24 (0.87;1.77)	0.2438	1.13 (0.79;1.61)	0.5097
Caloric drinks[a]	T2	0.85 (0.60;1.19)	0.3331	0.91 (0.64;1.31)	0.6196	0.89 (0.63;1.26)	0.5117	0.75 (0.53;1.06)	0.1065
	T3	1.08 (0.77;1.52)	0.6558	0.99 (0.69;1.42)	0.9515	1.00 (0.71;1.43)	0.9803	0.74 (0.52;1.06)	0.1010
Water[a] [ml/d]	T2	1.35 (0.95;1.91)	0.0942	1.35 (0.93;1.96)	0.1168	0.88 (0.62;1.26)	0.4889	0.81 (0.56;1.16)	0.2477
	T3	1.01 (0.70;1.47)	0.9493	1.14 (0.77;1.70)	0.5147	0.85 (0.58;1.25)	0.4104	1.19 (0.82;1.74)	0.3642
Tea[a] [ml/d]	T2	1.01 (0.71;1.43)	0.9759	1.16 (0.79;1.70)	0.4456	1.02 (0.70;1.46)	0.9351	1.05 (0.73;1.51)	0.8116
	T3	0.93 (0.64;1.34)	0.6781	1.17 (0.79;1.75)	0.4260	1.31 (0.90;1.90)	0.1657	1.08 (0.73;1.59)	0.7023
Total energy[b] [kJ/d]		238 (-93;569)	0.1588	253 (-100;606)	0.1594	456 (116;796)	0.0086	437 (96;778)	0.0122
Fat[b]		0.67 (-0.09;1.43)	0.0819	0.95 (0.14;1.76)	0.0209	0.34 (-0.44;1.13)	0.3895	0.70 (-0.09;1.48)	0.0824
Protein[b]		-0.10 (-0.43;0.23)	0.5550	-0.17 (-0.52;0.19)	0.3569	0.03 (-0.31;0.37)	0.8659	0.20 (-0.14;0.55)	0.2427
Carbohydrate[b]		-0.56 (-1.47;0.34)	0.2192	-0.77 (-1.73;0.19)	0.1146	-0.38 (-1.31;0.56)	0.4287	-0.90 (-1.83;0.03)	0.0591
Fibres[b] [g/d]		0.03 (-0.68;0.75)	0.9275	0.00 (-0.76;0.76)	0.9903	0.40 (-0.33;1.14)	0.2846	0.45 (-0.29;1.19)	0.2319
Total sugar[b]		-0.17 (-1.15;0.82)	0.7407	-0.51 (-1.56;0.54)	0.3371	-0.20 (-1.22;0.82)	0.6990	-0.62 (-1.64;0.39)	0.2293
SFA[b]		0.27 (-0.11;0.64)	0.1625	0.37 (-0.03;0.77)	0.0682	0.07 (-0.32;0.45)	0.7328	0.19 (-0.20;0.57)	0.3470
MUFA[b]		0.30 (-0.02;0.62)	0.0643	0.38 (0.04;0.72)	0.0304	0.16 (-0.17;0.49)	0.3451	0.28 (-0.05;0.61)	0.0945
PUFA[c]		1.02 (0.98;1.05)	0.2984	1.03 (0.99;1.07)	0.1242	1.02 (0.99;1.06)	0.2065	1.04 (1.00;1.07)	0.0423
omega-3 PUFA[c]		1.00 (0.97;1.02)	0.7575	1.03 (1.00;1.06)	0.0847	1.00 (0.97;1.03)	0.8565	1.03 (1.00;1.07)	0.0268
omega-6 PUFA[c]		1.02 (0.99;1.06)	0.2455	1.03 (0.99;1.07)	0.1546	1.03 (0.99;1.07)	0.1810	1.04 (1.00;1.08)	0.0582

Abbreviations: CI, confidence interval; MUFA, monounsaturated fatty acids; PUFA, polyunsaturated fatty acids; SFA, saturated fatty acids; T2, tertile 2; T3, tertile 3. [a]Effect estimates of multinomial logistic regression are presented as relative risk ratio (95% CI). [b]Effect estimates of multiple linear regression are presented as beta coefficient (95% CI). [c]Effect estimates of multiple linear regression for naturally log-transformed outcome variables are presented as means ratio (95% CI). All models were adjusted for age, BMI, pubertal status, siblings, moderate-to-vigorous physical activity, screen time, total difficulties, parental education, parental BMI, study, and recruitment region. Food groups (except water and tea) and nutrients models were further adjusted for total daily energy intake. Water and Tea models were further adjusted for total daily beverage intake. Tertile 1 is the reference category. Significant associations are marked in bold: p<0.0019.

Table S10. Sensitivity analysis (54 females who reported a vegetarian or vegan diet were excluded): Effect estimates and 95% CI assessing the association between tertiles of eating behaviours and dietary intake in females at the 15-year follow-up

		External eating (N=946)				Emotional eating (N=946)				Dietary restraint (N=946)			
		T2 (n=302) Score=6-9		T3 (n=295) Score=10-22		T2 (n=228) Score=3-5		T3 (n=287) Score=6-24		T2 (n=262) Score=3-6		T3 (n=281) Score=7-21	
		Estimate (95% CI)	P-value	Estimate (95% CI)	P-value	Estimate (95% CI)	P-value	Estimate (95% CI)	P-value	Estimate (95% CI)	P-value	Estimate (95% CI)	P-value
**Fruit**[a]	**T2**	1.06 (0.71;1.57)	0.7855	1.12 (0.75;1.67)	0.5701	0.83 (0.55;1.25)	0.3652	0.99 (0.67;1.46)	0.9667	1.01 (0.68;1.51)	0.9510	1.30 (0.86;1.96)	0.2172
	**T3**	1.00 (0.65;1.52)	0.9861	1.02 (0.66;1.57)	0.9209	0.77 (0.49;1.19)	0.2343	1.12 (0.73;1.71)	0.6044	1.55 (1.01;2.39)	0.0461	1.96 (1.26;3.05)	0.0030
**Vegetable**[a]	**T2**	1.24 (0.84;1.84)	0.2826	1.19 (0.80;1.78)	0.3828	0.91 (0.61;1.36)	0.6393	1.16 (0.78;1.71)	0.4668	1.37 (0.93;2.04)	0.1155	1.15 (0.76;1.74)	0.5035
	**T3**	1.03 (0.68;1.56)	0.9021	1.03 (0.68;1.58)	0.8777	0.59 (0.38;0.92)	0.0189	1.24 (0.82;1.87)	0.3194	1.32 (0.86;2.02)	0.2034	1.47 (0.96;2.26)	0.0778
**Starchy**	**T2**	1.24 (0.83;1.85)	0.2844	1.54 (1.04;2.30)	0.0333	1.17 (0.78;1.76)	0.4477	1.07 (0.73;1.58)	0.7340	0.69 (0.46;1.02)	0.0644	0.72 (0.48;1.08)	0.1141
**vegetables**[a]	**T3**	1.35 (0.88;2.05)	0.1669	1.55 (1.01;2.39)	0.0462	1.02 (0.66;1.59)	0.9130	1.23 (0.81;1.86)	0.3338	0.65 (0.42;1.00)	0.0525	0.71 (0.46;1.11)	0.1346
**Whole grains**[a]	**T2**	1.09 (0.74;1.59)	0.6746	1.44 (0.97;2.15)	0.0723	1.09 (0.73;1.63)	0.6741	1.42 (0.96;2.08)	0.0773	1.68 (1.13;2.50)	0.0109	1.30 (0.86;1.95)	0.2082
	**T3**	0.86 (0.58;1.28)	0.4608	1.34 (0.89;2.01)	0.1564	1.07 (0.71;1.60)	0.7591	1.42 (0.95;2.10)	0.0863	1.85 (1.23;2.80)	0.0033	1.79 (1.19;2.71)	0.0057
**Refined grains**[a]	**T2**	1.27 (0.86;1.87)	0.2217	1.14 (0.77;1.71)	0.5094	0.92 (0.61;1.39)	0.6856	0.85 (0.58;1.24)	0.3974	0.83 (0.55;1.24)	0.3556	0.61 (0.41;0.91)	0.0155
	**T3**	1.24 (0.83;1.85)	0.2929	1.63 (1.10;2.43)	0.0156	1.18 (0.79;1.78)	0.4229	0.84 (0.57;1.24)	0.3877	1.13 (0.75;1.68)	0.5648	0.58 (0.39;0.88)	0.0109
**Meat**[a]	**T2**	0.92 (0.62;1.36)	0.6786	0.97 (0.65;1.44)	0.8737	1.33 (0.89;2.01)	0.1655	0.95 (0.64;1.40)	0.7912	0.79 (0.53;1.19)	0.2563	0.84 (0.56;1.27)	0.4096
	**T3**	1.04 (0.70;1.55)	0.8467	1.06 (0.70;1.60)	0.7773	0.90 (0.59;1.38)	0.6392	0.79 (0.53;1.17)	0.2307	1.03 (0.68;1.54)	0.9052	0.75 (0.49;1.15)	0.1830
**Fish**[a]	**T2**	0.69 (0.46;1.02)	0.0612	1.09 (0.73;1.62)	0.6839	1.04 (0.69;1.57)	0.8394	0.94 (0.64;1.37)	0.7355	1.02 (0.69;1.51)	0.9290	0.96 (0.64;1.45)	0.8529
	**T3**	0.93 (0.63;1.37)	0.7214	1.10 (0.74;1.66)	0.6326	1.01 (0.67;1.53)	0.9478	0.84 (0.57;1.24)	0.3908	0.95 (0.63;1.41)	0.7836	1.09 (0.73;1.63)	0.6831
**Eggs**[a]	**T2**	1.06 (0.72;1.56)	0.7834	1.28 (0.87;1.89)	0.2095	1.40 (0.93;2.11)	0.1085	1.44 (0.98;2.10)	0.0601	0.91 (0.62;1.33)	0.6188	0.84 (0.56;1.27)	0.4125
	**T3**	1.10 (0.75;1.62)	0.6314	0.96 (0.64;1.43)	0.8357	1.50 (1.00;2.25)	0.0509	1.10 (0.75;1.63)	0.6279	0.76 (0.51;1.14)	0.1882	1.11 (0.74;1.66)	0.6170
**Nuts and seeds**[a]	**T2**	1.01 (0.69;1.48)	0.9401	0.97 (0.65;1.43)	0.8597	1.11 (0.75;1.66)	0.5931	0.89 (0.61;1.30)	0.5494	1.05 (0.70;1.55)	0.8265	0.98 (0.66;1.46)	0.9399
	**T3**	1.01 (0.68;1.49)	0.9708	1.14 (0.77;1.69)	0.5208	0.88 (0.58;1.33)	0.5463	0.83 (0.56;1.21)	0.3299	1.30 (0.87;1.94)	0.1988	1.04 (0.69;1.57)	0.8455
**Butter**[a]	**T2**	1.25 (0.83;1.88)	0.2846	1.04 (0.69;1.58)	0.8521	1.10 (0.72;1.68)	0.6620	0.78 (0.52;1.18)	0.2375	1.21 (0.80;1.85)	0.3669	0.99 (0.65;1.50)	0.9562
	**T3**	1.59 (1.05;2.41)	0.0289	1.49 (0.98;2.27)	0.0623	0.89 (0.57;1.37)	0.5962	1.00 (0.67;1.49)	0.9868	0.92 (0.60;1.39)	0.6809	0.56 (0.36;0.87)	0.0093
**Margarine**[a]	**T2**	1.21 (0.81;1.83)	0.3557	1.28 (0.86;1.92)	0.2288	1.09 (0.72;1.65)	0.6716	0.93 (0.62;1.38)	0.7137	0.70 (0.46;1.05)	0.0844	0.60 (0.40;0.92)	0.0182
	**T3**	1.22 (0.80;1.85)	0.3578	1.08 (0.70;1.65)	0.7279	0.98 (0.63;1.52)	0.9373	0.94 (0.62;1.43)	0.7780	0.66 (0.43;1.02)	0.0603	0.65 (0.42;1.01)	0.0564
**Oils**[a]	**T2**	0.91 (0.62;1.35)	0.6471	1.13 (0.76;1.67)	0.5436	0.82 (0.54;1.24)	0.3473	1.04 (0.72;1.52)	0.8214	1.06 (0.71;1.57)	0.7882	0.87 (0.58;1.31)	0.5144
	**T3**	0.96 (0.65;1.44)	0.8535	0.89 (0.59;1.35)	0.5921	1.14 (0.76;1.72)	0.5330	0.99 (0.66;1.48)	0.9448	1.13 (0.75;1.70)	0.5707	0.96 (0.63;1.46)	0.8483
**Dairy**[a]	**T2**	1.21 (0.82;1.78)	0.3356	0.98 (0.66;1.45)	0.9083	1.15 (0.77;1.72)	0.4827	1.17 (0.80;1.72)	0.4080	1.02 (0.69;1.50)	0.9244	1.07 (0.71;1.60)	0.7591
	**T3**	0.91 (0.61;1.35)	0.6325	0.80 (0.54;1.18)	0.2602	1.07 (0.71;1.60)	0.7569	0.94 (0.64;1.39)	0.7575	0.92 (0.62;1.38)	0.6929	1.40 (0.93;2.10)	0.1051
**Sugar-sweetend**	**T2**	1.09 (0.74;1.61)	0.6719	1.26 (0.85;1.87)	0.2457	1.05 (0.71;1.57)	0.8021	1.48 (1.00;2.19)	0.0527	0.87 (0.59;1.29)	0.4946	0.59 (0.40;0.89)	0.0113
**food**[a]	**T3**	1.18 (0.79;1.75)	0.4166	1.09 (0.73;1.64)	0.6704	1.14 (0.76;1.73)	0.5224	1.69 (1.13;2.52)	0.0102	0.76 (0.51;1.15)	0.1925	0.65 (0.43;0.97)	0.0374
**Caloric drinks**[a]	**T2**	0.79 (0.54;1.17)	0.2400	1.07 (0.72;1.59)	0.7512	1.02 (0.68;1.52)	0.9393	1.22 (0.83;1.79)	0.3049	0.91 (0.61;1.35)	0.6403	0.87 (0.58;1.30)	0.4830
	**T3**	0.67 (0.45;0.99)	0.0441	0.82 (0.55;1.23)	0.3359	0.81 (0.55;1.22)	0.3155	0.71 (0.48;1.06)	0.0920	0.67 (0.45;1.00)	0.0524	0.70 (0.47;1.05)	0.0846
**Water**[a] **[ml/d]**	**T2**	1.26 (0.85;1.86)	0.2570	1.12 (0.75;1.68)	0.5682	0.98 (0.65;1.47)	0.9083	1.48 (1.00;2.18)	0.0519	0.90 (0.60;1.37)	0.6300	1.36 (0.91;2.04)	0.1323
	**T3**	1.23 (0.76;1.98)	0.3943	1.17 (0.73;1.88)	0.5238	1.23 (0.76;1.99)	0.4038	1.43 (0.89;2.31)	0.1433	1.76 (1.09;2.84)	0.0210	1.54 (0.94;2.53)	0.0865
**Tea**[a] **[ml/d]**	**T2**	1.19 (0.80;1.76)	0.3958	0.76 (0.51;1.13)	0.1757	1.00 (0.65;1.52)	0.9828	0.66 (0.45;0.97)	0.0366	1.08 (0.73;1.60)	0.7000	0.99 (0.65;1.49)	0.9546
	**T3**	1.07 (0.70;1.64)	0.7514	0.93 (0.61;1.41)	0.7327	1.60 (1.03;2.47)	0.0354	0.78 (0.52;1.19)	0.2511	0.87 (0.57;1.34)	0.5273	1.23 (0.81;1.89)	0.3318

Table S10. Sensitivity analysis (54 females who reported a vegetarian or vegan diet were excluded): Effect estimates and 95% CI assessing the association between tertiles of eating behaviours and dietary intake in females at the 15-year follow-up *(Continued)*

	External eating (N=946)				Emotional eating (N=946)				Dietary restraint (N=946)			
	T2 (n=302) Score=6-9		T3 (n=295) Score=10-22		T2 (n=228) Score=3-5		T3 (n=287) Score=6-24		T2 (n=262) Score=3-6		T3 (n=281) Score=7-21	
	Estimate (95% CI)	P-value	Estimate (95% CI)	P-value	Estimate (95% CI)	P-value	Estimate (95% CI)	P-value	Estimate (95% CI)	P-value	Estimate (95% CI)	P-value
**Total energy[b] [kJ/d]**	406 (29;784)	0.0348	710 (329;1,091)	**0.0003**	-100 (-494;293)	0.6166	542 (170;915)	0.0044	-328 (-711;55)	0.0934	-873 (-1,261;-485)	**<0.0001**
**Fat[b]**	0.92 (-0.01;1.86)	0.0536	0.31 (-0.64;1.26)	0.5175	-0.40 (-1.37;0.57)	0.4194	0.31 (-0.62;1.23)	0.5124	-0.40 (-1.35;0.56)	0.4127	-0.20 (-1.17;0.77)	0.6869
**Protein[b]**	-0.14 (-0.58;0.30)	0.5388	-0.16 (-0.61;0.29)	0.4743	-0.26 (-0.72;0.21)	0.2770	-0.36 (-0.80;0.08)	0.1098	-0.13 (-0.58;0.32)	0.5751	0.34 (-0.13;0.80)	0.1533
**Carbohydrate[b]**	-0.75 (-1.89;0.39)	0.1977	-0.10 (-1.26;1.06)	0.8640	0.67 (-0.52;1.86)	0.2692	0.06 (-1.08;1.19)	0.9239	0.55 (-0.61;1.72)	0.3521	-0.16 (-1.35;1.03)	0.7911
**Fibres[b] [g/d]**	-0.41 (-1.24;0.41)	0.3271	0.64 (-0.19;1.48)	0.1318	-0.20 (-1.05;0.66)	0.6522	0.32 (-0.50;1.13)	0.4480	0.90 (0.06;1.74)	0.0354	0.82 (-0.04;1.68)	0.0608
**Total sugar[b]**	-0.74 (-2.01;0.53)	0.2513	-1.19 (-2.48;0.09)	0.0692	0.06 (-1.26;1.38)	0.9268	0.19 (-1.07;1.45)	0.7647	-0.79 (-2.08;0.51)	0.2326	0.51 (-0.81;1.83)	0.4481
**SFA[b]**	0.53 (0.07;0.98)	0.0227	0.24 (-0.22;0.70)	0.3128	-0.28 (-0.75;0.19)	0.2427	0.30 (-0.15;0.75)	0.1874	-0.22 (-0.68;0.24)	0.3524	-0.20 (-0.68;0.27)	0.3985
**MUFA[b]**	0.35 (-0.04;0.74)	0.0805	0.16 (-0.23;0.56)	0.4150	-0.14 (-0.54;0.27)	0.5127	0.08 (-0.30;0.47)	0.6716	-0.03 (-0.43;0.37)	0.8715	-0.02 (-0.43;0.38)	0.9098
**PUFA[c]**	1.01 (0.97;1.05)	0.7567	0.99 (0.95;1.03)	0.4825	1.00 (0.96;1.05)	0.8150	0.99 (0.95;1.03)	0.4643	0.98 (0.94;1.02)	0.2886	1.00 (0.96;1.05)	0.8698
**omega-3 PUFA[c]**	1.03 (0.99;1.07)	0.1053	1.01 (0.98;1.05)	0.4524	0.99 (0.95;1.03)	0.6078	1.00 (0.97;1.04)	0.9244	0.95 (0.92;0.99)	0.0062	0.99 (0.95;1.02)	0.4696
**omega-6 PUFA[c]**	1.00 (0.96;1.05)	0.8744	0.98 (0.94;1.03)	0.4296	1.01 (0.96;1.05)	0.7448	0.98 (0.94;1.03)	0.4451	0.98 (0.94;1.03)	0.4244	1.01 (0.96;1.05)	0.8036

Abbreviations: CI, confidence interval; MUFA, monounsaturated fatty acids; PUFA, polyunsaturated fatty acids; SFA, saturated fatty acids; T2, tertile 2; T3, tertile 3. [a]Effect estimates of multinomial logistic regression are presented as relative risk ratio (95% CI). [b]Effect estimates of multiple linear regression are presented as beta coefficient (95% CI). [c]Effect estimates of multiple linear regression for naturally log-transformed outcome variables are presented as means ratio (95% CI). All models were adjusted for age, BMI, pubertal status, siblings, moderate-to-vigorous physical activity, screen time, total difficulties, parental education, parental BMI, study, and recruitment region. Food groups (except water and tea) and nutrients models were further adjusted for total daily energy intake. Water and Tea models were further adjusted for total daily beverage intake. Tertile 1 is the reference category. Significant associations are marked in bold: $p<0.0019$.

Table S11. Sensitivity analysis (14 males who reported a vegetarian or vegan diet were excluded): Effect estimates and 95% CI assessing the association between tertiles of eating behaviours and dietary intake in males at the 15-year follow-up

		External eating (N=866)				Emotional eating (N=866)				Dietary restraint (N=866)			
		T2 (n=327) Score=6-9		T3 (n=246) Score=10-22		T2 (n=306) Score=3-5		T3 (n=236) Score=6-24		T2 (n=362) Score=3-6		T3 (n=214) Score=7-21	
		Estimate (95% CI)	P-value	Estimate (95% CI)	P-value	Estimate (95% CI)	P-value	Estimate (95% CI)	P-value	Estimate (95% CI)	P-value	Estimate (95% CI)	P-value
**Fruit[a]**	**T2**	1.02 (0.68;1.52)	0.9223	1.09 (0.71;1.68)	0.6961	0.71 (0.48;1.06)	0.0908	0.90 (0.58;1.39)	0.6368	1.18 (0.80;1.74)	0.4068	1.15 (0.71;1.85)	0.5781
	**T3**	1.10 (0.73;1.66)	0.6538	0.91 (0.57;1.44)	0.6799	0.69 (0.46;1.05)	0.0836	0.91 (0.58;1.45)	0.7017	1.30 (0.86;1.97)	0.2123	1.45 (0.87;2.39)	0.1508
**Vegetable[a]**	**T2**	1.02 (0.69;1.51)	0.9167	1.13 (0.74;1.74)	0.5688	0.93 (0.63;1.37)	0.7064	1.09 (0.71;1.67)	0.6925	1.19 (0.81;1.74)	0.3833	1.18 (0.73;1.90)	0.4977
	**T3**	1.06 (0.70;1.60)	0.7809	1.00 (0.64;1.58)	0.9853	0.79 (0.52;1.19)	0.2518	0.92 (0.58;1.45)	0.7207	1.21 (0.80;1.83)	0.3732	1.54 (0.94;2.52)	0.0888
**Starchy**	**T2**	1.06 (0.70;1.59)	0.7813	1.03 (0.67;1.59)	0.8971	1.53 (1.01;2.31)	0.0425	1.00 (0.64;1.55)	0.9956	0.89 (0.60;1.32)	0.5589	0.91 (0.56;1.48)	0.7077
**vegetables[a]**	**T3**	1.11 (0.73;1.70)	0.6200	0.93 (0.59;1.48)	0.7670	1.09 (0.71;1.66)	0.7050	0.72 (0.45;1.13)	0.1556	0.98 (0.64;1.50)	0.9286	1.12 (0.68;1.87)	0.6526
**Whole grains[a]**	**T2**	0.96 (0.65;1.44)	0.8557	0.59 (0.38;0.91)	0.0181	0.76 (0.51;1.12)	0.1652	0.58 (0.37;0.90)	0.0154	0.72 (0.49;1.07)	0.1023	0.96 (0.59;1.56)	0.8772
	**T3**	0.95 (0.63;1.43)	0.7916	0.64 (0.41;1.00)	0.0493	0.74 (0.49;1.11)	0.1490	0.76 (0.49;1.17)	0.2126	0.69 (0.46;1.03)	0.0724	1.10 (0.67;1.79)	0.7066
**Refined grains[a]**	**T2**	0.92 (0.62;1.38)	0.6980	0.77 (0.49;1.20)	0.2477	1.26 (0.84;1.88)	0.2666	0.71 (0.46;1.09)	0.1196	1.23 (0.82;1.84)	0.3220	1.42 (0.88;2.28)	0.1514
	**T3**	0.80 (0.53;1.20)	0.2820	0.95 (0.61;1.48)	0.8196	1.64 (1.09;2.47)	0.0184	1.08 (0.70;1.67)	0.7279	0.93 (0.63;1.39)	0.7309	0.73 (0.45;1.20)	0.2174
**Meat[a]**	**T2**	0.83 (0.55;1.23)	0.3492	0.88 (0.57;1.34)	0.5429	1.01 (0.68;1.51)	0.9450	0.89 (0.58;1.36)	0.5813	0.90 (0.61;1.33)	0.5865	0.94 (0.58;1.53)	0.7925
	**T3**	1.18 (0.79;1.77)	0.4220	1.00 (0.64;1.56)	0.9916	1.02 (0.69;1.53)	0.9067	0.80 (0.52;1.24)	0.3241	0.80 (0.54;1.20)	0.2807	1.09 (0.67;1.76)	0.7344
**Fish[a]**	**T2**	1.21 (0.81;1.79)	0.3501	1.74 (1.12;2.70)	0.0132	0.79 (0.53;1.17)	0.2410	0.97 (0.63;1.52)	0.9081	1.26 (0.85;1.87)	0.2470	1.18 (0.72;1.91)	0.5150
	**T3**	1.55 (1.04;2.30)	0.0299	1.91 (1.22;2.97)	0.0044	0.83 (0.56;1.23)	0.3581	1.33 (0.86;2.06)	0.2012	0.94 (0.63;1.39)	0.7578	1.32 (0.82;2.12)	0.2479
**Eggs[a]**	**T2**	1.49 (1.00;2.23)	0.0485	1.30 (0.84;2.01)	0.2371	1.23 (0.83;1.82)	0.3093	1.32 (0.86;2.04)	0.2096	1.22 (0.83;1.79)	0.3108	1.55 (0.95;2.52)	0.0770
	**T3**	1.33 (0.89;1.99)	0.1673	1.16 (0.75;1.80)	0.5131	1.10 (0.74;1.64)	0.6458	1.25 (0.80;1.94)	0.3246	1.34 (0.89;2.00)	0.1561	1.96 (1.20;3.19)	0.0068
**Nuts and seeds[a]**	**T2**	1.00 (0.68;1.48)	0.9948	1.23 (0.79;1.89)	0.3575	1.22 (0.83;1.80)	0.3090	1.19 (0.77;1.83)	0.4397	1.04 (0.71;1.53)	0.8495	1.60 (0.99;2.59)	0.0564
	**T3**	0.98 (0.66;1.46)	0.9319	1.16 (0.75;1.79)	0.5185	1.39 (0.93;2.08)	0.1036	1.62 (1.05;2.50)	0.0304	1.18 (0.79;1.75)	0.4137	2.02 (1.24;3.30)	0.0047
**Butter[a]**	**T2**	1.49 (0.99;2.26)	0.0589	1.22 (0.78;1.91)	0.3910	0.95 (0.63;1.44)	0.8053	1.01 (0.64;1.59)	0.9621	1.65 (1.09;2.51)	0.0180	1.72 (1.04;2.85)	0.0349
	**T3**	2.01 (1.31;3.09)	**0.0014**	1.35 (0.84;2.15)	0.2144	0.95 (0.62;1.46)	0.8282	0.92 (0.58;1.47)	0.7267	1.17 (0.76;1.78)	0.4765	1.53 (0.92;2.54)	0.1038
**Margarine[a]**	**T2**	1.10 (0.73;1.66)	0.6531	0.94 (0.60;1.47)	0.7984	0.86 (0.56;1.30)	0.4652	1.14 (0.73;1.77)	0.5730	0.96 (0.64;1.45)	0.8498	1.27 (0.78;2.09)	0.3382
	**T3**	1.21 (0.80;1.83)	0.3748	1.05 (0.67;1.65)	0.8323	1.01 (0.67;1.52)	0.9653	0.97 (0.61;1.53)	0.8949	0.90 (0.60;1.36)	0.6270	1.14 (0.69;1.87)	0.6043
**Oils[a]**	**T2**	1.08 (0.72;1.60)	0.7182	1.01 (0.65;1.57)	0.9566	1.13 (0.76;1.68)	0.5408	1.10 (0.72;1.69)	0.6611	0.76 (0.52;1.13)	0.1746	0.59 (0.37;0.96)	0.0328
	**T3**	0.73 (0.48;1.10)	0.1355	0.86 (0.55;1.35)	0.5129	1.12 (0.74;1.68)	0.5893	1.06 (0.68;1.67)	0.7846	0.94 (0.62;1.42)	0.7555	0.92 (0.57;1.51)	0.7506
**Dairy[a]**	**T2**	1.21 (0.82;1.80)	0.3427	0.87 (0.56;1.34)	0.5312	1.61 (1.08;2.39)	0.0193	1.35 (0.88;2.08)	0.1719	1.13 (0.76;1.68)	0.5373	1.19 (0.74;1.91)	0.4747
	**T3**	0.91 (0.60;1.36)	0.6292	0.76 (0.49;1.18)	0.2279	1.06 (0.71;1.58)	0.7756	0.91 (0.59;1.42)	0.6896	0.88 (0.59;1.31)	0.5193	0.95 (0.59;1.53)	0.8232
**Sugar-sweetend**	**T2**	1.42 (0.96;2.10)	0.0777	1.03 (0.66;1.60)	0.9028	1.50 (1.01;2.22)	0.0430	1.34 (0.86;2.08)	0.1925	0.81 (0.55;1.21)	0.3029	0.81 (0.51;1.30)	0.3825
**food[a]**	**T3**	1.11 (0.75;1.66)	0.6026	1.30 (0.85;1.98)	0.2313	1.15 (0.77;1.71)	0.4943	1.44 (0.94;2.22)	0.0930	0.92 (0.62;1.37)	0.6824	0.79 (0.49;1.27)	0.3293
**Caloric drinks[a]**	**T2**	0.96 (0.65;1.42)	0.8436	0.67 (0.43;1.05)	0.0793	1.05 (0.71;1.55)	0.7968	0.72 (0.46;1.11)	0.1404	0.91 (0.61;1.35)	0.6297	0.67 (0.42;1.08)	0.0981
	**T3**	0.99 (0.66;1.48)	0.9548	1.19 (0.78;1.83)	0.4208	0.90 (0.60;1.34)	0.5914	0.91 (0.60;1.40)	0.6789	1.14 (0.77;1.70)	0.5073	0.75 (0.47;1.20)	0.2330
**Water[a] [ml/d]**	**T2**	1.32 (0.88;1.99)	0.1787	0.95 (0.61;1.47)	0.8072	1.07 (0.71;1.60)	0.7519	0.93 (0.60;1.44)	0.7391	1.34 (0.90;2.00)	0.1561	1.92 (1.16;3.17)	0.0109
	**T3**	1.22 (0.77;1.94)	0.3922	0.86 (0.53;1.42)	0.5620	0.90 (0.57;1.42)	0.6413	0.96 (0.58;1.57)	0.8602	0.91 (0.58;1.43)	0.6786	1.79 (1.04;3.10)	0.0360
**Tea[a] [ml/d]**	**T2**	1.71 (1.13;2.59)	0.0112	1.69 (1.08;2.63)	0.0217	1.35 (0.90;2.02)	0.1490	1.43 (0.92;2.24)	0.1136	0.92 (0.61;1.37)	0.6690	1.28 (0.79;2.07)	0.3240
	**T3**	1.50 (0.99;2.28)	0.0564	1.16 (0.73;1.83)	0.5287	1.29 (0.85;1.96)	0.2403	1.55 (0.98;2.45)	0.0592	1.10 (0.73;1.66)	0.6508	1.12 (0.67;1.86)	0.6683

Table S11. Sensitivity analysis (14 males who reported a vegetarian or vegan diet were excluded): Effect estimates and 95% CI assessing the association between tertiles of eating behaviours and dietary intake in males at the 15-year follow-up *(Continued)*

	External eating (N=866)				Emotional eating (N=866)				Dietary restraint (N=866)			
	T2 (n=327) Score=6-9		T3 (n=246) Score=10-22		T2 (n=306) Score=3-5		T3 (n=236) Score=6-24		T2 (n=362) Score=3-6		T3 (n=214) Score=7-21	
	Estimate (95% CI)	P-value	Estimate (95% CI)	P-value	Estimate (95% CI)	P-value	Estimate (95% CI)	P-value	Estimate (95% CI)	P-value	Estimate (95% CI)	P-value
**Total energy[b] [kJ/d]**	232 (-212;676)	0.3053	312 (-173;798)	0.2072	-52 (-494;391)	0.8185	317 (-167;801)	0.1990	-248 (-687;192)	0.2697	-689 (-1,217;-160)	0.0107
**Fat[b]**	0.88 (-0.02;1.78)	0.0567	0.41 (-0.58;1.39)	0.4206	0.13 (-0.77;1.03)	0.7733	0.73 (-0.26;1.71)	0.1483	-0.22 (-1.12;0.67)	0.6258	0.69 (-0.39;1.77)	0.2111
**Protein[b]**	0.04 (-0.41;0.48)	0.8689	-0.44 (-0.92;0.05)	0.0781	-0.29 (-0.74;0.15)	0.1903	-0.34 (-0.83;0.14)	0.1638	-0.07 (-0.51;0.37)	0.7617	0.42 (-0.11;0.95)	0.1241
**Carbohydrate[b]**	-0.88 (-2.01;0.25)	0.1258	0.04 (-1.20;1.28)	0.9476	0.16 (-0.96;1.29)	0.7765	-0.38 (-1.62;0.85)	0.5419	0.24 (-0.88;1.36)	0.6750	-1.13 (-2.48;0.23)	0.1025
**Fibres[b] [g/d]**	0.16 (-0.81;1.13)	0.7428	-0.52 (-1.58;0.54)	0.3390	-0.15 (-1.11;0.81)	0.7623	-1.00 (-2.06;0.05)	0.0629	-0.15 (-1.11;0.81)	0.7529	0.95 (-0.21;2.11)	0.1079
**Total sugar[b]**	-0.48 (-1.75;0.79)	0.4602	0.43 (-0.96;1.82)	0.5420	-0.55 (-1.82;0.72)	0.3935	0.20 (-1.18;1.59)	0.7754	0.79 (-0.47;2.05)	0.2193	-0.58 (-2.10;0.94)	0.4545
**SFA[b]**	0.55 (0.11;1.00)	0.0143	0.28 (-0.21;0.76)	0.2662	0.02 (-0.42;0.46)	0.9295	0.37 (-0.12;0.85)	0.1375	-0.08 (-0.52;0.36)	0.7329	0.26 (-0.27;0.79)	0.3390
**MUFA[b]**	0.30 (-0.09;0.68)	0.1288	0.06 (-0.36;0.48)	0.7741	0.02 (-0.37;0.40)	0.9366	0.13 (-0.29;0.55)	0.5368	-0.05 (-0.43;0.33)	0.8014	0.32 (-0.14;0.78)	0.1675
**PUFA[c]**	1.00 (0.96;1.04)	0.9175	1.01 (0.97;1.05)	0.6753	1.02 (0.98;1.06)	0.2975	1.04 (0.99;1.08)	0.1032	0.98 (0.94;1.02)	0.2949	1.02 (0.97;1.07)	0.4949
**omega-3 PUFA[c]**	1.05 (1.01;1.09)	0.0145	1.07 (1.02;1.11)	**0.0018**	1.01 (0.97;1.05)	0.6393	1.05 (1.01;1.10)	0.0144	0.99 (0.95;1.02)	0.4550	1.01 (0.96;1.05)	0.7733
**omega-6 PUFA[c]**	1.00 (0.96;1.04)	0.8606	1.00 (0.96;1.05)	0.9493	1.02 (0.98;1.07)	0.2854	1.03 (0.99;1.08)	0.1518	0.98 (0.94;1.02)	0.2858	1.02 (0.97;1.07)	0.4918

Abbreviations: CI, confidence interval; MUFA, monounsaturated fatty acids; PUFA, polyunsaturated fatty acids; SFA, saturated fatty acids; T2, tertile 2; T3, tertile 3. [a]Effect estimates of multinomial logistic regression are presented as relative risk ratio (95% CI). [b]Effect estimates of multiple linear regression are presented as beta coefficient (95% CI). [c]Effect estimates of multiple linear regression for naturally log-transformed outcome variables are presented as means ratio (95% CI). All models were adjusted for age, BMI, pubertal status, siblings, moderate-to-vigorous physical activity, screen time, total difficulties, parental education, parental BMI, study, and recruitment region. Food groups (except water and tea) and nutrients models were further adjusted for total daily energy intake. Water and Tea models were further adjusted for total daily beverage intake. Tertile 1 is the reference category. Significant associations are marked in bold: $p<0.0019$.

Table S12. Sensitivity analysis (218 females at the 10-year follow-up with a BMI <10th or >90th percentile were excluded): Effect estimates and 95% CI assessing the association between tertiles of eating behaviours and dietary intake in females at the 10-year follow-up

		External eating (N=864)				Emotional eating (N=864)			
		T2 (n=326) Score=5-8		T3 (n=223) Score=9-24		T2 (n=245) Score=1-2		T3 (n=235) Score=3-18	
		Estimate (95% CI)	P-value	Estimate (95% CI)	P-value	Estimate (95% CI)	P-value	Estimate (95% CI)	P-value
Fruit[a]	T2	0.80 (0.53;1.20)	0.2876	0.75 (0.48;1.18)	0.2193	1.29 (0.85;1.96)	0.2271	0.90 (0.59;1.36)	0.6178
	T3	0.96 (0.64;1.45)	0.8455	0.73 (0.46;1.16)	0.1841	1.48 (0.97;2.26)	0.0725	0.98 (0.64;1.49)	0.9172
Vegetable[a]	T2	1.20 (0.80;1.80)	0.3699	1.03 (0.66;1.59)	0.9050	2.10 (1.38;3.21)	**0.0006**	0.83 (0.55;1.24)	0.3611
	T3	0.99 (0.66;1.48)	0.9518	0.60 (0.38;0.96)	0.0331	1.75 (1.13;2.72)	0.0121	0.77 (0.51;1.18)	0.2309
Starchy	T2	1.13 (0.76;1.67)	0.5420	0.95 (0.61;1.50)	0.8353	0.99 (0.67;1.49)	0.9785	1.00 (0.66;1.52)	0.9931
vegetables[a]	T3	0.81 (0.53;1.24)	0.3288	1.12 (0.70;1.79)	0.6284	0.92 (0.60;1.43)	0.7202	1.18 (0.76;1.83)	0.4615
Whole grains[a]	T2	0.88 (0.59;1.31)	0.5391	0.57 (0.36;0.89)	0.0127	0.79 (0.53;1.18)	0.2429	0.69 (0.45;1.06)	0.0896
	T3	1.17 (0.78;1.74)	0.4577	0.72 (0.46;1.12)	0.1451	0.74 (0.49;1.12)	0.1601	0.92 (0.61;1.38)	0.6736
Refined grains[a]	T2	0.89 (0.61;1.30)	0.5452	1.02 (0.65;1.58)	0.9443	1.05 (0.70;1.55)	0.8272	0.93 (0.62;1.39)	0.7111
	T3	0.96 (0.64;1.43)	0.8335	1.41 (0.90;2.20)	0.1357	1.08 (0.71;1.62)	0.7214	0.99 (0.66;1.50)	0.9730
Meat[a]	T2	1.32 (0.90;1.95)	0.1569	1.11 (0.72;1.73)	0.6309	0.74 (0.49;1.11)	0.1496	1.11 (0.74;1.65)	0.6155
	T3	1.20 (0.81;1.78)	0.3541	1.19 (0.77;1.84)	0.4415	1.18 (0.80;1.75)	0.4072	1.04 (0.69;1.58)	0.8459
Fish[a]	T2	1.04 (0.70;1.53)	0.8612	1.29 (0.83;2.00)	0.2663	1.63 (1.09;2.45)	0.0178	1.31 (0.87;1.98)	0.1906
	T3	0.90 (0.61;1.34)	0.6173	1.09 (0.69;1.70)	0.7132	1.43 (0.95;2.16)	0.0850	1.20 (0.79;1.82)	0.3848
Eggs[a]	T2	1.24 (0.84;1.84)	0.2823	1.02 (0.66;1.58)	0.9358	1.12 (0.74;1.69)	0.5948	1.22 (0.81;1.82)	0.3388
	T3	1.37 (0.92;2.03)	0.1217	1.01 (0.65;1.57)	0.9805	1.58 (1.06;2.37)	0.0249	1.03 (0.68;1.58)	0.8742
Nuts and seeds[a]	T2	0.71 (0.48;1.05)	0.0833	0.76 (0.49;1.17)	0.2126	0.83 (0.56;1.24)	0.3640	0.84 (0.55;1.26)	0.3883
	T3	1.22 (0.82;1.81)	0.3227	1.05 (0.68;1.64)	0.8207	0.88 (0.59;1.31)	0.5336	0.90 (0.60;1.36)	0.6204
Butter[a]	T2	0.95 (0.62;1.45)	0.8138	1.04 (0.64;1.69)	0.8686	1.23 (0.79;1.90)	0.3573	1.31 (0.84;2.05)	0.2319
	T3	0.81 (0.53;1.26)	0.3590	0.98 (0.60;1.61)	0.9307	0.92 (0.58;1.45)	0.7175	1.05 (0.66;1.67)	0.8291
Margarine[a]	T2	1.36 (0.91;2.03)	0.1369	0.80 (0.51;1.27)	0.3518	0.82 (0.54;1.25)	0.3646	1.07 (0.71;1.63)	0.7379
	T3	0.92 (0.59;1.42)	0.6984	1.07 (0.67;1.71)	0.7898	1.22 (0.79;1.89)	0.3781	1.28 (0.82;2.00)	0.2847
Oils[a]	T2	1.23 (0.83;1.82)	0.2978	1.01 (0.64;1.59)	0.9635	1.18 (0.78;1.78)	0.4346	1.17 (0.78;1.77)	0.4527
	T3	1.07 (0.71;1.61)	0.7508	1.06 (0.67;1.67)	0.8069	1.58 (1.04;2.40)	0.0321	1.27 (0.83;1.96)	0.2700
Dairy[a]	T2	0.85 (0.57;1.26)	0.4137	0.98 (0.64;1.53)	0.9450	0.99 (0.66;1.48)	0.9488	1.12 (0.75;1.69)	0.5777
	T3	0.77 (0.52;1.15)	0.2095	0.73 (0.46;1.15)	0.1743	0.75 (0.49;1.12)	0.1593	0.69 (0.45;1.06)	0.0907
Sugar-sweetend	T2	1.61 (1.09;2.39)	0.0177	1.56 (1.01;2.41)	0.0439	1.44 (0.97;2.13)	0.0675	1.28 (0.84;1.94)	0.2485
food[a]	T3	1.79 (1.20;2.65)	0.0040	1.42 (0.91;2.21)	0.1268	0.90 (0.60;1.36)	0.6220	1.44 (0.96;2.16)	0.0799
Caloric drinks[a]	T2	1.22 (0.82;1.80)	0.3272	1.40 (0.89;2.19)	0.1429	1.42 (0.95;2.13)	0.0850	1.05 (0.70;1.60)	0.8060
	T3	0.83 (0.57;1.23)	0.3575	1.06 (0.69;1.65)	0.7854	1.04 (0.69;1.56)	0.8516	1.10 (0.74;1.65)	0.6287
Water[a] [ml/d]	T2	1.41 (0.94;2.10)	0.0960	1.06 (0.68;1.65)	0.8078	0.84 (0.56;1.25)	0.3851	0.97 (0.63;1.47)	0.8699
	T3	1.15 (0.76;1.75)	0.5054	0.83 (0.52;1.34)	0.4483	0.82 (0.53;1.25)	0.3522	0.95 (0.61;1.49)	0.8332
Tea[a] [ml/d]	T2	1.33 (0.90;1.97)	0.1579	1.03 (0.66;1.60)	0.9002	0.71 (0.47;1.07)	0.0982	1.16 (0.76;1.75)	0.4938
	T3	1.03 (0.68;1.56)	0.9070	0.87 (0.54;1.39)	0.5540	0.74 (0.48;1.13)	0.1576	0.84 (0.53;1.31)	0.4357
Total energy[b] [kJ/d]		241 (-79;561)	0.1396	207 (-153;568)	0.2596	42 (-287;372)	0.8012	121 (-214;456)	0.4794
Fat[b]		0.20 (-0.67;1.06)	0.6556	0.32 (-0.65;1.29)	0.5180	0.34 (-0.55;1.22)	0.4517	-0.15 (-1.05;0.75)	0.7376
Protein[b]		-0.09 (-0.47;0.29)	0.6472	-0.05 (-0.47;0.38)	0.8265	-0.03 (-0.42;0.36)	0.8728	-0.18 (-0.58;0.21)	0.3615
Carbohydrate[b]		-0.09 (-1.10;0.93)	0.8661	-0.26 (-1.41;0.89)	0.6557	-0.33 (-1.37;0.72)	0.5392	0.35 (-0.72;1.41)	0.5239
Fibres[b] [g/d]		-0.13 (-0.87;0.62)	0.7402	-0.86 (-1.69;-0.02)	0.0451	0.26 (-0.50;1.02)	0.5053	-0.40 (-1.17;0.38)	0.3173
Total sugar[b]		-0.03 (-1.15;1.10)	0.9637	-0.33 (-1.59;0.94)	0.6131	0.25 (-0.91;1.40)	0.6757	0.58 (-0.60;1.75)	0.3342
SFA[b]		0.05 (-0.38;0.48)	0.8201	0.04 (-0.44;0.53)	0.8661	-0.10 (-0.54;0.35)	0.6677	-0.18 (-0.64;0.27)	0.4238
MUFA[b]		0.14 (-0.23;0.51)	0.4593	0.23 (-0.18;0.65)	0.2670	0.19 (-0.19;0.57)	0.3218	-0.03 (-0.41;0.36)	0.8912
PUFA[c]		1.00 (0.97;1.04)	0.9178	1.01 (0.97;1.06)	0.5391	1.04 (1.00;1.08)	0.0483	1.02 (0.98;1.06)	0.4186
omega-3 PUFA[c]		1.00 (0.97;1.03)	0.7725	1.00 (0.97;1.04)	0.8389	1.04 (1.01;1.07)	0.0144	1.01 (0.98;1.04)	0.4546
omega-6 PUFA[c]		1.00 (0.96;1.04)	0.8801	1.01 (0.97;1.06)	0.5285	1.04 (1.00;1.08)	0.0711	1.02 (0.98;1.06)	0.4251

Abbreviations: CI, confidence interval; MUFA, monounsaturated fatty acids; PUFA, polyunsaturated fatty acids; SFA, saturated fatty acids; T2, tertile 2; T3, tertile 3. [a]Effect estimates of multinomial logistic regression are presented as relative risk ratio (95% CI). [b]Effect estimates of multiple linear regression are presented as beta coefficient (95% CI). [c]Effect estimates of multiple linear regression for naturally log-transformed outcome variables are presented as means ratio (95% CI). All models were adjusted for age, BMI, pubertal status, siblings, moderate-to-vigorous physical activity, screen time, total difficulties, parental education, parental BMI, study, and recruitment region. Food groups (except water and tea) and nutrients models were further adjusted for total daily energy intake. Water and Tea models were further adjusted for total daily beverage intake. Tertile 1 is the reference category. Significant associations are marked in bold: $p<0.0019$.

Table S13. Sensitivity analysis (236 males at the 10-year follow-up with a BMI <10th or >90th percentile were excluded): Effect estimates and 95% CI assessing the association between tertiles of eating behaviours and dietary intake in males at the 10-year follow-up

		External eating (N=939)				Emotional eating (n=939)			
		T2 (n=291) Score=6-9		T3 (n=246) Score=10-24		T2 (n=269) Score=1-2		T3 (n=282) Score=3-24	
		Estimate (95% CI)	P-value	Estimate (95% CI)	P-value	Estimate (95% CI)	P-value	Estimate (95% CI)	P-value
Fruit[a]	T2	0.86 (0.59;1.26)	0.4447	0.76 (0.51;1.14)	0.1817	0.97 (0.66;1.43)	0.8842	0.97 (0.65;1.44)	0.8746
	T3	0.98 (0.66;1.44)	0.8999	0.77 (0.50;1.16)	0.2119	0.85 (0.56;1.27)	0.4194	1.09 (0.73;1.63)	0.6762
Vegetable[a]	T2	1.36 (0.93;1.98)	0.1153	0.96 (0.65;1.44)	0.8564	0.82 (0.56;1.21)	0.3214	0.94 (0.63;1.38)	0.7414
	T3	1.09 (0.73;1.63)	0.6589	0.94 (0.62;1.41)	0.7526	0.94 (0.63;1.40)	0.7512	1.00 (0.66;1.51)	0.9997
Starchy vegetables[a]	T2	0.78 (0.53;1.15)	0.2088	0.92 (0.62;1.38)	0.6909	0.86 (0.58;1.28)	0.4672	0.75 (0.51;1.11)	0.1531
	T3	0.81 (0.53;1.24)	0.3322	0.95 (0.60;1.48)	0.8135	0.97 (0.63;1.50)	0.8841	0.77 (0.50;1.20)	0.2541
Whole grains[a]	T2	1.32 (0.90;1.95)	0.1528	1.13 (0.75;1.69)	0.5672	0.96 (0.64;1.42)	0.8210	1.12 (0.75;1.66)	0.5857
	T3	1.14 (0.77;1.69)	0.5043	0.88 (0.58;1.33)	0.5469	1.11 (0.75;1.65)	0.6035	1.05 (0.70;1.58)	0.8043
Refined grains[a]	T2	1.04 (0.71;1.51)	0.8496	1.17 (0.78;1.75)	0.4423	0.98 (0.66;1.46)	0.9375	1.03 (0.70;1.52)	0.8678
	T3	0.89 (0.60;1.31)	0.5440	1.02 (0.68;1.55)	0.9083	0.95 (0.64;1.41)	0.8017	0.70 (0.46;1.05)	0.0815
Meat[a]	T2	1.00 (0.68;1.45)	0.9849	0.87 (0.58;1.29)	0.4817	0.95 (0.65;1.40)	0.7988	0.92 (0.62;1.35)	0.6576
	T3	1.20 (0.82;1.76)	0.3373	1.08 (0.72;1.61)	0.7141	1.00 (0.68;1.48)	0.9963	1.11 (0.75;1.64)	0.6068
Fish[a]	T2	0.92 (0.63;1.33)	0.6455	1.10 (0.73;1.66)	0.6362	0.67 (0.45;0.99)	0.0430	0.93 (0.63;1.38)	0.7292
	T3	0.88 (0.60;1.28)	0.4999	1.39 (0.93;2.08)	0.1039	0.81 (0.55;1.20)	0.2923	1.05 (0.70;1.55)	0.8211
Eggs[a]	T2	0.81 (0.55;1.19)	0.2762	0.74 (0.50;1.11)	0.1502	1.19 (0.79;1.79)	0.3990	0.68 (0.46;1.00)	0.0525
	T3	0.80 (0.54;1.17)	0.2494	0.73 (0.49;1.10)	0.1346	1.84 (1.23;2.76)	0.0031	0.87 (0.58;1.29)	0.4878
Nuts and seeds[a]	T2	1.01 (0.69;1.46)	0.9770	1.01 (0.68;1.52)	0.9558	1.08 (0.73;1.59)	0.7134	0.73 (0.49;1.09)	0.1205
	T3	0.83 (0.56;1.21)	0.3216	0.95 (0.63;1.41)	0.7828	1.29 (0.87;1.92)	0.2033	1.12 (0.76;1.65)	0.5647
Butter[a]	T2	1.26 (0.84;1.90)	0.2696	1.17 (0.75;1.83)	0.4868	1.73 (1.12;2.68)	0.0135	1.15 (0.75;1.77)	0.5207
	T3	1.16 (0.76;1.79)	0.4870	1.22 (0.77;1.93)	0.3936	1.62 (1.04;2.54)	0.0343	0.95 (0.61;1.49)	0.8198
Margarine[a]	T2	0.75 (0.50;1.11)	0.1539	1.05 (0.70;1.57)	0.8106	0.80 (0.53;1.20)	0.2822	1.20 (0.80;1.78)	0.3789
	T3	0.83 (0.55;1.25)	0.3795	0.64 (0.41;1.01)	0.0539	1.06 (0.70;1.62)	0.7762	1.07 (0.70;1.65)	0.7522
Oils[a]	T2	1.15 (0.78;1.70)	0.4688	1.01 (0.66;1.53)	0.9754	0.85 (0.57;1.27)	0.4193	1.34 (0.89;2.02)	0.1581
	T3	1.42 (0.95;2.13)	0.0874	1.53 (1.00;2.34)	0.0484	0.94 (0.62;1.41)	0.7533	1.37 (0.90;2.09)	0.1439
Dairy[a]	T2	0.76 (0.52;1.11)	0.1543	0.61 (0.40;0.91)	0.0147	0.67 (0.45;0.99)	0.0457	0.74 (0.50;1.09)	0.1279
	T3	0.91 (0.61;1.35)	0.6292	0.76 (0.50;1.15)	0.1903	0.82 (0.55;1.23)	0.3425	0.97 (0.64;1.46)	0.8769
Sugar-sweetend food[a]	T2	1.40 (0.96;2.04)	0.0823	0.96 (0.64;1.46)	0.8573	1.29 (0.87;1.92)	0.2034	1.09 (0.74;1.62)	0.6623
	T3	1.12 (0.76;1.65)	0.5742	1.33 (0.89;1.98)	0.1610	1.37 (0.92;2.04)	0.1198	1.21 (0.81;1.79)	0.3514
Caloric drinks[a]	T2	0.83 (0.56;1.22)	0.3361	1.01 (0.68;1.51)	0.9543	0.91 (0.61;1.35)	0.6370	0.76 (0.51;1.13)	0.1736
	T3	1.21 (0.83;1.77)	0.3181	0.95 (0.63;1.43)	0.7931	1.07 (0.72;1.59)	0.7399	0.81 (0.55;1.21)	0.3033
Water[a] [ml/d]	T2	1.09 (0.74;1.61)	0.6666	1.21 (0.80;1.83)	0.3615	0.79 (0.54;1.18)	0.2534	0.73 (0.48;1.09)	0.1237
	T3	0.90 (0.59;1.37)	0.6247	1.07 (0.68;1.69)	0.7557	0.78 (0.50;1.21)	0.2651	1.11 (0.72;1.72)	0.6222
Tea[a] [ml/d]	T2	1.03 (0.69;1.53)	0.9002	1.19 (0.78;1.84)	0.4220	0.96 (0.63;1.45)	0.8291	1.11 (0.73;1.68)	0.6257
	T3	0.94 (0.62;1.42)	0.7596	1.15 (0.74;1.80)	0.5349	1.34 (0.87;2.04)	0.1800	1.10 (0.71;1.70)	0.6745
Total energy[b] [kJ/d]		149 (-218;516)	0.4249	210 (-178;599)	0.2882	368 (-9;744)	0.0555	330 (-48;708)	0.0873
Fat[b]		0.66 (-0.17;1.50)	0.1183	0.85 (-0.03;1.73)	0.0595	0.47 (-0.39;1.33)	0.2802	0.77 (-0.10;1.63)	0.0822
Protein[b]		-0.14 (-0.52;0.23)	0.4513	-0.17 (-0.56;0.23)	0.4050	-0.14 (-0.52;0.25)	0.4860	0.18 (-0.21;0.56)	0.3664
Carbohydrate[b]		-0.52 (-1.52;0.48)	0.3081	-0.66 (-1.72;0.40)	0.2210	-0.33 (-1.36;0.70)	0.5325	-0.94 (-1.97;0.10)	0.0754
Fibres[b] [g/d]		-0.18 (-0.97;0.61)	0.6501	-0.33 (-1.17;0.50)	0.4337	0.23 (-0.58;1.04)	0.5803	0.01 (-0.81;0.83)	0.9802
Total sugar[b]		0.25 (-0.84;1.34)	0.6509	-0.42 (-1.58;0.73)	0.4705	-0.23 (-1.35;0.89)	0.6907	-0.26 (-1.38;0.87)	0.6565
SFA[b]		0.32 (-0.09;0.74)	0.1277	0.36 (-0.08;0.80)	0.1117	0.15 (-0.28;0.58)	0.4940	0.20 (-0.22;0.63)	0.3492
MUFA[b]		0.27 (-0.09;0.62)	0.1385	0.29 (-0.08;0.66)	0.1297	0.20 (-0.16;0.57)	0.2729	0.33 (-0.03;0.70)	0.0734
PUFA[c]		1.01 (0.98;1.05)	0.4649	1.03 (0.99;1.07)	0.1674	1.02 (0.98;1.06)	0.3038	1.04 (1.00;1.08)	0.0541
omega-3 PUFA[c]		1.00 (0.96;1.03)	0.8248	1.03 (0.99;1.06)	0.1331	0.99 (0.96;1.03)	0.7355	1.03 (1.00;1.07)	0.0665
omega-6 PUFA[c]		1.02 (0.98;1.06)	0.4016	1.03 (0.99;1.07)	0.1941	1.02 (0.98;1.07)	0.2494	1.04 (1.00;1.08)	0.0668

Abbreviations: CI, confidence interval; MUFA, monounsaturated fatty acids; PUFA, polyunsaturated fatty acids; SFA, saturated fatty acids; T2, tertile 2; T3, tertile 3. [a]Effect estimates of multinomial logistic regression are presented as relative risk ratio (95% CI). [b]Effect estimates of multiple linear regression are presented as beta coefficient (95% CI). [c]Effect estimates of multiple linear regression for naturally log-transformed outcome variables are presented as means ratio (95% CI). All models were adjusted for age, BMI, pubertal status, siblings, moderate-to-vigorous physical activity, screen time, total difficulties, parental education, parental BMI, study, and recruitment region. Food groups (except water and tea) and nutrients models were further adjusted for total daily energy intake. Water and Tea models were further adjusted for total daily beverage intake. Tertile 1 is the reference category. Significant associations are marked in bold: $p<0.0019$.

Table S14. Sensitivity analysis (200 females at the 15-year follow-up with a BMI <10th or >90th percentile were excluded): Effect estimates and 95% CI assessing the association between tertiles of eating behaviours and dietary intake in females at the 15-year follow-up

		External eating (N=800)				Emotional eating (N=800)				Dietary restraint (N=800)			
		T2 (n=258) Score=6-9		T3 (n=255) Score=10-22		T2 (n=191) Score=3-5		T3 (n=239) Score=6-24		T2 (n=230) Score=3-6		T3 (n=251) Score=7-21	
		Estimate (95% CI)	P-value	Estimate (95% CI)	P-value	Estimate (95% CI)	P-value	Estimate (95% CI)	P-value	Estimate (95% CI)	P-value	Estimate (95% CI)	P-value
Fruit[a]	T2	1.18 (0.76;1.83)	0.4602	1.40 (0.90;2.20)	0.1373	0.80 (0.51;1.27)	0.3474	1.20 (0.77;1.88)	0.4155	0.90 (0.58;1.40)	0.6348	1.25 (0.79;1.97)	0.3403
	T3	1.23 (0.77;1.95)	0.3925	1.33 (0.82;2.16)	0.2414	0.74 (0.46;1.20)	0.2277	1.32 (0.82;2.12)	0.2575	1.71 (1.06;2.74)	0.0270	1.98 (1.22;3.24)	0.0062
Vegetable[a]	T2	1.40 (0.90;2.16)	0.1356	1.35 (0.86;2.11)	0.1949	0.86 (0.55;1.35)	0.5250	1.12 (0.72;1.74)	0.6174	1.45 (0.94;2.26)	0.0966	1.02 (0.64;1.61)	0.9341
	T3	1.12 (0.71;1.77)	0.6349	1.28 (0.80;2.03)	0.3029	0.67 (0.41;1.07)	0.0945	1.30 (0.82;2.06)	0.2668	1.40 (0.88;2.24)	0.1602	1.39 (0.87;2.21)	0.1690
Starchy	T2	1.52 (0.99;2.33)	0.0544	1.72 (1.11;2.67)	0.0154	1.34 (0.86;2.07)	0.1922	1.24 (0.81;1.90)	0.3248	0.75 (0.49;1.16)	0.2025	0.82 (0.53;1.27)	0.3728
vegetables[a]	T3	1.41 (0.88;2.25)	0.1511	1.73 (1.08;2.80)	0.0236	1.12 (0.69;1.82)	0.6567	1.36 (0.85;2.16)	0.1984	0.76 (0.47;1.22)	0.2487	0.74 (0.45;1.20)	0.2195
Whole grains[a]	T2	1.21 (0.79;1.86)	0.3827	1.37 (0.87;2.14)	0.1697	1.04 (0.67;1.61)	0.8781	1.31 (0.84;2.04)	0.2333	1.70 (1.09;2.65)	0.0194	1.44 (0.92;2.26)	0.1135
	T3	0.94 (0.61;1.46)	0.7958	1.25 (0.80;1.95)	0.3320	0.88 (0.56;1.39)	0.5936	1.46 (0.94;2.27)	0.0912	2.05 (1.31;3.23)	**0.0018**	2.02 (1.28;3.19)	0.0025
Refined grains[a]	T2	1.37 (0.90;2.09)	0.1471	1.17 (0.75;1.81)	0.4945	0.94 (0.60;1.47)	0.7725	0.84 (0.55;1.28)	0.4119	0.88 (0.56;1.38)	0.5832	0.61 (0.39;0.95)	0.0283
	T3	1.19 (0.77;1.85)	0.4368	1.66 (1.07;2.57)	0.0235	1.28 (0.82;2.00)	0.2777	0.86 (0.55;1.33)	0.4930	1.19 (0.77;1.86)	0.4315	0.52 (0.33;0.82)	0.0046
Meat[a]	T2	1.13 (0.74;1.73)	0.5765	1.11 (0.72;1.71)	0.6519	1.42 (0.91;2.22)	0.1186	0.83 (0.54;1.27)	0.3973	0.72 (0.46;1.11)	0.1385	0.87 (0.56;1.34)	0.5267
	T3	1.18 (0.76;1.83)	0.4613	1.22 (0.78;1.90)	0.3925	1.06 (0.67;1.68)	0.8121	0.72 (0.47;1.12)	0.1417	0.98 (0.63;1.53)	0.9352	0.74 (0.47;1.18)	0.2108
Fish[a]	T2	0.75 (0.49;1.14)	0.1777	1.08 (0.70;1.67)	0.7261	1.30 (0.83;2.03)	0.2519	0.99 (0.65;1.50)	0.9540	0.90 (0.59;1.39)	0.6440	0.98 (0.63;1.53)	0.9313
	T3	0.98 (0.64;1.50)	0.9165	1.18 (0.76;1.85)	0.4618	1.16 (0.74;1.82)	0.5068	0.77 (0.50;1.20)	0.2498	0.91 (0.58;1.41)	0.6608	1.14 (0.73;1.78)	0.5553
Eggs[a]	T2	1.00 (0.65;1.55)	0.9830	1.30 (0.84;2.00)	0.2362	1.42 (0.90;2.22)	0.1310	1.34 (0.88;2.04)	0.1776	1.06 (0.69;1.63)	0.7847	0.89 (0.57;1.40)	0.6139
	T3	1.21 (0.80;1.83)	0.3736	1.07 (0.69;1.65)	0.7728	1.65 (1.06;2.55)	0.0256	1.16 (0.76;1.77)	0.4968	0.85 (0.55;1.32)	0.4707	1.17 (0.76;1.80)	0.4806
Nuts and seeds[a]	T2	0.89 (0.58;1.37)	0.6092	0.89 (0.58;1.38)	0.6087	1.17 (0.75;1.82)	0.4946	0.96 (0.62;1.47)	0.8417	1.00 (0.64;1.55)	0.9869	1.15 (0.74;1.78)	0.5452
	T3	0.98 (0.64;1.50)	0.9160	0.90 (0.58;1.40)	0.6557	0.89 (0.57;1.39)	0.6068	0.65 (0.42;1.01)	0.0557	1.16 (0.75;1.80)	0.4921	1.00 (0.64;1.56)	0.9937
Butter[a]	T2	1.34 (0.85;2.11)	0.2034	1.11 (0.70;1.76)	0.6491	1.21 (0.76;1.92)	0.4307	1.03 (0.65;1.63)	0.8945	0.97 (0.61;1.55)	0.9075	0.91 (0.57;1.46)	0.7022
	T3	1.52 (0.96;2.40)	0.0710	1.33 (0.84;2.12)	0.2237	0.82 (0.51;1.33)	0.4221	0.98 (0.62;1.53)	0.9159	0.84 (0.53;1.33)	0.4545	0.61 (0.38;0.98)	0.0431
Margarine[a]	T2	1.18 (0.75;1.84)	0.4688	1.34 (0.86;2.08)	0.1991	1.05 (0.67;1.65)	0.8237	1.05 (0.68;1.63)	0.8298	0.62 (0.39;0.97)	0.0374	0.68 (0.43;1.08)	0.0995
	T3	1.22 (0.77;1.94)	0.3912	1.10 (0.68;1.77)	0.6998	0.82 (0.50;1.32)	0.4082	0.88 (0.56;1.40)	0.5994	0.54 (0.34;0.87)	0.0116	0.64 (0.39;1.04)	0.0698
Oils[a]	T2	0.95 (0.61;1.47)	0.8090	1.09 (0.70;1.68)	0.7149	0.80 (0.51;1.27)	0.3468	0.96 (0.62;1.47)	0.8453	1.01 (0.65;1.57)	0.9732	0.72 (0.46;1.12)	0.1469
	T3	0.97 (0.63;1.50)	0.8862	0.87 (0.55;1.36)	0.5324	1.04 (0.67;1.63)	0.8526	0.87 (0.55;1.35)	0.5274	1.16 (0.74;1.83)	0.5125	1.00 (0.64;1.57)	0.9991
Dairy[a]	T2	1.05 (0.68;1.61)	0.8205	0.91 (0.59;1.40)	0.6588	1.13 (0.73;1.75)	0.5885	1.19 (0.78;1.83)	0.4201	1.00 (0.65;1.53)	0.9894	1.07 (0.68;1.68)	0.7634
	T3	0.91 (0.59;1.41)	0.6731	0.74 (0.48;1.16)	0.1888	1.13 (0.72;1.77)	0.5889	1.02 (0.66;1.58)	0.9140	0.98 (0.63;1.53)	0.9260	1.46 (0.93;2.28)	0.1009
Sugar-sweetend	T2	0.93 (0.60;1.43)	0.7374	1.51 (0.97;2.33)	0.0661	0.99 (0.64;1.53)	0.9622	1.43 (0.92;2.22)	0.1078	0.85 (0.55;1.31)	0.4507	0.67 (0.43;1.05)	0.0823
food[a]	T3	1.08 (0.71;1.64)	0.7225	1.12 (0.72;1.73)	0.6241	1.10 (0.71;1.70)	0.6816	1.60 (1.04;2.47)	0.0327	0.74 (0.48;1.15)	0.1773	0.67 (0.43;1.04)	0.0757
Caloric drinks[a]	T2	0.81 (0.52;1.24)	0.3254	1.13 (0.73;1.75)	0.5929	1.06 (0.68;1.66)	0.7833	1.28 (0.84;1.96)	0.2534	0.98 (0.63;1.51)	0.9093	0.95 (0.61;1.49)	0.8357
	T3	0.63 (0.41;0.97)	0.0358	0.78 (0.50;1.22)	0.2830	0.80 (0.52;1.24)	0.3234	0.59 (0.38;0.92)	0.0198	0.62 (0.40;0.97)	0.0358	0.67 (0.43;1.04)	0.0746
Water[a] [ml/d]	T2	1.20 (0.78;1.85)	0.4184	1.20 (0.77;1.86)	0.4297	1.37 (0.87;2.16)	0.1733	2.13 (1.37;3.33)	**0.0009**	1.08 (0.68;1.70)	0.7437	1.80 (1.15;2.80)	0.0099
	T3	1.13 (0.68;1.90)	0.6329	1.00 (0.59;1.68)	0.9964	1.28 (0.75;2.17)	0.3658	1.51 (0.88;2.59)	0.1340	1.80 (1.07;3.03)	0.0278	1.70 (0.98;2.94)	0.0587
Tea[a] [ml/d]	T2	1.29 (0.83;1.99)	0.2536	0.88 (0.57;1.37)	0.5807	1.26 (0.79;2.02)	0.3372	0.77 (0.50;1.18)	0.2235	1.04 (0.67;1.61)	0.8728	1.09 (0.70;1.72)	0.7003
	T3	1.27 (0.80;2.03)	0.3133	1.05 (0.66;1.66)	0.8323	1.89 (1.16;3.07)	0.0104	0.75 (0.47;1.19)	0.2190	0.95 (0.59;1.52)	0.8306	1.26 (0.79;2.02)	0.3333

Table S14. Sensitivity analysis (200 females at the 15-year follow-up with a BMI <10th or >90th percentile were excluded): Effect estimates and 95% CI assessing the association between tertiles of eating behaviours and dietary intake in females at the 15-year follow-up *(Continued)*

	External eating (N=800)				Emotional eating (N=800)				Dietary restraint (N=800)			
	T2 (n=258) Score=6-9		T3 (n=255) Score=10-22		T2 (n=191) Score=3-5		T3 (n=239) Score=6-24		T2 (n=230) Score=3-6		T3 (n=251) Score=7-21	
	**Estimate (95% CI)**	**P-value**	**Estimate (95% CI)**	**P-value**	**Estimate (95% CI)**	**P-value**	**Estimate (95% CI)**	**P-value**	**Estimate (95% CI)**	**P-value**	**Estimate (95% CI)**	**P-value**
**Total energy[b] [kJ/d]**	554 (139;968)	0.0089	897 (476;1,318)	**<0.0001**	74 (-360;507)	0.7385	595 (177;1,013)	0.0053	-382 (-805;41)	0.0768	-965 (-1,391;-538)	**<0.0001**
**Fat[b]**	0.99 (-0.04;2.03)	0.0605	0.08 (-0.98;1.14)	0.8804	-0.19 (-1.26;0.88)	0.7299	-0.13 (-1.17;0.91)	0.8053	-0.58 (-1.63;0.48)	0.2845	-0.09 (-1.17;0.99)	0.8698
**Protein[b]**	-0.03 (-0.50;0.43)	0.8878	-0.25 (-0.73;0.22)	0.2971	-0.03 (-0.51;0.45)	0.9093	-0.40 (-0.86;0.07)	0.0967	0.01 (-0.47;0.48)	0.9801	0.24 (-0.25;0.72)	0.3392
**Carbohydrate[b]**	-0.92 (-2.16;0.33)	0.1489	0.21 (-1.06;1.48)	0.7480	0.25 (-1.04;1.54)	0.7057	0.53 (-0.73;1.78)	0.4099	0.60 (-0.68;1.87)	0.3573	-0.17 (-1.47;1.12)	0.7910
**Fibres[b] [g/d]**	-0.23 (-1.17;0.71)	0.6304	0.86 (-0.10;1.82)	0.0777	-0.27 (-1.24;0.70)	0.5883	0.44 (-0.50;1.38)	0.3595	0.81 (-0.14;1.77)	0.0955	0.42 (-0.55;1.40)	0.3955
**Total sugar[b]**	-0.98 (-2.38;0.41)	0.1660	-0.97 (-2.39;0.46)	0.1832	-0.37 (-1.81;1.07)	0.6141	0.47 (-0.93;1.87)	0.5091	-0.80 (-2.21;0.62)	0.2700	0.83 (-0.62;2.27)	0.2611
**SFA[b]**	0.50 (0.00;1.01)	0.0479	0.10 (-0.41;0.61)	0.7122	-0.21 (-0.72;0.31)	0.4354	0.13 (-0.37;0.64)	0.6001	-0.33 (-0.84;0.18)	0.2035	-0.08 (-0.60;0.44)	0.7573
**MUFA[b]**	0.42 (-0.01;0.86)	0.0556	0.07 (-0.38;0.51)	0.7715	0.01 (-0.44;0.46)	0.9640	-0.09 (-0.53;0.35)	0.6838	-0.10 (-0.54;0.35)	0.6668	-0.03 (-0.48;0.42)	0.8962
**PUFA[c]**	1.01 (0.97;1.06)	0.6520	0.99 (0.94;1.03)	0.5894	1.00 (0.95;1.04)	0.9223	0.97 (0.92;1.01)	0.1333	0.98 (0.93;1.02)	0.3363	1.00 (0.95;1.05)	0.9770
**omega-3 PUFA[c]**	1.02 (0.98;1.06)	0.2515	1.02 (0.98;1.06)	0.4472	0.99 (0.95;1.04)	0.7784	0.99 (0.95;1.03)	0.4796	0.94 (0.90;0.98)	0.0028	0.98 (0.94;1.02)	0.3211
**omega-6 PUFA[c]**	1.01 (0.96;1.06)	0.7206	0.98 (0.94;1.03)	0.5321	1.00 (0.95;1.05)	0.9426	0.96 (0.92;1.01)	0.1353	0.98 (0.94;1.03)	0.5001	1.00 (0.95;1.05)	0.9549

Abbreviations: CI, confidence interval; MUFA, monounsaturated fatty acids; PUFA, polyunsaturated fatty acids; SFA, saturated fatty acids; T2, tertile 2; T3, tertile 3. [a]Effect estimates of multinomial logistic regression are presented as relative risk ratio (95% CI). [b]Effect estimates of multiple linear regression are presented as beta coefficient (95% CI). [c]Effect estimates of multiple linear regression for naturally log-transformed outcome variables are presented as means ratio (95% CI). All models were adjusted for age, BMI, pubertal status, siblings, moderate-to-vigorous physical activity, screen time, total difficulties, parental education, parental BMI, study, and recruitment region. Food groups (except water and tea) and nutrients models were further adjusted for total daily energy intake. Water and Tea models were further adjusted for total daily beverage intake. Tertile 1 is the reference category. Significant associations are marked in bold: p<0.0019.

Table S15. Sensitivity analysis (176 males at the 15-year follow-up with a BMI <10th or >90th percentile were excluded): Effect estimates and 95% CI assessing the association between tertiles of eating behaviours and dietary intake in males at the 15-year follow-up

		External eating (N=704)				Emotional eating (N=704)				Dietary restraint (N=704)			
		T2 (n=269) Score=5-9		T3 (n=197) Score=10-22		T2 (n=249) Score=1-3		T3 (n=182) Score=4-23		T2 (n=309) Score=1-3		T3 (n=173) Score=4-21	
		**Estimate (95% CI)**	**P-value**	**Estimate (95% CI)**	**P-value**	**Estimate (95% CI)**	**P-value**	**Estimate (95% CI)**	**P-value**	**Estimate (95% CI)**	**P-value**	**Estimate (95% CI)**	**P-value**
**Fruit[a]**	**T2**	1.05 (0.67;1.65)	0.8147	1.08 (0.66;1.75)	0.7581	0.62 (0.40;0.97)	0.0379	0.89 (0.55;1.45)	0.6428	1.33 (0.86;2.06)	0.2045	1.29 (0.76;2.22)	0.3465
	**T3**	1.08 (0.68;1.71)	0.7494	0.84 (0.50;1.40)	0.5027	0.71 (0.45;1.12)	0.1417	0.88 (0.52;1.47)	0.6194	1.49 (0.93;2.37)	0.0956	1.58 (0.90;2.76)	0.1120
**Vegetable[a]**	**T2**	1.16 (0.75;1.80)	0.5016	1.28 (0.79;2.08)	0.3191	0.87 (0.56;1.35)	0.5326	1.31 (0.81;2.13)	0.2763	1.22 (0.79;1.87)	0.3685	1.24 (0.73;2.13)	0.4239
	**T3**	1.04 (0.65;1.65)	0.8725	0.99 (0.59;1.65)	0.9623	0.80 (0.50;1.26)	0.3336	1.12 (0.66;1.90)	0.6679	1.36 (0.85;2.17)	0.2028	1.68 (0.96;2.95)	0.0686
**Starchy vegetables[a]**	**T2**	1.05 (0.67;1.66)	0.8205	1.01 (0.62;1.65)	0.9616	1.29 (0.82;2.03)	0.2718	1.13 (0.69;1.84)	0.6229	0.81 (0.52;1.27)	0.3648	0.91 (0.53;1.57)	0.7383
	**T3**	1.12 (0.71;1.79)	0.6197	0.81 (0.49;1.35)	0.4222	1.07 (0.68;1.70)	0.7694	0.70 (0.42;1.16)	0.1665	0.99 (0.62;1.59)	0.9825	1.14 (0.65;1.99)	0.6510
**Whole grains[a]**	**T2**	1.02 (0.66;1.58)	0.9336	0.54 (0.33;0.89)	0.0149	0.84 (0.54;1.29)	0.4203	0.64 (0.39;1.04)	0.0692	0.96 (0.62;1.49)	0.8478	1.26 (0.73;2.15)	0.4036
	**T3**	1.08 (0.68;1.71)	0.7375	0.67 (0.41;1.10)	0.1097	0.93 (0.59;1.45)	0.7360	0.80 (0.49;1.31)	0.3807	0.77 (0.49;1.22)	0.2649	1.12 (0.65;1.93)	0.6908
**Refined grains[a]**	**T2**	0.93 (0.60;1.45)	0.7571	0.81 (0.49;1.33)	0.3976	1.20 (0.77;1.87)	0.4196	0.70 (0.43;1.14)	0.1560	1.31 (0.83;2.06)	0.2397	1.39 (0.82;2.35)	0.2249
	**T3**	0.86 (0.55;1.35)	0.5084	1.03 (0.63;1.68)	0.9209	1.87 (1.19;2.95)	0.0071	1.29 (0.79;2.11)	0.3084	1.05 (0.68;1.64)	0.8177	0.83 (0.48;1.43)	0.4969
**Meat[a]**	**T2**	0.99 (0.64;1.53)	0.9468	1.05 (0.65;1.69)	0.8441	0.96 (0.62;1.47)	0.8391	0.92 (0.57;1.49)	0.7338	0.94 (0.61;1.45)	0.7838	1.07 (0.63;1.81)	0.8047
	**T3**	1.15 (0.74;1.80)	0.5305	1.09 (0.66;1.80)	0.7366	0.89 (0.57;1.39)	0.6046	1.00 (0.61;1.64)	0.9881	0.86 (0.55;1.34)	0.4973	1.16 (0.67;1.99)	0.5950
**Fish[a]**	**T2**	1.03 (0.66;1.59)	0.9027	1.71 (1.05;2.78)	0.0324	0.82 (0.53;1.26)	0.3651	1.10 (0.67;1.82)	0.7008	1.39 (0.89;2.16)	0.1477	1.41 (0.82;2.42)	0.2141
	**T3**	1.39 (0.90;2.15)	0.1337	1.87 (1.14;3.06)	0.0135	0.77 (0.50;1.19)	0.2391	1.62 (1.00;2.64)	0.0520	0.96 (0.62;1.49)	0.8720	1.39 (0.82;2.35)	0.2175
**Eggs[a]**	**T2**	1.49 (0.95;2.32)	0.0816	1.18 (0.73;1.93)	0.4990	1.11 (0.72;1.72)	0.6368	1.55 (0.94;2.55)	0.0833	1.46 (0.94;2.25)	0.0907	1.49 (0.87;2.57)	0.1463
	**T3**	1.41 (0.90;2.22)	0.1359	1.09 (0.67;1.78)	0.7364	0.96 (0.61;1.50)	0.8467	1.45 (0.88;2.40)	0.1439	1.52 (0.97;2.39)	0.0682	1.92 (1.12;3.30)	0.0181
**Nuts and seeds[a]**	**T2**	1.03 (0.67;1.60)	0.8832	1.45 (0.89;2.36)	0.1379	1.26 (0.82;1.93)	0.2973	1.30 (0.79;2.14)	0.3105	1.17 (0.76;1.81)	0.4715	1.82 (1.05;3.13)	0.0314
	**T3**	0.97 (0.63;1.51)	0.9037	1.09 (0.66;1.78)	0.7393	1.32 (0.84;2.05)	0.2253	1.98 (1.21;3.24)	0.0063	1.20 (0.77;1.86)	0.4231	2.12 (1.23;3.65)	0.0069
**Butter[a]**	**T2**	1.38 (0.87;2.19)	0.1690	1.25 (0.75;2.06)	0.3926	0.90 (0.57;1.42)	0.6381	1.10 (0.66;1.84)	0.7118	1.46 (0.92;2.32)	0.1112	1.54 (0.88;2.69)	0.1339
	**T3**	1.80 (1.12;2.90)	0.0146	1.20 (0.71;2.03)	0.5020	0.93 (0.59;1.49)	0.7689	0.98 (0.58;1.67)	0.9533	1.25 (0.78;2.01)	0.3500	1.59 (0.90;2.80)	0.1101
**Margarine[a]**	**T2**	1.02 (0.64;1.61)	0.9357	0.94 (0.57;1.53)	0.7900	0.91 (0.58;1.44)	0.6826	1.13 (0.69;1.86)	0.6139	0.92 (0.58;1.45)	0.7213	1.31 (0.76;2.26)	0.3328
	**T3**	1.31 (0.83;2.08)	0.2470	0.99 (0.59;1.65)	0.9704	0.95 (0.60;1.49)	0.8155	0.88 (0.53;1.47)	0.6292	0.95 (0.60;1.50)	0.8169	1.20 (0.69;2.08)	0.5174
**Oils[a]**	**T2**	0.84 (0.54;1.30)	0.4297	0.87 (0.53;1.44)	0.5831	1.25 (0.80;1.95)	0.3233	1.10 (0.67;1.79)	0.7079	0.79 (0.51;1.22)	0.2853	0.66 (0.38;1.14)	0.1359
	**T3**	0.65 (0.41;1.03)	0.0674	0.84 (0.51;1.39)	0.4918	1.20 (0.77;1.89)	0.4226	1.05 (0.64;1.75)	0.8404	0.91 (0.57;1.45)	0.6853	1.00 (0.58;1.73)	0.9984
**Dairy[a]**	**T2**	1.19 (0.76;1.85)	0.4531	0.90 (0.56;1.47)	0.6852	1.45 (0.93;2.25)	0.1009	1.24 (0.76;2.01)	0.3826	1.30 (0.84;2.03)	0.2432	1.27 (0.75;2.17)	0.3789
	**T3**	0.76 (0.49;1.19)	0.2307	0.70 (0.43;1.14)	0.1492	0.93 (0.60;1.45)	0.7439	0.76 (0.47;1.25)	0.2794	0.87 (0.56;1.36)	0.5500	0.93 (0.55;1.57)	0.7727
**Sugar-sweetend food[a]**	**T2**	1.18 (0.76;1.81)	0.4617	0.87 (0.53;1.42)	0.5805	1.48 (0.96;2.28)	0.0733	1.38 (0.84;2.25)	0.2011	0.69 (0.44;1.07)	0.0988	0.88 (0.52;1.48)	0.6237
	**T3**	1.01 (0.65;1.59)	0.9546	1.15 (0.71;1.86)	0.5668	1.12 (0.72;1.74)	0.6203	1.44 (0.88;2.34)	0.1443	0.78 (0.50;1.22)	0.2830	0.81 (0.47;1.39)	0.4484
**Caloric drinks[a]**	**T2**	0.95 (0.62;1.46)	0.8189	0.73 (0.44;1.20)	0.2122	1.17 (0.76;1.81)	0.4797	0.71 (0.43;1.17)	0.1843	0.66 (0.42;1.04)	0.0743	0.53 (0.31;0.90)	0.0183
	**T3**	1.09 (0.69;1.72)	0.7041	1.50 (0.92;2.44)	0.1010	1.00 (0.64;1.56)	0.9829	0.93 (0.58;1.50)	0.7620	0.94 (0.60;1.48)	0.7989	0.64 (0.37;1.10)	0.1060
**Water[a] [ml/d]**	**T2**	1.50 (0.95;2.38)	0.0829	0.86 (0.52;1.42)	0.5600	1.08 (0.69;1.70)	0.7259	0.84 (0.51;1.38)	0.4932	1.49 (0.94;2.34)	0.0873	1.79 (1.02;3.14)	0.0410
	**T3**	1.10 (0.66;1.84)	0.7124	0.66 (0.38;1.16)	0.1487	0.87 (0.52;1.44)	0.5805	0.95 (0.55;1.64)	0.8473	1.02 (0.62;1.68)	0.9421	1.78 (0.97;3.25)	0.0621
**Tea[a] [ml/d]**	**T2**	2.24 (1.40;3.60)	**0.0008**	1.85 (1.11;3.08)	0.0191	1.45 (0.92;2.29)	0.1064	1.63 (0.97;2.73)	0.0642	0.96 (0.61;1.52)	0.8607	1.15 (0.66;2.00)	0.6136
	**T3**	1.48 (0.93;2.35)	0.0971	1.10 (0.66;1.83)	0.7133	1.13 (0.71;1.79)	0.6092	1.65 (0.99;2.76)	0.0550	1.07 (0.67;1.70)	0.7838	1.02 (0.58;1.79)	0.9332

Table S15. Sensitivity analysis (176 males at the 15-year follow-up with a BMI <10th or >90th percentile were excluded): Effect estimates and 95% CI assessing the association between tertiles of eating behaviours and dietary intake in males at the 15-year follow-up *(Continued)*

	External eating (N=704)				Emotional eating (N=704)				Dietary restraint (N=704)			
	T2 (n=269) Score=5-9		T3 (n=197) Score=10-22		T2 (n=249) Score=1-3		T3 (n=182) Score=4-23		T2 (n=309) Score=1-3		T3 (n=173) Score=4-21	
	Estimate (95% CI)	P-value	Estimate (95% CI)	P-value	Estimate (95% CI)	P-value	Estimate (95% CI)	P-value	Estimate (95% CI)	P-value	Estimate (95% CI)	P-value
**Total energy[b] [kJ/d]**	193 (-291;678)	0.4341	196 (-340;731)	0.4730	-215 (-696;266)	0.3802	203 (-331;738)	0.4551	-206 (-692;279)	0.4045	-515 (-1,096;66)	0.0822
**Fat[b]**	0.50 (-0.50;1.51)	0.3255	-0.09 (-1.20;1.01)	0.8671	-0.25 (-1.24;0.75)	0.6287	0.43 (-0.68;1.54)	0.4437	0.06 (-0.95;1.06)	0.9111	0.78 (-0.43;1.98)	0.2071
**Protein[b]**	-0.13 (-0.63;0.37)	0.6130	-0.68 (-1.23;-0.13)	0.0163	-0.53 (-1.03;-0.03)	0.0378	-0.35 (-0.90;0.20)	0.2161	-0.02 (-0.52;0.48)	0.9406	0.47 (-0.13;1.08)	0.1235
**Carbohydrate[b]**	-0.35 (-1.61;0.92)	0.5926	0.77 (-0.63;2.17)	0.2813	0.79 (-0.47;2.05)	0.2214	-0.06 (-1.46;1.34)	0.9279	-0.07 (-1.34;1.20)	0.9121	-1.27 (-2.79;0.26)	0.1027
**Fibres[b] [g/d]**	0.49 (-0.60;1.58)	0.3750	-0.59 (-1.79;0.61)	0.3339	-0.02 (-1.10;1.06)	0.9764	-0.89 (-2.09;0.31)	0.1448	0.15 (-0.94;1.24)	0.7825	1.08 (-0.22;2.39)	0.1043
**Total sugar[b]**	-0.41 (-1.81;1.00)	0.5704	1.11 (-0.45;2.66)	0.1621	-0.39 (-1.79;1.01)	0.5857	-0.04 (-1.59;1.52)	0.9633	-0.03 (-1.45;1.38)	0.9616	-0.99 (-2.68;0.70)	0.2505
**SFA[b]**	0.29 (-0.20;0.78)	0.2515	0.01 (-0.53;0.55)	0.9790	-0.20 (-0.69;0.28)	0.4116	0.11 (-0.43;0.65)	0.6804	0.03 (-0.46;0.52)	0.8997	0.31 (-0.28;0.90)	0.2972
**MUFA[b]**	0.24 (-0.19;0.66)	0.2763	-0.05 (-0.52;0.42)	0.8263	-0.19 (-0.61;0.24)	0.3841	0.10 (-0.37;0.57)	0.6696	0.04 (-0.39;0.47)	0.8541	0.32 (-0.19;0.83)	0.2207
**PUFA[c]**	1.00 (0.95;1.04)	0.9493	0.99 (0.95;1.05)	0.8416	1.03 (0.98;1.08)	0.2070	1.04 (0.99;1.09)	0.1387	0.99 (0.95;1.03)	0.6295	1.02 (0.97;1.08)	0.4377
**omega-3 PUFA[c]**	1.04 (1.00;1.08)	0.0747	1.04 (1.00;1.09)	0.0617	1.01 (0.97;1.05)	0.7055	1.04 (1.00;1.09)	0.0583	1.01 (0.97;1.05)	0.6151	1.01 (0.96;1.06)	0.6565
**omega-6 PUFA[c]**	0.99 (0.95;1.04)	0.8124	0.99 (0.94;1.04)	0.6755	1.03 (0.98;1.08)	0.1912	1.04 (0.98;1.09)	0.1763	0.99 (0.94;1.03)	0.5430	1.02 (0.97;1.08)	0.4383

Abbreviations: CI, confidence interval; MUFA, monounsaturated fatty acids; PUFA, polyunsaturated fatty acids; SFA, saturated fatty acids; T2, tertile 2; T3, tertile 3. [a]Effect estimates of multinomial logistic regression are presented as relative risk ratio (95% CI). [b]Effect estimates of multiple linear regression are presented as beta coefficient (95% CI). [c]Effect estimates of multiple linear regression for naturally log-transformed outcome variables are presented as means ratio (95% CI). All models were adjusted for age, BMI, pubertal status, siblings, moderate-to-vigorous physical activity, screen time, total difficulties, parental education, parental BMI, study, and recruitment region. Food groups (except water and tea) and nutrients models were further adjusted for total daily energy intake. Water and Tea models were further adjusted for total daily beverage intake. Tertile 1 is the reference category. Significant associations are marked in bold: p<0.0019.

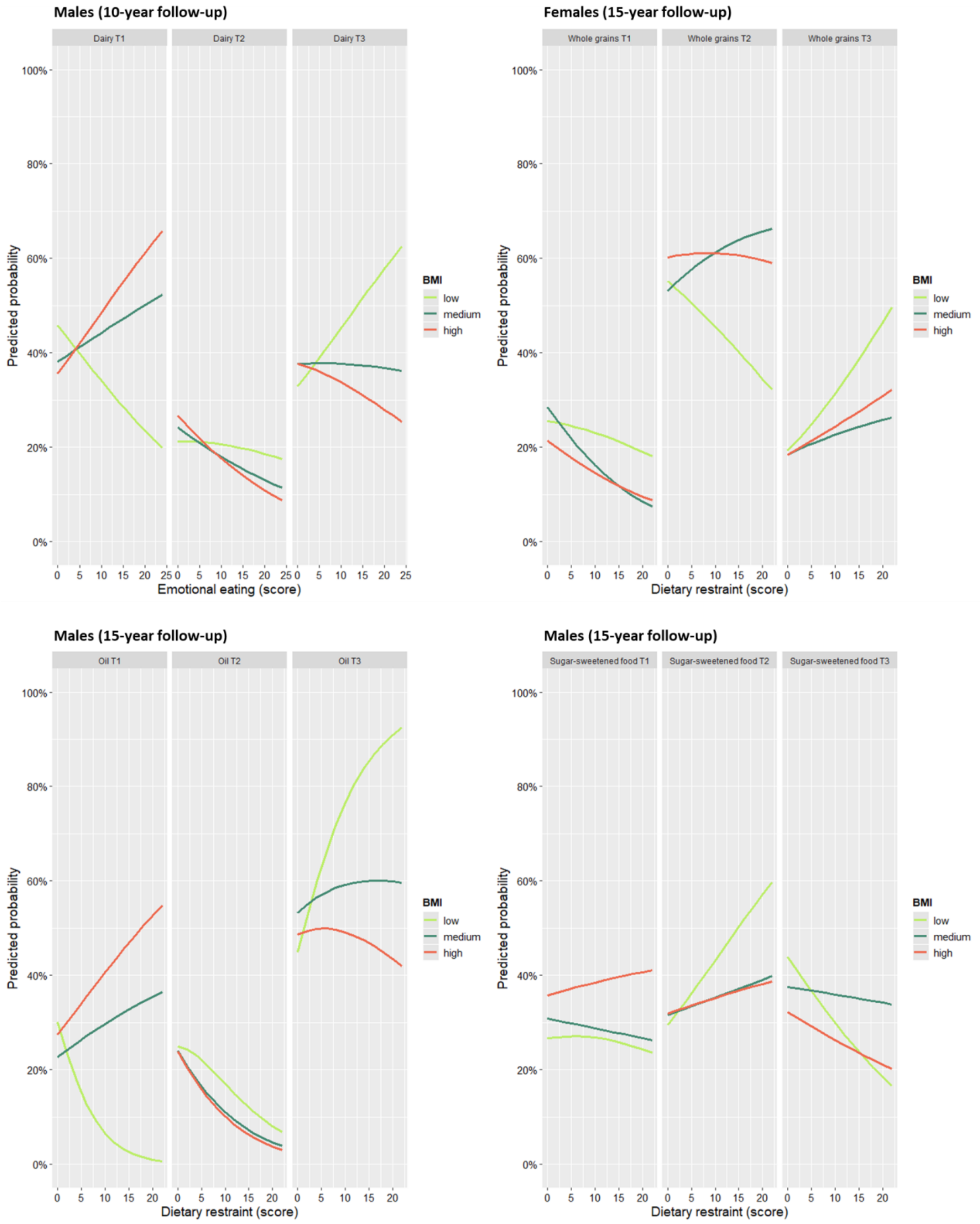

Figure S1. Significant interactions between eating behaviours and BMI for selected food groups

Abbreviations: BMI, body mass index. T1, tertile 1; T2, tertile 2; T3, tertile 3. BMI grouped based on sex- and age-specific percentiles (low: <25th percentile; medium: ≥25th and <75th percentile; high: ≥75th percentile). The plots depict the predicted probability (%) of being in a tertile of dietary intake depending on EWI-C subscale score stratified by BMI.
